# Quantifying the minimum localization uncertainty of image scanning localization microscopy

**DOI:** 10.1016/j.bpr.2024.100143

**Published:** 2024-01-20

**Authors:** Dylan Kalisvaart, Shih-Te Hung, Carlas S. Smith

**Affiliations:** 1 Delft Center for Systems and Control, Delft University of Technology, Delft, the Netherlands; 2 Department of Imaging Physics, Delft University of Technology, Delft, the Netherlands

## Abstract

Modulation enhanced single-molecule localization microscopy (meSMLM), where emitters are sparsely activated with sequentially applied patterned illumination, increases the localization precision over single-molecule localization microscopy (SMLM). The precision improvement of modulation enhanced SMLM is derived from retrieving the position of an emitter relative to individual illumination patterns, which adds to existing point spread function information from SMLM. Here, we introduce SpinFlux: modulation enhanced localization for spinning disk confocal microscopy. SpinFlux uses a spinning disk with pinholes in its illumination and emission paths, to sequentially illuminate regions in the sample during each measurement. The resulting intensity-modulated emission signal is analyzed for each individual pattern to localize emitters with improved precision. We derive a statistical image formation model for SpinFlux and we quantify the theoretical minimum localization uncertainty in terms of the Cramér-Rao lower bound. Using the theoretical minimum uncertainty, we compare SpinFlux to localization on Fourier reweighted image scanning microscopy reconstructions. We find that localization on image scanning microscopy reconstructions with Fourier reweighting ideally results in a global precision improvement of 2.1 over SMLM. When SpinFlux is used for sequential illumination with three patterns around the emitter position, the localization precision improvement over SMLM is twofold when patterns are focused around the emitter position. If four donut-shaped illumination patterns are used for SpinFlux, the maximum local precision improvement over SMLM is increased to 3.5. Localization of image scanning microscopy reconstructions thus has the largest potential for global improvements of the localization precision, where SpinFlux is the method of choice for local refinements.

## Why it matters

One of the main objectives of single-molecule localization microscopy (SMLM) is to improve the precision with which single molecules can be localized. This has been successfully achieved through modulation enhanced SMLM, which uses patterned illumination to increase the information content of signal photons. However, this technique relies on setups with increased technical complexity over SMLM. With SpinFlux, we enable a 2- to 3.5-fold maximum precision improvement over SMLM when the emitter is in the pattern focus. These improvements can be achieved with only minor modifications to existing spinning disk confocal microscopy setups (e.g., a phase mask in the illumination and emission paths). In addition, our modeling framework enables evaluation of a wide variety of spinning disk setups and therefore paves the way for optimal spinning disk design.

## Introduction

Single-molecule localization microscopy (SMLM) increases the precision with which single molecules can be localized beyond the diffraction limit (1,2,3). Methods in SMLM require sparse activation of single emitters, after which emitters can be localized sequentially with reduced uncertainty.

In recent years, various modulation enhanced SMLM (meSMLM) methods were introduced that increase the localization precision over SMLM by sparsely activating emitters with intensity-modulated illumination patterns (4). As a result, information is added to the data about the relative position of the emitter with respect to the illumination patterns. meSMLM methods include SIMFLUX (5), SIMPLE (6), and repetitive optical selective exposure (ROSE) (7), which use sinusoidally shaped intensity patterns, and MINFLUX (8) and RASTMIN (9,10), which use a donut-shaped illumination pattern. Patterned illumination can also be used to improve axial resolution, for example, with modulated localization (ModLoc) (11,12) and ROSE-Z (13), which use illumination with both axial and lateral structure. Additional improvements to the localization precision can be attained through iterative meSMLM (14,15), where patterns are iteratively moved through the sample using prior information from earlier measurements, to improve the localization precision locally around single emitters.

Specifically for SIMFLUX (5), it has been shown that meSMLM with sinusoidal patterns improves the resolution over both SMLM and structured illumination microscopy (SIM) (16). SIM uses nine sinusoidal patterns in total aligned on three lateral axes, and subsequent reconstruction results in at most a 2-fold resolution improvement over the diffraction limit. SIMFLUX on the other hand only uses six patterns in total aligned on two lateral axes, and subsequent localization results in a 2.4-fold maximum improvement of the localization precision over SMLM. Therefore, the combination of structured illumination with sparse localization in meSMLM can result in a better resolution over existing reconstruction approaches, while using less illumination patterns in the process. These factors motivate the incorporation of meSMLM in existing systems, in which image reconstruction instead of localization is the current state-of-the-art.

**Figure 1:**
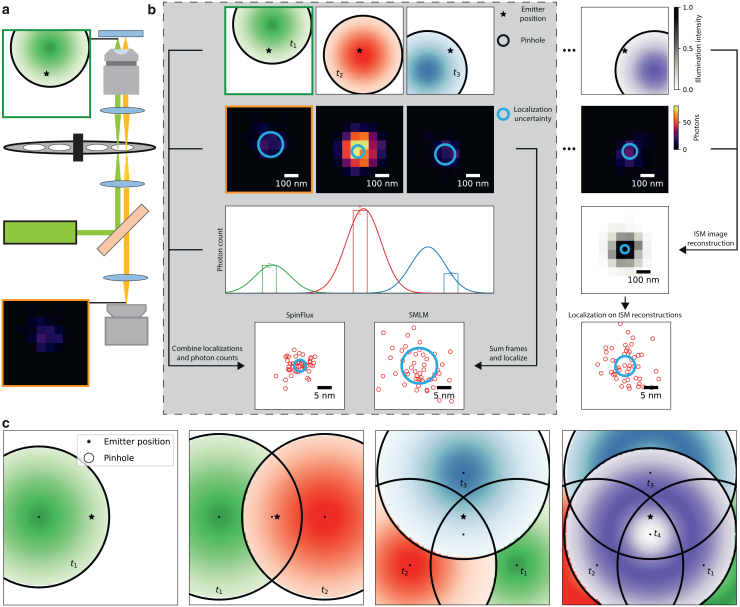
Schematic overview of SpinFlux image formation and analysis. (*a*) In SpinFlux, a rotating disk containing pinholes is placed in the illumination and emission paths. This causes patterned illumination (*green cadre*) in the sample, modulating the emission intensity of emitters in the sample based on their relative distance to the pattern. Subsequently, the emission signal (*orange cadre*) is windowed by the pinhole. Rapidly switching the laser on and off causes stroboscopic illumination of emitters in the sample with stationary illumination patterns. (*b*) SpinFlux obtains its localization precision improvement by merging localized emitter data with information about the relative distance between an illumination pattern and the emitter, derived from photon counts. In this way, it improves the localization precision over SMLM, which only uses localized emitter data and ignores pattern information. We compare SpinFlux with an idealized approach, in which first an ISM acquisition and reconstruction are performed. Afterward, isolated emitters are localized in the ISM reconstruction. (*c*) Schematic overview of SpinFlux localization variants. In the main text, we consider SpinFlux with one, two, three, and four sequentially applied illumination patterns. The configurations with one, two, and three patterns use Gaussian beams, the configuration with four patterns uses donut beams. Additional configurations are explored in the supporting document.

A promising candidate system is spinning disk confocal microscopy (SDCM) (17,18,19,20,21) (see Fig. 1 *a*). SDCM introduces a spinning disk with pinholes in the illumination and emission paths. Rapidly pulsing the excitation laser causes stroboscopic illumination of the sample with moving illumination foci. If used for image scanning microscopy (ISM) (22), the fluorescent emission signal is recorded on an image detector. Subsequent reconstruction of the recorded images results in an expected resolution improvement of a factor 2 over diffraction limited imaging (18,19).

Recently, SDCM was used for PAINT- and STORM-based localization microscopy, where SMLM localization algorithms were used to localize emitters in raw camera data (20,21). It is shown that this improves the detection rate and signal-to-background ratio compared with widefield SMLM at the cost of a reduced signal photon count, resulting in a localization precision that is at best comparable with that of SMLM (20).

However, these methods do not take the information contained in the illumination pattern into account, as one would do in meSMLM. In this text, we therefore develop a statistical image formation model, suited for modulation enhanced localization in SDCM (see Fig. 1 *b*). Our method, called SpinFlux, sequentially applies patterned illumination generated by a spinning disk to excite the sample. Subsequently, emitters are localized in the recordings from a sequence of individual pattern acquisitions, taking knowledge about the pattern into account. The resulting intensity-modulated emission signal is then described by our image formation model. To evaluate the potential localization precision improvements of SpinFlux, we need to study the information contained in a single-pattern exposure, the localization precision obtained by sequential illumination with multiple patterns and the optimal pattern configuration to maximally improve the precision. To accomplish this, we calculate the theoretical minimum uncertainty of SpinFlux in terms of the Cramér-Rao lower bound (CRLB) (23,24). The CRLB is often used in (me)SMLM to quantify the theoretical minimum uncertainty of localizations. Using the SpinFlux image formation model, we calculate the CRLB for various illumination pattern configurations. Based on the CRLB, we compare SpinFlux with SMLM.

Secondly, we consider a localization approach that is comparable with SpinFlux. Here, isolated emitters are localized directly in ISM reconstructions (25) rather than in individual pattern acquisitions as done in SpinFlux. Specifically, we consider localization in ISM reconstructions with a factor $\sqrt{2}$-reduction in the point spread function (PSF) width. We also consider ISM reconstructions that are Fourier reweighted (see Fig. 1 *b*), resulting in a factor 2 reduction in the PSF width. We approximate the maximum localization precision of these approaches and compare it with SpinFlux.

## Methods

In SpinFlux (see Fig. 1 *a*), a spinning disk containing pinholes is placed in the illumination and emission paths. The spinning disk is rotated, thereby sequentially moving illumination patterns over the sample. As in SDCM (19), the excitation laser is rapidly switched on and off. Within the time frame where the laser is on, the spinning disk can be considered stationary. This causes stroboscopic illumination of emitters in the sample. Furthermore, the illumination has a nonuniform intensity profile over the field of view due to the spinning disk architecture. This causes patterned illumination of emitters in the sample, which in turn results in intensity modulation of the emission signal. The rotation angle of the spinning disk determines the position of each illumination pattern with respect to the emitter position. Subsequently, the intensity-modulated emission signal is windowed by the same pinhole, after which the signal is imaged on a camera.

The image analysis (see Fig. 1 *b*) consists of extracting localized emitters from the recordings, as well as retrieving the relative distance between the illumination pattern and emitter from the photon count. To evaluate the total amount of information that can be extracted from the measurements with this approach, we first develop an image formation model for SpinFlux. We subsequently use this model to calculate the theoretical minimum uncertainty of SpinFlux in terms of the CRLB. The CRLB will allow us to quantify the maximum amount of information contained in each exposure with a single pattern. In turn, we use this to derive the localization precision that can be attained through sequential exposures with multiple patterns. In addition, we can explore how the pattern configuration, the pinhole radius, and the mutual spacing between patterns affect the maximum localization precision.

### Model for SpinFlux image formation

To calculate the theoretical minimum uncertainty that can be attained with SpinFlux localization, we need a model to describe the amount of photons collected by a camera pixel. Existing models for (me)SMLM (5,8,14,26,27) do not suffice for this, as they do not include a pinhole in the illumination and emission paths. In this subsection, we therefore develop a statistical image formation model for SpinFlux. A detailed derivation of this model can be found in Note S2.

For the image formation, we assume that pinholes are separated far enough on the spinning disk, such that only one pinhole can appear in a region of interest during each camera frame. This assumption is valid for the magnifications, pinhole sizes, and pinhole separations in existing SDCM setups (19,20,21). In line with this, we can assume that there is no cross talk of emission signals between different pinholes. This allows us to describe the regions of interest on the camera frames as separate regions of interest from individual patterns.

We model the pinhole in the emission path as a circular window. In the absence of readout noise, the measurements on each camera pixel can be described as independent realizations of a Poisson process (26). For each pixel *i* with center coordinates $\left(x_{i},y_{i}\right)$ and for the measurement corresponding to illumination pattern *k*, the expected photon count $\mu_{i,k}$ after illumination through the pinhole with position $\left(x_{p,k},y_{p,k}\right)$ is described by (see Note S2):

(1) $$\mu_{i,k}\left(x_{i},x_{p,k},y_{i},y_{p,k}\right)=A\theta_{I}P\left(\theta_{x}-x_{p,k},\theta_{y}-y_{p,k}\right)H\left(\theta_{x},\theta_{y},x_{i},y_{i}\right)+A\theta_{b}B_{i,k}\text{.}$$

Here, $\left(\theta_{x},\theta_{y}\right)$ is the emitter position, $\theta_{I}$ is the expected signal photon count under maximum illumination, and $\theta_{b}$ is the expected background photon count.

Each illumination pattern $P\left(\theta_{x}-x_{p,k},\theta_{y}-y_{p,k}\right)$ is assumed to be a known function with a known pinhole position $\left(x_{p,k},y_{p,k}\right)$ in our image formation model. We model each illumination pattern as a Gaussian PSF in the center of the pinhole, with standard deviation $\sigma_{\text{illum}}$. Alternate illumination patterns can be generated by placing a phase mask in the illumination path. We therefore also include a model of the donut-shaped pattern from, e.g., MINFLUX (8), with a zero-intensity minimum at the center of the pinhole and standard deviation $\sigma_{\text{illum}}$.

Note that the signal photon budget of a single emitter stays constant when going from one pattern location to multiple pattern locations. In particular, this means that one pattern exhausts the full signal photon budget, whereas multiple patterns need to share the same signal photon budget. Each pattern in a multiple-pattern illumination sequence gets a share of the signal photon budget proportional to their illumination intensity on the emitter position.

We model the emission PSF as a Gaussian, with standard deviation $\sigma_{\text{PSF}}$. The term $H\left(\theta_{x},\theta_{y},x_{i},y_{i}\right)$ describes the discretized emission PSF after windowing by the pinhole (see Note S2: illumination and emission point spread functions).

In existing work on meSMLM, such as in MINFLUX (8), it is assumed that meSMLM is able to record the same amount of signal photons as SMLM. This assumption allows benchmarking between methods on the same signal photon count. However, the assumption is not trivial, as additional illumination power or time is needed to exhaust the signal photon budget with nonmaximum illumination intensity. Properly adjusting the illumination power to compensate for the reduced photon flux requires accurate prior knowledge about the emitter position, which is generally unavailable. Increasing the illumination time increases the probability of sample degradation. As such, we should include the possibility that meSMLM will not exhaust the signal photon budget in the image formation model.

The normalizing constant *A* describes how the signal photon budget is affected by nonmaximum illumination intensity. This constant plays a vital role in benchmarking meSMLM (when the summed intensity over all patterns does not result in a uniform profile), as it gives a physical explanation of the fair signal photon count against which meSMLM should be compared (14). Specifically when comparing meSMLM to SMLM, the normalization constant models whether meSMLM would have had recorded the same amount of signal photons as SMLM, despite the additional illumination power or time needed to do so. Results on the improvement of meSMLM compared with SMLM should thus only be given in the context of the normalizing constant *A*.

We choose *A* to model two scenarios (see Note S2: multiple emission patterns). In the first scenario, which we explore in this text, we assume that the entire signal photon budget is exhausted after illumination with all patterns, independent of the total brightness on the emitter position. We thus assume the illumination power and time is sufficient to exhaust the signal photon budget of the emitter. Here, *A* is inversely proportional to the summed illumination patterns. The only signal photon loss in this scenario comes from the windowing effect of the emission pinhole. This scenario is consistent with the assumption used in, e.g., MINFLUX (8), stating that meSMLM will record the same amount of photons as SMLM. In the second scenario, the illumination power and time are constant for each pattern such that the total illumination power and time equal that of SMLM, even though this does not exhaust the signal photon budget for nonmaximum illumination. Instead, the maximum possible signal photon count occurs when the emitter is placed at the brightest position of the total illumination pattern. Here, *A* is inversely proportional to the amount of illumination patterns *K*.

The constant $B_{i,k}$ describes how the background is affected by illumination pattern *k*. As such, the term $A\theta_{b}B_{i,k}$ represents the effective background under patterned illumination. It depends on the camera pixel area, the pinhole area, the PSF, and the illumination pattern, but not on the emitter position (see Note S2: effective background $B_{i}$). In the analysis of, e.g., MINFLUX (8), the pattern dependency of the background is neglected. We can incorporate this in our image formation model for SpinFlux by modeling $B_{i,k}$ as the overlapping area between the camera pixel *i* and the approximation of pinhole *k* (see Note S2: pattern-independent background).

### Cramér-Rao lower bound

To quantify the theoretical minimum uncertainty of localizations, the CRLB is often used (23,24). Under regularity conditions on the likelihood of the data (23), the CRLB states that the estimator covariance $C_{\hat{\theta }}$ of any unbiased estimator $\hat{\theta }$ of the parameters θ satisfies the property that $\left(C_{\hat{\theta }}-I^{-1}\left(\theta \right)\right)$ is positive semidefinite. Here, I(θ) is the Fisher information, of which entry (u,v) is described by:

(2) $$I_{uv}\left(\theta \right)=E\left[\frac{\partial l\left(\theta |c\right)}{\partial \theta_{u}}\frac{\partial l\left(\theta |c\right)}{\partial \theta_{v}}\right]\text{,}$$

where l(θ|c) is the log-likelihood function given the recorded photon counts c on the camera pixels. The matrix $I^{-1}\left(\theta \right)$ is the CRLB. Consequently, the diagonal of the CRLB bounds the estimator variance from below. Specifically for SMLM, the CRLB is attained by the covariance of the maximum likelihood estimator for 100 or more signal photons (26). As the localization uncertainty of the maximum likelihood estimator converges asymptotically to the CRLB (28, 29), we can also use the CRLB to investigate the theoretical minimum uncertainty of SpinFlux.

Using the image formation model from Eq. 1, we can derive the CRLB for SpinFlux. When using *K* pinholes and a camera consisting of an array with $N_{\text{pixels}}$ pixels, any entry (u,v) of the Fisher information is given by (see Note S3):

(3) $$I_{uv}\left(\theta \right)=\sum_{i=1}^{N_{\text{pixels}}}\sum_{k=1}^{K}\frac{1}{\mu_{i,k}}\frac{\partial \mu_{i,k}}{\partial \theta_{u}}\frac{\partial \mu_{i,k}}{\partial \theta_{v}}\text{.}$$

To evaluate Eq. 3, the partial derivatives of the image formation model of Eq. 1 with respect to the unknown parameters $\theta_{x}$, $\theta_{y}$, $\theta_{I}$, and $\theta_{b}$ need to be computed. Expressions for these partial derivatives are found in Note S4.

### Simulations and parameter values

We sampled measurements from the image formation model and evaluated the CRLB using representative in silico experiments. The model parameters (see Table S1) are considered to be representative of an SDCM experiment (20).

To maximize the information contained in the Gaussian illumination and emission PSFs, we choose their standard deviations to be diffraction limited (30). Specifically, we approximate the standard deviation of the illumination as $\sigma_{\text{illum}}=0.21\frac{\lambda_{\text{ex}}}{\text{NA}}$ and the standard deviation of the PSF as $\sigma_{\text{PSF}}=0.21\frac{\lambda_{\text{em}}}{\text{NA}}$. Here, $\lambda_{\text{ex}}$ and $\lambda_{\text{em}}$, respectively, describe the excitation and emission wavelengths and NA is the numerical aperture.

Emitters are located in the center of the region of interest, consisting of 10 × 10 pixels. The pinhole was discretized on a mesh with $N_{M,x}$, $N_{M,y}=100$ pixels in each direction. For $N_{M,x}$, $N_{M,y}=100$ mesh pixels, the relative error in the CRLB caused by the discretized pinhole approximation is at most 0.02% (see Fig. S2).

## Results

A spinning disk can be designed with various pinhole sizes, spacing, and arrangements (20). In addition, the rotation of the spinning disk gives additional freedom, as patterns and pinholes can appear arbitrarily close to each other via sequential illumination with a rotating spinning disk. For SpinFlux, this means that a wide variety of illumination pattern configurations can be created via the appropriate spinning disk and rotation angle. Furthermore, donut-shaped illumination patterns can be used by adding a phase mask in the illumination path (see Fig. S3). In this section, we explore how the theoretical minimum localization uncertainty of SpinFlux depends on pattern configurations and positions.

**Figure 2:**
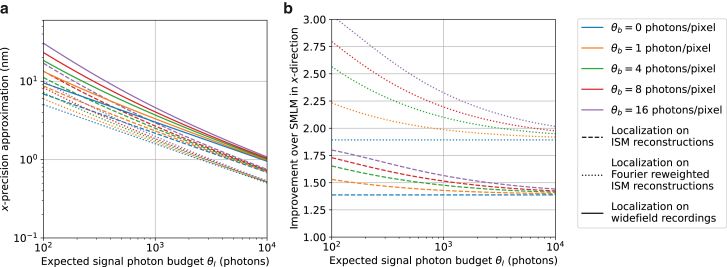
Approximation of the theoretical minimum localization uncertainty of SMLM on reconstructions acquired from (Fourier reweighted) ISM. For this simulation, a PSF standard deviation of 93.3 nm and a camera pixel size of 65 nm were used. (*a*) Approximate CRLB in the *x*-direction as a function of the expected signal photon budget for varying values of the expected background photon count. (*b*) Improvement of the approximate CRLB over SMLM as a function of the expected signal photon budget for varying values of the expected background photon count.

**Figure 3:**
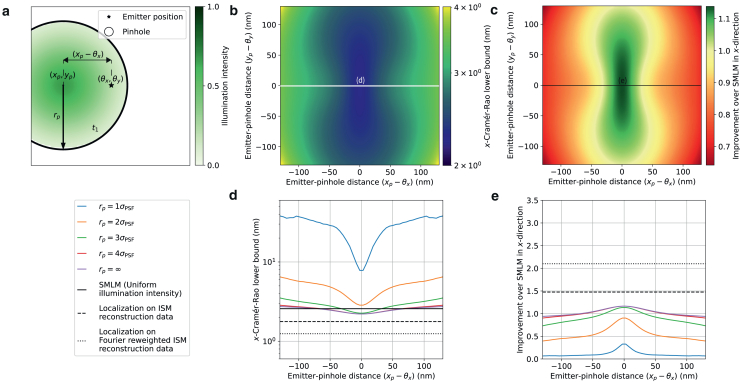
Theoretical minimum localization uncertainty of SpinFlux localization with one *x*-offset pinhole and pattern. For this simulation, 2000 expected signal photons and 8 expected background photons per pixel were used. Results are evaluated for the scenario where the entire signal photon budget is exhausted after illumination with the pattern (disregarding signal photons blocked by the spinning disk). (*a*) Schematic overview of SpinFlux localization with one pinhole with radius $r_{p}$ centered at coordinates $\left(x_{p},y_{p}\right)$. In (*d*) and (*e*), the *x*-distance $\left(x_{p}-\theta_{x}\right)$ between the pinhole and the emitter is varied, where $y_{p}=\theta_{y}$. (*b*) SpinFlux CRLB in the *x*-direction as a function of the emitter-pinhole *x*- and *y*-distances for pinhole radius $r_{p}=3\sigma_{\text{PSF}}$. (*c*) Improvement of the SpinFlux CRLB over SMLM as a function of the emitter-pinhole *x*- and *y*-distances, for pinhole radius $r_{p}=3\sigma_{\text{PSF}}$. (*d*) CRLB in the *x*-direction as a function of the emitter-pinhole *x*-distance. Simulations show SpinFlux with varying pinhole sizes, widefield SMLM, and localization on ISM reconstructions. (*e*) Improvement of the SpinFlux CRLB over SMLM as a function of the emitter-pinhole *x*-distance for varying pinhole sizes.

**Figure 4:**
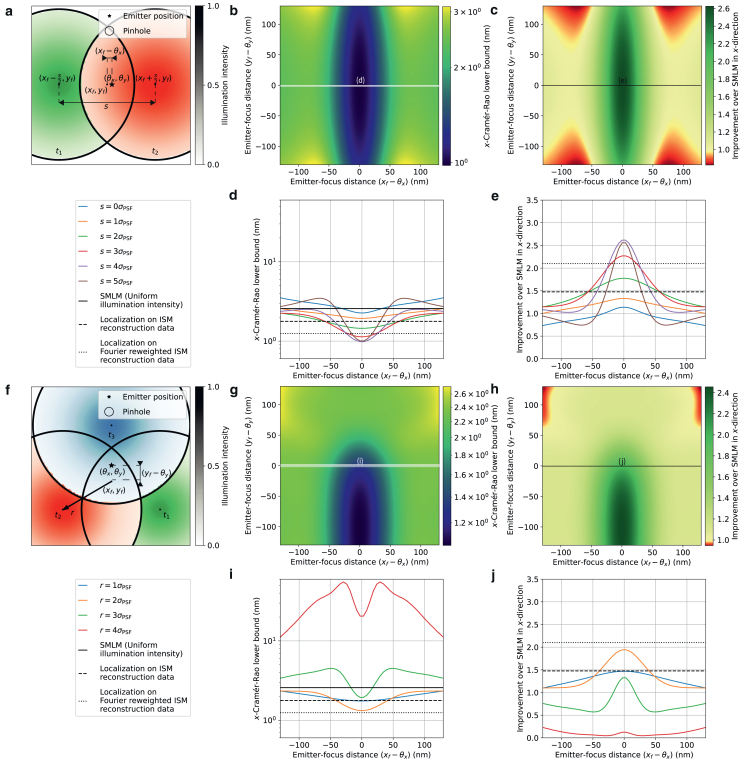
Theoretical minimum localization uncertainty of SpinFlux localization with multiple pinholes and patterns. For this simulation, 2000 expected signal photons and 8 expected background photons per pixel were used, with pinhole radius $r_{p}=3\sigma_{\text{PSF}}$. Results are evaluated for the scenario where the entire signal photon budget is exhausted after illumination with all patterns (disregarding signal photons blocked by the spinning disk). (*a*) Schematic overview of SpinFlux localization with two pinholes, separated in *x* by distance *s* and centered around the focus coordinates $\left(x_{f},y_{f}\right)$. In (*d*) and (*e*), the *x*-distance $\left(x_{f}-\theta_{x}\right)$ between the pattern focus and the emitter is varied, where $y_{f}=\theta_{y}$. (*b*) SpinFlux CRLB in the *x*-direction as a function of the emitter-pinhole *x*- and *y*-distances for pinhole separation $r_{p}=4\sigma_{\text{PSF}}$. (*c*) Improvement of the SpinFlux CRLB over SMLM as a function of the emitter-pinhole *x*- and *y*-distances for pinhole separation $r_{p}=4\sigma_{\text{PSF}}$. (*d*) CRLB in the *x*-direction as a function of the emitter-focus *x*-distance. Simulations show SpinFlux with varying pinhole separations, widefield SMLM, and localization on ISM reconstructions. (*e*) Improvement of the SpinFlux CRLB over SMLM as a function of the emitter-focus *x*-distance for varying pinhole separations. (*f*) Schematic overview of SpinFlux localization with a triangle of three pinholes, centered at focus coordinates $\left(x_{f},y_{f}\right)$ at a radius *r*. In (*i*) and (*j*), the *x*-distance $\left(x_{f}-\theta_{x}\right)$ between the pattern focus and the emitter is varied, where $y_{f}=\theta_{y}$. (*g*) SpinFlux CRLB in the *x*-direction as a function of the emitter-pinhole *x*- and *y*-distances for pinhole spacing $r=2\sigma_{\text{PSF}}$. (*h*) Improvement of the SpinFlux CRLB over SMLM as a function of the emitter-pinhole *x*- and *y*-distances for pinhole spacing $r=2\sigma_{\text{PSF}}$. (*i*) CRLB in the *x*-direction as a function of the emitter-focus *x*-distance. Simulations show SpinFlux with varying pinhole spacing, widefield SMLM, and localization on ISM reconstructions. (*j*) Improvement of the SpinFlux CRLB over SMLM as a function of the emitter-focus *x*-distance for varying pinhole spacing.

**Figure 5:**
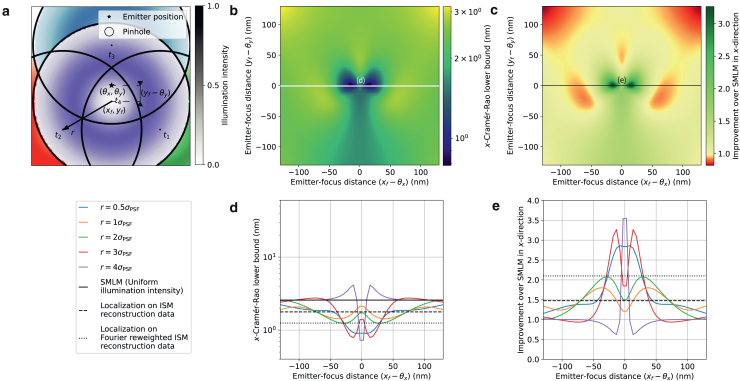
Theoretical minimum localization uncertainty of SpinFlux localization with four pinholes and donut-shaped patterns in an equilateral triangle configuration with a center pinhole. For this simulation, 2000 expected signal photons and 8 expected background photons per pixel were used, with pinhole radius $r_{p}=3\sigma_{\text{PSF}}$. Results are evaluated for the scenario where the entire signal photon budget is exhausted after illumination with all patterns (disregarding signal photons blocked by the spinning disk). (*a*) Schematic overview of SpinFlux localization with a triangle of three pinholes with an additional center pinhole centered at focus coordinates $\left(x_{f},y_{f}\right)$. In (*d*) and (*e*), the *x*-distance $\left(x_{f}-\theta_{x}\right)$ between the pattern focus and the emitter is varied, where $y_{f}=\theta_{y}$. (*b*) SpinFlux CRLB in the *x*-direction as a function of the emitter-pinhole *x*- and *y*-distances for pinhole spacing $r=3\sigma_{\text{PSF}}$. (*c*) Improvement of the SpinFlux CRLB over SMLM as a function of the emitter-pinhole *x*- and *y*-distances for pinhole spacing $r=3\sigma_{\text{PSF}}$. (*d*) CRLB in the *x*-direction as a function of the emitter-focus *x*-distance. Simulations show SpinFlux with varying pinhole spacing, widefield SMLM, and localization on ISM reconstructions. (*e*) Improvement of the SpinFlux CRLB over SMLM as a function of the emitter-focus *x*-distance for varying pinhole spacing.

In Figure 2, Figure 3, Figure 4, Figure 5, and S4–S17, we calculate the theoretical minimum uncertainty for the scenario where the entire signal photon budget is exhausted after illumination with all patterns. We compute the theoretical minimum localization uncertainty for three standard configurations. These pattern configurations can be created via sequential illumination with a rotating spinning disk, where the rotation angle of the spinning disk determines the position of an illumination pattern. In Localization on ISM reconstruction data, we establish localization on ISM reconstruction data as a benchmark for SpinFlux. In Single-pattern configuration, we simulate the theoretical minimum uncertainty using a single pattern and pinhole, akin to confocal microscopy. In Two-pattern configuration, we compute the CRLB for a two-pattern configuration where pinholes are separated by a distance *s* along the *x*-axis, resembling raster-like configurations of earlier work on meSMLM (9,10,14). In Triangular pattern configuration, patterns and pinholes are arranged in an equilateral triangle configuration, similar to the configuration found in MINFLUX (8,15). Donut-shaped intensity patterns shows the effect of donut-shaped illumination patterns. A summary of the most important simulation results is found in Table 1.

**Table 1:** Summary of simulation results for localization on ISM reconstructions and SpinFlux variants considered in the main text

Variant	Amount of patterns	Illumination type	Maximum *x*-improvement
ISM reconstructions	≫4	Gaussian	1.48
Fourier reweighted ISM reconstructions	≫4	Gaussian	2.10
SpinFlux	1	Gaussian	1.17
	2	Gaussian	2.62
	3	Gaussian	1.94
	4	Donut	3.50

To rigorously quantify the improvement of SpinFlux, we also evaluate the localization precision in the two other scenarios described in Model for SpinFlux image formation. Figs. S18–S31 show the theoretical minimum uncertainty in the case in which the illumination power and time are constant for each pattern. There, the maximum possible signal photon count occurs when the emitter is placed at the brightest position of the total illumination pattern. Figs. S32–S45 show the CRLB where the pattern dependency of the background is neglected and where the entire signal photon budget is exhausted after illumination with all patterns.

### Localization on ISM reconstruction data

As a straightforward implementation of localization, we consider localizing isolated emitters in ISM reconstruction data. In this approach, an ISM image is first acquired and reconstructed, resulting in a reduction of the PSF width by at most a factor $\sqrt{2}$ (18,19). If the ISM image is subsequently Fourier reweighted (18), the PSF width is reduced further by a total factor 2. Subsequently, individual emitters are localized in the ISM reconstruction data.

We approximate the CRLB for this localization approach (see Note S1). For a signal photon count of 2000 photons per emitter and a background photon count of 8 photons per pixel, the best-case localization precision of localization on the ISM reconstructions is 1.77 nm, or 1.25 nm with Fourier reweighting, whereas SMLM would achieve a localization precision of at most 2.62 nm. The improvement of localization on the ISM reconstructions over SMLM is thus 1.48, or 2.10 with Fourier reweighting. These results agree with the improvements that were recently found experimentally (25).

Fig. 2 shows the localization precision of localization of individual emitters in the ISM data over a range of signal and background photon counts, PSF standard deviations, and camera pixel sizes. From Fig. 2 *b*, we see that the improvement of localization on the ISM data over SMLM for a PSF standard deviation of 93.3 nm and a camera pixel size of 65 nm is at most 1.8, or 3.0 with Fourier reweighting. This is achieved at a signal photon count of 200 photons and a background photon count of 16 photons per pixel. Furthermore, the improvement decreases to 1.4, or 1.9 with Fourier reweighting, as the background goes to zero. For zero background, the improvement over SMLM is constant as a function of the signal photon count. In our approximation, the localization precision of localization on ISM reconstructions is proportional to $\frac{1}{\sqrt{\theta_{I}}}$ if the background is zero, and therefore the improvement over widefield SMLM is constant.

Fig. S1 shows the localization precision of localization of individual emitters in the ISM data over a range of PSF standard deviations and camera pixel sizes. From Fig. S1 *b*, we see that the improvement of localization on the ISM data over SMLM for a signal photon count of 2000 photons and a background photon count of 8 photons per pixel is at most 1.7, or 2.8 with Fourier reweighting, achieved at a PSF standard deviation of 250 nm and a camera pixel size of 50 nm. Furthermore, the improvement decreases to 1.3, or 1.5 with Fourier reweighting, for an increasing camera pixel size and a decreasing PSF size.

### Single-pattern configuration

In Fig. 3, we evaluate the theoretical minimum uncertainty in the case in which a single pinhole is used for illumination and emission, as illustrated in Fig. 3 *a*. Results are shown for the scenario where the entire signal photon budget is exhausted after illumination with all patterns.

From Fig. 3, *d* and *e*, we see that the localization precision is optimal when the pinhole and pattern are centered directly on the emitter position. Without a pinhole, this results in an improvement of at most 1.17 over SMLM. For a pinhole with radius $r_{p}=4\sigma_{\text{PSF}}$, the difference with SMLM is negligible, indicating that the confocal effect of the pinhole has been lost. The improvement can thus be attributed to the effect of pattern-dependent background, as the background is reduced on camera pixels that are not located on the maximum of the Gaussian illumination pattern. This background reduction is visualized in Fig. S4 *g*, showing a 10.2-fold reduction in the average background count per pixel compared with SMLM for $r_{p}=4\sigma_{\text{PSF}}$ and $x_{p}=\theta_{x}$.

For pinholes of radius $r_{p}=3\sigma_{\text{PSF}}$ and below, the localization precision deteriorates with respect to the no-pinhole case. Already for $r_{p}=2\sigma_{\text{PSF}}$, no position of the pinhole results in an improvement over SMLM. In these cases, the pinhole not only blocks background photons, but also signal photons carrying information about the emitter position. Fig. S4, *f* and *g* show that, in the best case (for $x_{p}=\theta_{x}$), 248 signal photons are lost when going from $r_{p}=3\sigma_{\text{PSF}}$ to $r_{p}=2\sigma_{\text{PSF}}$, whereas the average background is reduced with only 0.21 photons per pixel. As such, more information about the emitter position is lost due to the loss of signal photons than that we gain by blocking background, resulting in a reduction of the improvement factor from 1.14 to 0.90. Similarly, moving the pinhole away from the emitter position blocks signal photons, thereby reducing the localization precision. For $r_{p}=3\sigma_{\text{PSF}}$, the improvement over SMLM goes from 1.14 at $x_{p}=\theta_{x}$ to 0.73 at a 130 nm distance between $x_{p}$ and $\theta_{x}$. From this, we can conclude that larger pinholes are in principle better for SpinFlux, as more information about the underlying signal is revealed through the larger pinhole.

### Two-pattern configuration

In Figs. 4 and S6, we evaluate the theoretical minimum uncertainty in the case in which multiple patterns are used sequentially for illumination and emission. We first consider the scenario of pinholes that are separated in the *x*-direction around focus coordinates $\left(x_{f},y_{f}\right)$, as illustrated in Fig. 4, *a*–*e*. Results are shown for the scenario where the entire signal photon budget is exhausted after illumination with all patterns. For these simulations, the pinhole radius was set to $r_{p}=3\sigma_{\text{PSF}}$ for both pinholes.

From Fig. 4, *d* and *e*, we see that using multiple patterns is beneficial for SpinFlux, maximally resulting in a 2.62-fold precision improvement over SMLM in the *x*-direction when using a pinhole separation $s=4\sigma_{\text{PSF}}$. This improvement decreases only moderately to 2.17 when the pattern *y*-coordinate is moved 130 nm out of focus (see Fig. S7). When the illumination time and power are adjusted to exhaust the entire signal photon budget, the low-intensity tails of the Gaussian intensity profile increase the information content of signal photons, as these contain increased information about the relative position of the emitter with respect to the illumination pattern. As discussed in Model for SpinFlux image formation, the multiple-pattern configuration has the same signal photon budget as the single-pattern configuration. These results therefore show that the same signal photon budget is utilized more efficiently by using multiple pattern locations.

However, increasing the pinhole separation also reduces the region where SpinFlux improves over SMLM. For a pinhole separation $s=3\sigma_{\text{PSF}}$, the domain where SpinFlux improves over SMLM by at least a factor 1.2 spans 175 nm, whereas this domain spans 111 nm for $s=4\sigma_{\text{PSF}}$. In the case where the pinholes are not centered around the emitter position, one of the patterns takes more of the signal photon budget than the other. As such, highly informative signal photons carrying information from the tails of the Gaussian illumination pattern are traded in for lowly informative photons coming from the center of the pattern. This is shown in Fig. S6 *f*: for a pinhole separation $s=4\sigma_{\text{PSF}}$, 1573 signal photons are collected in total when $x_{f}=\theta_{x}$, with the remaining 427 photons being blocked by the spinning disk. When considering a 130 nm distance between $x_{f}$ and $\theta_{x}$, 1956 signal photons are being collected in total as one pinhole has moved close to the emitter position. Yet these photons are lowly informative, resulting in a precision improvement of 1.09 over SMLM. For increasing separations, the relative difference in illumination intensity between noncentered patterns increases, thereby reducing the domain of improvement.

Furthermore, Fig. 4, *d* and *e* show that there is an optimal pinhole separation of $s=4\sigma_{\text{PSF}}$ for SpinFlux. When increasing the pinhole separation beyond this, the localization precision decreases again. This is caused by a combination of two factors. First of all, as shown in Fig. S6 *f*, the spinning disk blocks an increasing amount of signal photons for increasing pinhole separations, as the overlap between the pinhole and emission PSF is reduced. Between $s=4\sigma_{\text{PSF}}$ and $s=5\sigma_{\text{PSF}}$, the amount of signal photons is reduced by 324 when $x_{f}=\theta_{x}$. This effect is eliminated when the pinhole is removed, as shown in Fig. S8.

Secondly, increasing the pinhole separation results in illumination with the low-intensity tails of the Gaussian illumination patterns. As we exhaust the signal photon budget in this scenario and as the background is pattern dependent, this results in an amplification of the background. Fig. S6 *g* shows that the average background count increases from 7.75 photons per pixel at $s=4\sigma_{\text{PSF}}$ to 26.7 photons per pixel at $s=5\sigma_{\text{PSF}}$.

Up until now, we have only considered the localization precision in the *x*-direction. Because the pattern has a different structure in the *x*- and *y*-directions, the modulated emission intensity will carry different information about the emitter *x*- and *y*-positions. Specifically in this configuration, both patterns lie on the *x*-axis. Therefore, the intensity difference in the modulated emission signal is strongly affected by the emitter *x*-position. However, as both patterns have the same *y*-coordinate, there is no difference in the effect of the emitter *y*-coordinate on the modulated emission intensity between the patterns. Therefore minimal information is carried about the emitter *y*-position.

To investigate how the two-pattern configuration of Fig. 4 *a* affects the *y*-precision, we equivalently consider the *x*-precision that can be obtained with the rotated pattern (see Fig. S9). From Fig. S9, we see that the *x*-precision for the rotated pattern results in negligible improvements or even reductions over SMLM if the entire signal photon budget is exhausted. Specifically for $s=4\sigma_{\text{PSF}}$, the improvement factor over SMLM is 0.83 when the patterns are perfectly centered around the emitter position, whereas the improvement increases to 1.12 when the distance between $y_{f}$ and $\theta_{y}$ is 130 nm. From the equivalence, we can thus conclude that the two-pattern configuration of Fig. 4 *a* results in optimal *x*-precision, but the associated *y*-precision is diminished.

### Triangular pattern configuration

In Figs. 4, *f*–*j* and S10, we evaluate the theoretical minimum uncertainty in the case in which multiple pinholes are used for illumination and emission in an equilateral triangle configuration centered around focus coordinates $\left(x_{f},y_{f}\right)$. Results are shown for the scenario where the entire signal photon budget is exhausted after illumination with all patterns. For these simulations, the pinhole radius was set to $r_{p}=3\sigma_{\text{PSF}}$ for all pinholes.

From Fig. 4, *i* and *j*, we see that the triangle configuration from Fig. 4 *f* results in a precision improvement in the *x*-direction of at most 1.94 compared with SMLM, when the distance between the pinholes and the center of the triangle is $r=2\sigma_{\text{PSF}}$. As seen for the two-pattern case, this optimum is a result of two contrasting factors. On one hand, increasing the pattern distance illuminates the emitter with the tail of the Gaussian intensity profile, thereby increasing the information that signal photons carry about the relative distance between the illumination pattern and the emitter. On the other hand, increasing the distance between the emitter and the pinholes also increases the amount of signal photons that are blocked by the spinning disk, while the pattern-dependent background increases due to the low illumination intensity.

Note that the *x*-localization precision of the triangle configuration is worse than that of the two-pattern configuration described in Two-pattern configuration. The reason for this is that the triangle configuration contains one pinhole, of which the *x*-coordinate is located close to the true emitter *x*-coordinate (i.e., the *blue pattern* in Fig. 4 *f*). As such, signal photons that are collected after illumination with this pattern contain little information about the emitter *x*-position. The Two-pattern configuration of two-pattern configuration is thus able to distribute signal photons more efficiently to maximize the information about the emitter *x*-position.

On the other hand, as discussed earlier for Fig. S9, the two-pattern configuration contains little information about the emitter *y*-position. To investigate this for the triangle configuration, Fig. S11 shows the *x*-localization precision that can be achieved when the triangle pattern is rotated clockwise by 90° for all three scenarios under consideration. Equivalently, these results also hold for the *y*-precision that can be attained with the nonrotated pattern. It can be seen that the optimal spacing *r* and the localization precision are comparable with those for the nonrotated triangle configuration. We find a precision improvement in the *y*-direction of 2.05 over SMLM. As the rotated pattern is asymmetric along the *x*-axis, the precision also scales asymmetrically around the optimum. In addition, the asymmetry causes a shift to the optimal *x*-coordinate of the pattern focus. For example, the optimal focus position is $x_{f}=\theta_{x}-0.13$ nm when considering the scenario where the entire signal photon budget is exhausted. From the equivalence, we find that the triangle configuration balances the localization precision in the *x*- and *y*-directions at approximately a twofold improvement in either direction at the cost of suboptimal precision in each individual direction.

In MINFLUX (8,15), a triangle configuration was also used for illumination, where an additional fourth pattern was added in the center of the configuration. As such, we also consider the scenario where an additional pinhole and pattern are added in the center of the triangle for both rotations of the configuration (see Figs. S12 and S13).

From Figs. S12 and S13, we find that adding a center pinhole causes a deterioration of the localization precision compared with the triangle configuration without a center pinhole. The precision improvement over SMLM is at most 1.44 for the nonrotated pattern, and at most 1.78 for the rotated pattern. On the other hand, the domain where SpinFlux attains an improvement over SMLM has increased due to the addition of the center pinhole. For the nonrotated pattern with spacing $r=2\sigma_{\text{PSF}}$, the improvement over SMLM varies between 1.39 and 1.44 as long as the pattern focus and the emitter remain at a 130 nm distance from each other.

The explanation for both these effects is that the center pinhole blocks the least amount of signal photons, and also claims the majority of the signal photon budget due to illumination with near-maximum intensity. As such, as shown in Figs. S12, *f*, *g* and S13, *f*, *g*, the effect of the pinhole spacing *r* on the usage of the signal photon budget and background count is strongly reduced. For pattern spacings between $r=0.5\sigma_{\text{PSF}}$ and $r=2\sigma_{\text{PSF}}$, pattern focus positions within a 130 nm range of the emitter position and either rotation, signal photon counts vary between 1753 and 1968 photons, and average backgrounds vary between 0.88 and 4.30 photons per pixel. When the center of the triangle is displaced from the emitter position, another pinhole is able to cover the emitter position, thereby enlarging the range of similar photon counts and increasing the domain of precision improvement.

### Donut-shaped intensity patterns

Note that MINFLUX uses a donut-shaped intensity pattern for illumination, which contains an intensity minimum in the center. As described until now, SpinFlux uses a Gaussian intensity profile, with an intensity maximum in the center. By incorporating two phase masks in the system (see Fig. S3), SpinFlux can be adapted to utilize donut-shaped illumination. As the donut-shaped pattern increases the information content of signal photons in its center rather than at its boundary (8), it will mitigate the situation where highly informative signal photons are blocked by the pinhole, which in turn improves the theoretically minimum localization uncertainty. We explore this effect in Figs. 5 and S14–S17.

Figs. S14 and S15 show the SpinFlux localization precision of the triangular configuration without a center pinhole, in the scenario where the entire signal photon budget is exhausted. Here, the improvement of SpinFlux with donut-shaped illumination over SMLM is approximately 1.64 in the *x*-direction and 1.74 in the *y*-direction at a pinhole spacing $r=3\sigma_{\text{PSF}}$. This improvement is comparable with that of SpinFlux with Gaussian illumination, as the intensity minimum of the illumination donut is placed $3\sigma_{\text{PSF}}$ away from the emitter. The Gaussian pattern at $r=2\sigma_{\text{PSF}}$ and the donut-shaped pattern at $r=3\sigma_{\text{PSF}}$ are comparable on the emitter coordinates, thereby negating the advantages of the donut-shaped pattern.

This changes when including a center pinhole in the triangular configuration, as shown in Figs. 5, S16, and S17. Here, the maximum improvement over SMLM is 3.5 in the *x*- and *y*-directions at a pinhole spacing of $r=4\sigma_{\text{PSF}}$. When increasing the spacing *r* between the pinholes (beyond the width of the donut-shaped beam), a larger share of the signal photon budget will be claimed by the center pinhole. The intensity minimum of the center pinhole increases the information content of signal photons, thereby improving the resolution over SpinFlux with Gaussian illumination. However, this improvement decays sharply when the pattern focus is not centered on the emitter position. Specifically for $r=4\sigma_{\text{PSF}}$, the improvement exceeds 1.5 in either direction only when the emitter-focus distance is smaller than 5 nm. Therefore, it is more practical to choose a smaller spacing between the pinholes. For $r=3\sigma_{\text{PSF}}$, the maximum improvement over SMLM is 3.3 in the *x*- and *y*-directions, and the improvement is larger than 1.5 in either direction when the emitter-focus distance is at most 37 nm.

## Discussion

In meSMLM, sparse activation of single emitters with patterned illumination results in improved localization precision over SMLM. The precision improvement of meSMLM is derived from retrieving the position of an emitter relative to individual illumination patterns, which adds to existing PSF information from SMLM. In addition, meSMLM improves the resolution over image reconstruction in SIM while reducing the required amount of illumination patterns. This suggests that meSMLM can improve the localization precision in existing setups, which are limited by image reconstruction in processing.

We developed SpinFlux, which incorporates meSMLM into SDCM setups. In SpinFlux, patterned illumination is generated using a spinning disk with pinholes to sequentially illuminate the sample. Subsequently, the emission signal is windowed by the same pinhole before being imaged on the camera. During the analysis, emitters are localized in the recordings from a sequence of individual pattern acquisitions, taking knowledge about the pattern into account.

We have derived a statistical image formation model for SpinFlux, which includes the effects of patterned illumination, windowing of the emission signal by the pinhole and pattern-dependent background. For our analysis, we considered Gaussian illumination patterns and a Gaussian emission PSF. We also consider donut-shaped illumination patterns, which can be generated by incorporating a phase mask in the illumination path. In addition, we have derived and evaluated the CRLB for this model. We applied the CRLB to various illumination pattern configurations to quantify the theoretical minimum uncertainty that can be gained with SpinFlux. We compared SpinFlux with SMLM and with localization on ISM reconstruction data, which results in an average global improvement of 1.48 over SMLM, or 2.10 with Fourier reweighting.

When using one pattern only, pattern dependency of the background causes an improvement of at most 1.17 over SMLM, whereas no improvement is found when neglecting this effect. In the single-pattern case, the pinhole blocks signal photons that carry information about the emitter position. As such, it is beneficial for SpinFlux to use pinholes that are as large as possible to reduce the amount of signal photons blocked by the pinhole. In other words, we find that a spinning disk with pinholes is convenient to generate patterned illumination, although the pinhole itself has an adverse effect on the localization precision due to the blockage of signal photons. However, we have not considered neighboring emitters in our analysis, nor have we modeled out-of-focus background. In ISM, optical sectioning is achieved with the spinning disk by reducing the effects of neighboring or out-of-focus fluorescent signals, thereby improving the resolution. We expect that the pinhole has a similar effect on the localization precision that can be attained with SpinFlux, thereby resulting in an optimal pinhole radius. Future research should focus on incorporating these effects into the image formation model.

Based on the single-pattern results, we conclude that SpinFlux requires multiple patterns to generate a significant precision improvement over SMLM. We explored various multiple-pattern configurations, which can be obtained via sequential illumination. We found that a configuration of two pinholes with radius $3\sigma_{\text{PSF}}$, separated in the *x*-direction around the emitter position by a distance of $4\sigma_{\text{PSF}}$, results in a precision improvement of 2.62 in the *x*-direction compared with SMLM, while the *y*-improvement is at most 1.12. For larger separations, the information content of signal photons increases due to illumination with the low-intensity tails of the Gaussian illumination pattern. However, when the separation increases above $4\sigma_{\text{PSF}}$, the loss of signal photons due to the windowing effect of the pinhole causes deterioration of the localization precision.

We also evaluated the theoretical minimum uncertainty of a triangular pattern configuration, where pinholes are sequentially placed at the corners of an equilateral triangle around the emitter position. This results in approximately a twofold *x*-precision improvement over SMLM, which is a reduction compared with the two-pattern configuration. However, the triangle configuration also attains approximately a twofold precision improvement in the *y*-direction. As such, the triangle configuration balances the localization precision in the *x*- and *y*-directions at the cost of suboptimal precision in each individual direction. Including a center pinhole in the triangle does not improve the maximum localization improvement, but it extends the domain on which any improvement can be attained.

By including a phase mask in the illumination and emission paths, illumination patterns with arbitrary diffraction-limited intensity profiles can be created. We evaluated the localization precision of SpinFlux with donut-shaped illumination. As the donut-shaped pattern increases the information content of signal photons in its center rather than at its boundary, it will mitigate the situation where highly informative signal photons are blocked by the pinhole. We find that, in the triangular configuration with a center pinhole, the maximum improvement over SMLM is increased to 3.5 in the *x*- and *y*-directions at a pinhole spacing $r=4\sigma_{\text{PSF}}$.

We conclude that localization on ISM reconstruction data results is the most straightforward implementation and results in the largest global average improvement of the localization precision. On the other hand, SpinFlux is the method of choice for local refinements of the localization precision. In addition, the versatility of the image formation model makes SpinFlux analysis on non-Gaussian illumination patterns straightforward.

## Data and code availability

The data that support the findings of this study are openly available in 4TU.ResearchData (31) at https://doi.org/10.4121/21313230. The code that supports the findings of this study is openly available on GitHub (32) at https://github.com/qnano/spinflux-crlb.

## Author contributions

D.K., S.H., and C.S.S. designed the research. D.K. derived and implemented the model, analyzed the data, and wrote the manuscript, which was edited by S.H. and C.S.S. The study was supervised by C.S.S.

## Supporting citations

The following references appear in the supporting material: (33,34,35,36,37,38).

## Supporting material

Document S1. Figures S1–S45, Table S1, and Notes S1–S4

Document S2. Article plus supporting material
