# Supplementary material for: Quantifying the minimum localization uncertainty of image scanning localization microscopy
Source: Biophys Rep (N Y). 2024 Jan 20;4(1):100143. doi: 10.1016/j.bpr.2024.100143 (PMC10878846; doi:10.1016/j.bpr.2024.100143)
Supplement: Document S1. Figures S1–S45, Table S1, and Notes S1–S4 [file mmc1.pdf]

Biophysical Reports, Volume *4*

Supplemental information

Quantifying the minimum localization uncertainty of image scanning localization microscopy

Dylan Kalisvaart, Shih-Te Hung, and Carlas S. Smith

TABLE OF CONTENTS:

**Note S1**	Theoretical approximation of the best-case localization precision of localization on image scanning microscopy data.
**Note S2**	Image formation model for SpinFlux localization.
**Note S3**	Cramér-Rao lower bound for SpinFlux localization.
**Note S4**	Derivatives of the SpinFlux image formation model, needed to compute the Cramér-Rao lower bound.
**Figure** S1	Approximation of the theoretical minimum localization uncertainty of single-molecule localization microscopy on data acquired from spinning disk confocal microscopy.
**Figure** S2	Relative error in the x-Cramér-Rao lower bound resulting from the discretized pinhole approximation.
**Figure** S3	Schematic overview of SpinFlux image formation with donut-shaped illumination patterns.
**Figure** S4	Theoretical minimum localization uncertainty of SpinFlux localization with one x-offset pinhole and pattern, for the scenario where the entire signal photon budget is exhausted after illumination with the pattern.
**Figure** S5	Theoretical minimum localization uncertainty of SpinFlux localization with one y-offset pinhole and pattern, for the scenario where the entire signal photon budget is exhausted after illumination with the pattern.
**Figure** S6	Theoretical minimum localization uncertainty of SpinFlux localization with two pinholes and patterns separated in the x-direction, for the scenario where the entire signal photon budget is exhausted after illumination with all patterns.
**Figure** S7	Theoretical minimum localization uncertainty of SpinFlux localization with two y-offset pinholes and patterns separated in the x-direction, for the scenario where the entire signal photon budget is exhausted after illumination with all patterns.
**Figure** S8	Theoretical minimum localization uncertainty of SpinFlux localization with two patterns without pinholes separated in the x-direction, for the scenario where the entire signal photon budget is exhausted after illumination with all patterns.
**Figure** S9	Theoretical minimum localization uncertainty of SpinFlux localization with two pinholes and patterns separated in the y-direction, for the scenario where the entire signal photon budget is exhausted after illumination with all patterns.
**Figure** S10	Theoretical minimum localization uncertainty of SpinFlux localization with three pinholes and patterns in an equilateral triangle configuration, for the scenario where the entire signal photon budget is exhausted after illumination with all patterns.
**Figure** S11	Theoretical minimum localization uncertainty of SpinFlux localization with three pinholes and patterns in a 90° rotated equilateral triangle configuration, for the scenario where the entire signal photon budget is exhausted after illumination with all patterns.
**Figure** S12	Theoretical minimum localization uncertainty of SpinFlux localization with four pinholes and patterns in an equilateral triangle configuration with a center pinhole, for the scenario where the entire signal photon budget is exhausted after illumination with all patterns.
**Figure** S13	Theoretical minimum localization uncertainty of SpinFlux localization with four pinholes and patterns in a 90° rotated equilateral triangle configuration with a center pinhole, for the scenario where the entire signal photon budget is exhausted after illumination with all patterns.
**Figure** S14	Theoretical minimum localization uncertainty of SpinFlux localization with three pinholes and donut-shaped patterns in an equilateral triangle configuration, for the scenario where the entire signal photon budget is exhausted after illumination with all patterns.
**Figure** S15	Theoretical minimum localization uncertainty of SpinFlux localization with three pinholes and donut-shaped patterns in a 90° rotated equilateral triangle configuration, for the scenario where the entire signal photon budget is exhausted after illumination with all patterns.

**Figure** S16	Theoretical minimum localization uncertainty of SpinFlux localization with four pinholes and donut-shaped patterns in an equilateral triangle configuration with a center pinhole, for the scenario where the entire signal photon budget is exhausted after illumination with all patterns.
**Figure** S17	Theoretical minimum localization uncertainty of SpinFlux localization with four pinholes and donut-shaped patterns in a 90° rotated equilateral triangle configuration with a center pinhole, for the scenario where the entire signal photon budget is exhausted after illumination with all patterns.
**Figure** S18	Theoretical minimum localization uncertainty of SpinFlux localization with one x-offset pinhole and pattern, for the scenario where the illumination power and time are constant during illumination with this pattern.
**Figure** S19	Theoretical minimum localization uncertainty of SpinFlux localization with one y-offset pinhole and pattern, for the scenario where the illumination power and time are constant during illumination with this pattern.
**Figure** S20	Theoretical minimum localization uncertainty of SpinFlux localization with two pinholes and patterns separated in the x-direction, for the scenario where the illumination power and time are constant during illumination with all patterns.
**Figure** S21	Theoretical minimum localization uncertainty of SpinFlux localization with two y-offset pinholes and patterns separated in the x-direction, for the scenario where the illumination power and time are constant during illumination with all patterns.
**Figure** S22	Theoretical minimum localization uncertainty of SpinFlux localization with two patterns without pinholes separated in the x-direction, for the scenario where the illumination power and time are constant during illumination with all patterns.
**Figure** S23	Theoretical minimum localization uncertainty of SpinFlux localization with two pinholes and patterns separated in the y-direction, for the scenario where the illumination power and time are constant during illumination with all patterns.
**Figure** S24	Theoretical minimum localization uncertainty of SpinFlux localization with three pinholes and patterns in an equilateral triangle configuration, for the scenario where the illumination power and time are constant during illumination with all patterns.
**Figure** S25	Theoretical minimum localization uncertainty of SpinFlux localization with three pinholes and patterns in a 90° rotated equilateral triangle configuration, for the scenario where the illumination power and time are constant during illumination with all patterns.
**Figure** S26	Theoretical minimum localization uncertainty of SpinFlux localization with four pinholes and patterns in an equilateral triangle configuration with a center pinhole, for the scenario where the illumination power and time are constant during illumination with all patterns.
**Figure** S27	Theoretical minimum localization uncertainty of SpinFlux localization with four pinholes and patterns in a 90° rotated equilateral triangle configuration with a center pinhole, for the scenario where the illumination power and time are constant during illumination with all patterns.
**Figure** S28	Theoretical minimum localization uncertainty of SpinFlux localization with three pinholes and donut-shaped patterns in an equilateral triangle configuration, for the scenario where the illumination power and time are constant during illumination with all patterns.
**Figure** S29	Theoretical minimum localization uncertainty of SpinFlux localization with three pinholes and donut-shaped patterns in a 90° rotated equilateral triangle configuration, for the scenario where the illumination power and time are constant during illumination with all patterns.
**Figure** S30	Theoretical minimum localization uncertainty of SpinFlux localization with four pinholes and donut-shaped patterns in an equilateral triangle configuration with a center pinhole, for the scenario where the illumination power and time are constant during illumination with all patterns.

**Figure** S31	Theoretical minimum localization uncertainty of SpinFlux localization with four pinholes and donut-shaped patterns in a 90° rotated equilateral triangle configuration with a center pinhole, for the scenario where the illumination power and time are constant during illumination with all patterns.
**Figure** S32	Theoretical minimum localization uncertainty of SpinFlux localization with one x-offset pinhole and pattern, for the scenario where the entire signal photon budget is exhausted after illumination with the pattern, neglecting the effects of pattern-dependent background.
**Figure** S33	Theoretical minimum localization uncertainty of SpinFlux localization with one y-offset pinhole and pattern, for the scenario where the entire signal photon budget is exhausted after illumination with the pattern, neglecting the effects of pattern-dependent background.
**Figure** S34	Theoretical minimum localization uncertainty of SpinFlux localization with two pinholes and patterns separated in the x-direction, for the scenario where the entire signal photon budget is exhausted after illumination with all patterns, neglecting the effects of pattern-dependent background.
**Figure** S35	Theoretical minimum localization uncertainty of SpinFlux localization with two y-offset pinholes and patterns separated in the x-direction, for the scenario where the entire signal photon budget is exhausted after illumination with all patterns, neglecting the effects of pattern-dependent background.
**Figure** S36	Theoretical minimum localization uncertainty of SpinFlux localization with two patterns without pinholes separated in the x-direction, for the scenario where the entire signal photon budget is exhausted after illumination with all patterns, neglecting the effects of pattern-dependent background.
**Figure** S37	Theoretical minimum localization uncertainty of SpinFlux localization with two pinholes and patterns separated in the y-direction, for the scenario where the entire signal photon budget is exhausted after illumination with all patterns, neglecting the effects of pattern-dependent background.
**Figure** S38	Theoretical minimum localization uncertainty of SpinFlux localization with three pinholes and patterns in an equilateral triangle configuration, for the scenario where the entire signal photon budget is exhausted after illumination with all patterns, neglecting the effects of pattern-dependent background.
**Figure** S39	Theoretical minimum localization uncertainty of SpinFlux localization with three pinholes and patterns in a 90° rotated equilateral triangle configuration, for the scenario where the entire signal photon budget is exhausted after illumination with all patterns, neglecting the effects of pattern-dependent background.
**Figure** S40	Theoretical minimum localization uncertainty of SpinFlux localization with four pinholes and patterns in an equilateral triangle configuration with a center pinhole, for the scenario where the entire signal photon budget is exhausted after illumination with all patterns, neglecting the effects of pattern-dependent background.
**Figure** S41	Theoretical minimum localization uncertainty of SpinFlux localization with four pinholes and patterns in a 90° rotated equilateral triangle configuration with a center pinhole, for the scenario where the entire signal photon budget is exhausted after illumination with all patterns, neglecting the effects of pattern-dependent background.
**Figure** S42	Theoretical minimum localization uncertainty of SpinFlux localization with three pinholes and donut-shaped patterns in an equilateral triangle configuration, for the scenario where the entire signal photon budget is exhausted after illumination with all patterns, neglecting the effects of pattern-dependent background.
**Figure** S43	Theoretical minimum localization uncertainty of SpinFlux localization with three pinholes and donut-shaped patterns in a 90° rotated equilateral triangle configuration, for the scenario where the entire signal photon budget is exhausted after illumination with all patterns, neglecting the effects of pattern-dependent background.
**Figure** S44	Theoretical minimum localization uncertainty of SpinFlux localization with four pinholes and donut-shaped patterns in an equilateral triangle configuration with a center pinhole, for the scenario where the entire signal photon budget is exhausted after illumination with all patterns, neglecting the effects of pattern-dependent background.

**Figure S45**	Theoretical minimum localization uncertainty of SpinFlux localization with four pinholes and donut-shaped patterns in a 90° rotated equilateral triangle configuration with a center pinhole, for the scenario where the entire signal photon budget is exhausted after illumination with all patterns, neglecting the effects of pattern-dependent background.
**Table S1**	Model parameters used in SpinFlux simulations.

NOTE S1: THEORETICAL APPROXIMATION OF THE BEST-CASE LOCALIZATION PRECISION OF LOCALIZATION ON IMAGE SCANNING MICROSCOPY DATA

In this note, we derive a theoretical approximation of the best-case localization precision that can be achieved by localizing emitters on ISM reconstructions. Concretely, we assume that enough illumination patterns are used to uniformly illuminate the sample. For ideal ISM reconstructions (1, 2), the effective PSF standard deviation after reconstruction is reduced by a factor $\sqrt{2}$. If the ISM reconstructions are subsequently Fourier reweighted, the effective PSF standard deviation is reduced further, up to a total factor 2.

From (3, 4), we find that the theoretical minimum localization uncertainty σ_x of SMLM can be approximated as:

$$\sigma_x^2 = \frac{\sigma_{\text{PSF}}^2 + \Delta x^2/12}{\theta_I}\left(1 + 4\tau + \sqrt{\frac{2\tau}{1+4\tau}}\right). \tag{S1}$$

Here, σ_{PSF} denotes the standard deviation of the Gaussian point spread function (PSF), Δx is the pixel size and θ_I is the expected signal photon budget. In addition, τ is a normalized dimensionless background parameter

$$\tau = \frac{2\pi\theta_b(\sigma_{\text{PSF}}^2 + \Delta x^2/12)}{\theta_I \Delta x^2}, \tag{S2}$$

where θ_b denotes the expected amount of background photons per pixel.

If individual emitters are localized in ISM data, the standard deviation of the best-case ISM PSF is given by

$$\sigma_{\text{PSF, ISM}} = \frac{\sigma_{\text{PSF}}}{\sqrt{2}}. \tag{S3}$$

The approximation of the theoretically minimum localization precision is then given by:

$$\sigma_{x,\text{ ISM}}^2 = \frac{\sigma_{\text{PSF}}^2/2 + \Delta x^2/12}{\theta_I}\left(1 + 4\tau_{\text{ISM}} + \sqrt{\frac{2\tau_{\text{ISM}}}{1+4\tau_{\text{ISM}}}}\right), \tag{S4}$$

$$\tau_{\text{ISM}} = \frac{2\pi\theta_b(\sigma_{\text{PSF}}^2/2 + \Delta x^2/12)}{\theta_I \Delta x^2}. \tag{S5}$$

For ISM reconstructions with Fourier reweighting, the analysis is identical with a reduction of σ_{PSF} by a factor two.

As described in Equations S4 and S5, the localization precision depends on a combination of the PSF size and the pixel size. If the PSF size is small compared to the pixel size, the localization precision becomes proportional to the pixel size. On the other hand, if the PSF size is large compared to the pixel size, the localization precision becomes a (non-proportional) function of the PSF size. In that case, the ratio between the PSF size and the pixel size influences the weighting of the signal-to-background ratio in determining the localization precision, through the parameter τ_{ISM} in Equation S5. This dependency is shown in Figure S1.

NOTE S2: IMAGE FORMATION MODEL FOR SPINFLUX LOCALIZATION

In this note, we derive a statistical image formation model for SpinFlux modulation enhanced single molecule localization microscopy. We start by formulating a model for one pinhole and one illumination pattern, then we extend the model for arbitrary amounts of pinholes and patterns.

In Note S2, we will model the amount of photons that are acquired by a camera pixel through the Poisson distribution. As such, we aim to find a model for the Poisson mean μ_i here, to describe the expected amount of photons recorded on each camera pixel i. We follow a similar modeling procedure as (5–8) to derive a model for the Poisson mean μ_i.

An image $\tilde{g}(x, y)$ of an object $f(x, y)$ is formed through an optical system with point spread function (PSF) $h(x, y)$ through a convolution, as shown in Equation (S6).

$$\tilde{g}(x, y) = h(x, y) \otimes f(x, y). \tag{S6}$$

In Equation (S6), $\otimes$ denotes the two-dimensional convolution operator. In this equation, we need to ensure that the total area under the PSF equals 1, to avoid that the optical system adds energy to the image formation process. This results in the normalization condition of Equation (S7).

$$\iint_{\mathbb{R}^2} h(x, y)\mathrm{d}x\mathrm{d}y = 1. \tag{S7}$$

We will now propose a model for the object function $f(x)$ when a single pinhole and illumination pattern are used. Consider a point emitter, located at a position (θ_x, θ_y). Such an emitter can be modeled as $\delta(x - \theta_x, y - \theta_y)$, where δ denotes the two-dimensional delta function. Under non-uniform illumination with a pattern $P(x - x_\mathrm{p}, y - y_\mathrm{p})$ centered at pinhole center coordinates $(x_\mathrm{p}, y_\mathrm{p})$, the expected amount of signal photons emitted by this emitter is $P(x - x_\mathrm{p}, y - y_\mathrm{p})\theta_I$ and the expected pattern-dependent background count is $P(x - x_\mathrm{p}, y - y_\mathrm{p})\theta_b$. Here, the (dimensionless) illumination intensity needs to satisfy $0 \leq P(x - x_\mathrm{p}, y - y_\mathrm{p}) \leq 1$ for all $(x, y) \in \mathbb{R}^2$, and $P(x - x_\mathrm{p}, y - y_\mathrm{p}) = 1$ for some $(x, y) \in \mathbb{R}^2$ to ensure there exists a coordinate which receives maximum illumination. The resulting object function $f(x, x_\mathrm{p}, y, y_\mathrm{p})$ is shown in Equation (S8).

$$f(x, x_\mathrm{p}, y, y_\mathrm{p}) = P(x - x_\mathrm{p}, y - y_\mathrm{p})(\theta_I \delta(x - \theta_x, y - \theta_y) + \theta_b). \tag{S8}$$

To obtain an expression for the image function $\tilde{g}(x, y)$, we evaluate the convolution in Equation (S6). This ultimately results in the expression for $\tilde{g}(x, y)$ as shown in Equation (S9).

$$\tilde{g}(x, x_\mathrm{p}, y, y_\mathrm{p}) = \theta_I P(\theta_x - x_\mathrm{p}, \theta_y - y_\mathrm{p}) h(x - \theta_x, y - \theta_y) + \theta_b \iint_{\mathbb{R}^2} h(\tau, \gamma) P(x - x_\mathrm{p} - \tau, y - y_\mathrm{p} - \gamma)\mathrm{d}\tau\mathrm{d}\gamma. \tag{S9}$$

For SpinFlux, the image $\tilde{g}(x, y)$ is not imaged on the camera directly. Instead, $\tilde{g}(x, x_\mathrm{p}, y, y_\mathrm{p})$ passes through a circular pinhole, resulting in the circularly windowed image $g(x, y)$. Let r_p describe the radius of the pinhole. For notation convenience, we define the pinhole area $S_\mathrm{p} = \left\{(x, y) | (x - x_\mathrm{p})^2 + (y - y_\mathrm{p})^2 \leq r_\mathrm{p}^2\right\}$. We describe the pinhole with center coordinates $(x_\mathrm{p}, y_\mathrm{p})$ by the pinhole mask $\Pi(x, x_\mathrm{p}, y, y_\mathrm{p})$.

$$\Pi(x, x_\mathrm{p}, y, y_\mathrm{p}) = \begin{cases} 1, & \text{if } (x, y) \in S_\mathrm{p}, \\ 0, & \text{otherwise.} \end{cases} \tag{S10}$$

We identified two different ways of modelling a confocal pinhole in literature. In the first type of models (1, 9, 10), the pinhole is included as a product with emission point-spread function. A second class of models exists, where the confocal aperture is modelled as a convolution with the emission point-spread function (11, 12).

From (9), we infer that that the product model is valid in case the pupil stop of the objective lens is much smaller than the pupil stop of the pinhole. We therefore choose this model and limit ourselves to simulation conditions where this is the case. This means our image formation model is not suited for the case where the pinhole stop is more limiting than the objective lens.

The image $g(x, y)$ on the camera after windowing by the pinhole is now given by:

$$g(x, y) = \Pi(x, x_\mathrm{p}, y, y_\mathrm{p})\tilde{g}(x, y). \tag{S11}$$

As a next step, we need to discretize the image function $g(x, y)$ on the camera pixel array. Let $S_{\mathrm{c},i}$ denote the area belonging to the camera pixel with index i. To discretize the image function on camera pixel i, we integrate it over all $(x, y) \in S_{\mathrm{c},i}$ to obtain the expected amount of photons on the i'th pixel, μ_i. Let (x_i, y_i) denote the center coordinates of the i'th pixel. We then find:

$$\mu_i(x_i, x_\mathrm{p}, y_i, y_\mathrm{p}) = \theta_I P(\theta_x - x_\mathrm{p}, \theta_y - y_\mathrm{p}) \iint_{(x,y)\in S_{\mathrm{c},i}} \Pi(x, x_\mathrm{p}, y, y_\mathrm{p}) h(x-\theta_x, y-\theta_y)\mathrm{d}x\mathrm{d}y \\ + \theta_b \iint_{(x,y)\in S_{\mathrm{c},i}} \Pi(x, x_\mathrm{p}, y, y_\mathrm{p}) \left(\iint_{\mathbb{R}^2} h(\tau,\gamma) P(x - x_\mathrm{p} - \tau, y - y_\mathrm{p} - \gamma)\mathrm{d}\tau\mathrm{d}\gamma\right)\mathrm{d}x\mathrm{d}y. \tag{S12}$$

The aperture mask $\Pi(x, x_\mathrm{p}, y, y_\mathrm{p})$ from Equation (S10) acts as a window on the integrands, thereby constraining the relevant domain of integration to the overlapping area between the camera pixel area $S_{\mathrm{c},i}$ and the pinhole area S_p. We denote this overlapping area as $\left(S_{\mathrm{c},i} \cap S_\mathrm{p}\right)$. Equation (S12) then becomes Equation (S13).

$$\mu_i(x_i, x_\mathrm{p}, y_i, y_\mathrm{p}) = \theta_I P(\theta_x - x_\mathrm{p}, \theta_y - y_\mathrm{p}) \iint_{(x,y)\in\left(S_{\mathrm{c},i}\cap S_\mathrm{p}\right)} h(x-\theta_x, y-\theta_y)\mathrm{d}x\mathrm{d}y \\ + \theta_b \underbrace{\iint_{(x,y)\in\left(S_{\mathrm{c},i}\cap S_\mathrm{p}\right)} \left(\iint_{\mathbb{R}^2} h(\tau,\gamma) P(x - x_\mathrm{p} - \tau, y - y_\mathrm{p} - \gamma)\mathrm{d}\tau\mathrm{d}\gamma\right)\mathrm{d}x\mathrm{d}y}_{B_i}. \tag{S13}$$

Note that the effective background B_i is a constant, which does not depend on the emitter position, but only on the camera pixel area, the pinhole area, the PSF and the illumination pattern. We can thus give a compact expression for the Poisson mean μ_i, as shown in Equation (S14).

$$\mu_i(x_i, x_\mathrm{p}, y_i, y_\mathrm{p}) = \theta_I P(\theta_x - x_\mathrm{p}, \theta_y - y_\mathrm{p}) \iint_{(x,y)\in\left(S_{\mathrm{c},i}\cap S_\mathrm{p}\right)} h(x-\theta_x, y-\theta_y)\mathrm{d}x\mathrm{d}y + \theta_b B_i. \tag{S14}$$

Approximation of domain of integration

To evaluate the integrations in Equation (S14), we need to describe the domain of integration $\left(S_{\mathrm{c},i} \cap S_\mathrm{p}\right)$. That is, we have to find the overlapping area of the camera pixel with center coordinates (x_i, y_i) and the pinhole with center coordinates $(x_\mathrm{p}, y_\mathrm{p})$. To this extent, let us assume a camera for which all pixels have the same shape and size. Let all pixels be rectangular, with length Δx in the x-direction and length Δy in the y-direction. Furthermore, let N_x, N_y be the amount of camera pixels in each direction (with the total amount of pixels being $N_\mathrm{pixels} = N_x N_y$).

To simplify our analysis, we resort to a numerical approximation of the intersection, where we approximate the pinhole S_p and the overlapping area $\left(S_{\mathrm{c},i} \cap S_\mathrm{p}\right)$ on a square mesh. Let $(x_{\mathrm{M},j}, y_{\mathrm{M},j})$ describe the center coordinates of mesh pixel j, defined in the same coordinate system as the pixel coordinates (x_i, y_i). We define the mesh to have $N_{\mathrm{M},x}, N_{\mathrm{M},y}$ pixels in each direction (with the total amount of mesh pixels being $N_\mathrm{M} = N_{\mathrm{M},x} N_{\mathrm{M},y}$). We parametrize the mesh width as $\Delta x_\mathrm{M} = \frac{N_x}{N_{\mathrm{M},x}} \cdot \Delta x$ in the x-direction and as $\Delta y_\mathrm{M} = \frac{N_y}{N_{\mathrm{M},y}} \cdot \Delta y$ in the y-direction. To avoid cases where mesh pixels partially overlap with camera pixels, we restrict choices of $N_{\mathrm{M},x}$ and $N_{\mathrm{M},y}$ to integer multiples of N_x and N_y. Note that increasing the amount of mesh pixels $N_{\mathrm{M},x}$ and $N_{\mathrm{M},y}$ decreases the mesh widths Δx_M and Δy_M and thus improves the accuracy of the numerical approximation.

On the mesh, we approximate the pinhole area S_p as $\tilde{S}_\mathrm{p}$. We propose the following midpoint approximation, which assigns the area of mesh pixel j to the approximated pinhole area $\tilde{S}_\mathrm{p}$ if its center coordinate $(x_{\mathrm{M},j}, y_{\mathrm{M},j})$ falls within S_p:

$$\left[x_{\mathrm{M},j} - \frac{\Delta x_\mathrm{M}}{2}, x_{\mathrm{M},j} + \frac{\Delta x_\mathrm{M}}{2}\right] \times \left[y_{\mathrm{M},j} - \frac{\Delta y_\mathrm{M}}{2}, y_{\mathrm{M},j} + \frac{\Delta y_\mathrm{M}}{2}\right] \subseteq \tilde{S}_\mathrm{p} \text{ if } \left(x_{\mathrm{M},j}, y_{\mathrm{M},j}\right) \in S_\mathrm{p}. \tag{S15}$$

As $\tilde{S}_\mathrm{p}$ is a square mesh in the same coordinate system as the camera pixel area $S_{\mathrm{c},i}$, the intersection $\left(S_{\mathrm{c},i} \cap \tilde{S}_\mathrm{p}\right)$ is straightforward to evaluate. Namely, this intersection consists of those mesh pixels in $\tilde{S}_\mathrm{p}$ that are also contained in $S_{\mathrm{c},i}$. Formally, this is the set of all mesh pixels j for which the center coordinates satisfy $(x_{\mathrm{M},j}, y_{\mathrm{M},j}) \in \left(S_{\mathrm{c},i} \cap \tilde{S}_\mathrm{p}\right)$. This is illustrated in Figure 1c of the main text.

Using this numerical approximation, Equation (S14) can be rewritten as follows:

$$\mu_i(x_i, x_\mathrm{p}, y_i, y_\mathrm{p}) = \theta_I P(\theta_x - x_\mathrm{p}, \theta_y - y_\mathrm{p}) \\ \cdot \sum_{(x_{\mathrm{M},j}, y_{\mathrm{M},j})\in\left(S_{\mathrm{c},i}\cap\tilde{S}_\mathrm{p}\right)} \int_{x_{\mathrm{M},j}-\frac{\Delta x_\mathrm{M}}{2}}^{x_{\mathrm{M},j}+\frac{\Delta x_\mathrm{M}}{2}} \int_{y_{\mathrm{M},j}-\frac{\Delta y_\mathrm{M}}{2}}^{y_{\mathrm{M},j}+\frac{\Delta y_\mathrm{M}}{2}} h(x-\theta_x, y-\theta_y)\mathrm{d}x\mathrm{d}y + \theta_b B_i. \tag{S16}$$

Illumination and emission point spread functions

As a model for the illumination PSF $P(x - x_p, y - y_p)$ and the emission PSF $h(x, y)$, we choose to use Gaussians. The illumination PSF is given by Equation (S17), where σ_{illum} denotes the standard deviation of the illumination PSF.

$$P_{\text{Gaussian}}(x - x_p, y - y_p) = e^{\frac{-(x-x_p)^2-(y-y_p)^2}{2\sigma_{\text{illum}}^2}}. \tag{S17}$$

Alternate illumination patterns can be generated by placing a phase mask in the illumination path. We therefore also include a model of the donut-shaped pattern from e.g. MINFLUX (13), with a zero-intensity minimum at the center of the pinhole and standard deviation σ_{illum}:

$$P_{\text{donut}}(x - x_p, y - y_p) = e \cdot \left(\frac{(x - x_p)^2 + (y - y_p)^2}{2\sigma_{\text{illum}}^2} \right) e^{\frac{-(x-x_p)^2-(y-y_p)^2}{2\sigma_{\text{illum}}^2}}. \tag{S18}$$

Note that for the Gaussian and donut illumination models, the condition $0 \le P(x - x_p, y - y_p) \le 1$ is satisfied, with $P_{\text{Gaussian}}(x - x_p, y - y_p) = 1$ for $x = x_p$, $y = y_p$ and with $P_{\text{donut}}(x - x_p, y - y_p) = 1$ for $(x - x_p)^2 + (y - y_p)^2 = 2\sigma_{\text{illum}}^2$.

The emission PSF is given by Equation (S19), where σ_{PSF} denotes the standard deviation of the emission PSF.

$$h(x, y) = \frac{1}{2\pi\sigma_{\text{PSF}}^2} e^{\frac{-x^2-y^2}{2\sigma_{\text{PSF}}^2}}. \tag{S19}$$

Note that for the Gaussian emission PSF model, the condition of Equation (S7) is satisfied. Furthermore, note that the exponential term in Equation (S19) can be split up as a product of two exponentials, of which one is dependent on x and of which the other is dependent on y. Using this property, we can further simplify the expression for the Poisson mean μ_i:

$$\begin{aligned}\mu_i(x_i, x_p, y_i, y_p) &= \theta_I P(\theta_x - x_p, \theta_y - y_p) \\ &\cdot \sum_{(x_{M,j}, y_{M,j}) \in (S_{c,i} \cap \tilde{S}_p)} \underbrace{\left(\int_{x_{M,j} - \frac{\Delta x_M}{2}}^{x_{M,j} + \frac{\Delta x_M}{2}} \frac{1}{\sigma_{\text{PSF}}\sqrt{2\pi}} e^{-\frac{(x-\theta_x)^2}{2\sigma_{\text{PSF}}^2}} \mathrm{d}x \right)}_{E(x_{M,j}-\theta_x, \Delta x_M, \sigma_{\text{PSF}}^2)} \underbrace{\left(\int_{y_{M,j} - \frac{\Delta y_M}{2}}^{y_{M,j} + \frac{\Delta y_M}{2}} \frac{1}{\sigma_{\text{PSF}}\sqrt{2\pi}} e^{-\frac{(y-\theta_y)^2}{2\sigma_{\text{PSF}}^2}} \mathrm{d}y \right)}_{E(y_{M,j}-\theta_y, \Delta y_M, \sigma_{\text{PSF}}^2)} \\ &+ \theta_b B_i.\end{aligned} \tag{S20}$$

We will use the error function to evaluate the integrations from Equation (S20). To this extent, we introduce the function $E(x, \Delta x, \sigma^2)$:

$$E(x, \Delta x, \sigma^2) = \frac{1}{2}\text{erf}\left(\frac{x + \frac{\Delta x}{2}}{\sqrt{2}\sigma} \right) - \frac{1}{2}\text{erf}\left(\frac{x - \frac{\Delta x}{2}}{\sqrt{2}\sigma} \right). \tag{S21}$$

The integrations then evaluate to:

$$E(x_{M,j} - \theta_x, \Delta x_M, \sigma_{\text{PSF}}^2) = \frac{1}{2}\text{erf}\left(\frac{x_{M,j} - \theta_x + \frac{\Delta x_M}{2}}{\sqrt{2}\sigma_{\text{PSF}}} \right) - \frac{1}{2}\text{erf}\left(\frac{x_{M,j} - \theta_x - \frac{\Delta x_M}{2}}{\sqrt{2}\sigma_{\text{PSF}}} \right), \tag{S22}$$

$$E(y_{M,j} - \theta_y, \Delta y_M, \sigma_{\text{PSF}}^2) = \frac{1}{2}\text{erf}\left(\frac{y_{M,j} - \theta_y + \frac{\Delta y_M}{2}}{\sqrt{2}\sigma_{\text{PSF}}} \right) - \frac{1}{2}\text{erf}\left(\frac{y_{M,j} - \theta_y - \frac{\Delta y_M}{2}}{\sqrt{2}\sigma_{\text{PSF}}} \right). \tag{S23}$$

In final, we obtain the Poisson mean μ_i as shown in Equation (S24).

$$\mu_i(x_i, x_p, y_i, y_p) = \theta_I P(\theta_x - x_p, \theta_y - y_p) \sum_{(x_{M,j}, y_{M,j}) \in (S_{c,i} \cap \tilde{S}_p)} E(x_{M,j} - \theta_x, \Delta x_M, \sigma_{\text{PSF}}^2) E(y_{M,j} - \theta_y, \Delta y_M, \sigma_{\text{PSF}}^2) + \theta_b B_i. \tag{S24}$$

For convenience, we collect the terms $\sum_{(x_{M,j}, y_{M,j}) \in (S_{c,i} \cap \tilde{S}_p)} E(x_{M,j} - \theta_x, \Delta x_M, \sigma_{\text{PSF}}^2) E(y_{M,j} - \theta_y, \Delta y_M, \sigma_{\text{PSF}}^2)$ in the discretized emission PSF term $H(\theta_x, \theta_y, x_i, y_i)$. By doing so, we retrieve the image formation model as in the main text:

$$\mu_i(x_i, x_p, y_i, y_p) = \theta_I P(\theta_x - x_p, \theta_y - y_p) H(\theta_x, \theta_y, x_i, y_i) + \theta_b B_i. \tag{S25}$$

Effective background B_i

In Equation (S13), a constant term B_i was identified which describes the effective background, given the camera pixel area, the pinhole area, the PSF and the illumination pattern. Using the discretized approximation of the pinhole area, $B_{i,\text{Gaussian}}$ can be expressed as follows:

$$B_i = \sum_{(x_{\mathrm{M},j}, y_{\mathrm{M},j}) \in (S_{\mathrm{c},i} \cap \tilde{S}_\mathrm{p})} \int_{x_{\mathrm{M},j}-\frac{\Delta x_\mathrm{M}}{2}}^{x_{\mathrm{M},j}+\frac{\Delta x_\mathrm{M}}{2}} \int_{y_{\mathrm{M},j}-\frac{\Delta y_\mathrm{M}}{2}}^{y_{\mathrm{M},j}+\frac{\Delta y_\mathrm{M}}{2}} \left(\iint_{\mathbb{R}^2} h(\tau,\gamma) P(x - x_\mathrm{p} - \tau, y - y_\mathrm{p} - \gamma) \mathrm{d}\tau \mathrm{d}\gamma \right) \mathrm{d}x \mathrm{d}y. \tag{S26}$$

Under the Gaussian model of the illumination and emission PSF's, we can explicitly evaluate the integrals contained in the effective background B_i. For the convolution, we find:

$$\iint_{\mathbb{R}^2} h(\tau,\gamma) P_{\text{Gaussian}}(x - x_\mathrm{p} - \tau, y - y_\mathrm{p} - \gamma) \mathrm{d}\tau \mathrm{d}\gamma = \iint_{\mathbb{R}^2} \frac{1}{2\pi\sigma_{\mathrm{PSF}}^2} e^{\frac{-\tau^2-\gamma^2}{2\sigma_{\mathrm{PSF}}^2}} e^{\frac{-(x-x_\mathrm{p}-\tau)^2-(y-y_\mathrm{p}-\gamma)^2}{2\sigma_{\mathrm{illum}}^2}} \mathrm{d}\tau \mathrm{d}\gamma \tag{S27}$$

$$= \left(\int_{\mathbb{R}} \frac{1}{\sqrt{2\pi}\sigma_{\mathrm{PSF}}} e^{\frac{-\tau^2}{2\sigma_{\mathrm{PSF}}^2} + \frac{-(x-x_\mathrm{p}-\tau)^2}{2\sigma_{\mathrm{illum}}^2}} \mathrm{d}\tau \right) \cdot \left(\int_{\mathbb{R}} \frac{1}{\sqrt{2\pi}\sigma_{\mathrm{PSF}}} e^{\frac{-\gamma^2}{2\sigma_{\mathrm{PSF}}^2} + \frac{-(y-y_\mathrm{p}-\gamma)^2}{2\sigma_{\mathrm{illum}}^2}} \mathrm{d}\gamma \right) \tag{S28}$$

$$= \left(\frac{\sigma_{\mathrm{illum}}}{\sqrt{\sigma_{\mathrm{PSF}}^2 + \sigma_{\mathrm{illum}}^2}} e^{\frac{-(x-x_\mathrm{p})^2}{2\left(\sigma_{\mathrm{PSF}}^2+\sigma_{\mathrm{illum}}^2\right)}} \right) \cdot \left(\frac{\sigma_{\mathrm{illum}}}{\sqrt{\sigma_{\mathrm{PSF}}^2 + \sigma_{\mathrm{illum}}^2}} e^{\frac{-(y-y_\mathrm{p})^2}{2\left(\sigma_{\mathrm{PSF}}^2+\sigma_{\mathrm{illum}}^2\right)}} \right) \tag{S29}$$

$$= \frac{\sigma_{\mathrm{illum}}^2}{\sigma_{\mathrm{PSF}}^2 + \sigma_{\mathrm{illum}}^2} e^{\frac{-(x-x_\mathrm{p})^2-(y-y_\mathrm{p})^2}{2\left(\sigma_{\mathrm{PSF}}^2+\sigma_{\mathrm{illum}}^2\right)}} \tag{S30}$$

$$= 2\pi\sigma_{\mathrm{illum}}^2 \left(\frac{1}{2\pi(\sigma_{\mathrm{PSF}}^2 + \sigma_{\mathrm{illum}}^2)} e^{\frac{-(x-x_\mathrm{p})^2-(y-y_\mathrm{p})^2}{2\left(\sigma_{\mathrm{PSF}}^2+\sigma_{\mathrm{illum}}^2\right)}} \right). \tag{S31}$$

For the effective background $B_{i,\text{Gaussian}}$, we now have:

$$B_{i,\text{Gaussian}} = 2\pi\sigma_{\mathrm{illum}}^2 \sum_{(x_{\mathrm{M},j}, y_{\mathrm{M},j}) \in (S_{\mathrm{c},i} \cap \tilde{S}_\mathrm{p})} \int_{x_{\mathrm{M},j}-\frac{\Delta x_\mathrm{M}}{2}}^{x_{\mathrm{M},j}+\frac{\Delta x_\mathrm{M}}{2}} \int_{y_{\mathrm{M},j}-\frac{\Delta y_\mathrm{M}}{2}}^{y_{\mathrm{M},j}+\frac{\Delta y_\mathrm{M}}{2}} \frac{1}{2\pi\left(\sigma_{\mathrm{PSF}}^2 + \sigma_{\mathrm{illum}}^2\right)} e^{\frac{-(x-x_\mathrm{p})^2-(y-y_\mathrm{p})^2}{2\left(\sigma_{\mathrm{PSF}}^2+\sigma_{\mathrm{illum}}^2\right)}} \mathrm{d}x\mathrm{d}y \tag{S32}$$

$$= 2\pi\sigma_{\mathrm{illum}}^2 \sum_{(x_{\mathrm{M},j}, y_{\mathrm{M},j}) \in (S_{\mathrm{c},i} \cap \tilde{S}_\mathrm{p})} \underbrace{\left(\int_{x_{\mathrm{M},j}-\frac{\Delta x_\mathrm{M}}{2}}^{x_{\mathrm{M},j}+\frac{\Delta x_\mathrm{M}}{2}} \frac{1}{\sqrt{2\pi}\sqrt{\sigma_{\mathrm{PSF}}^2 + \sigma_{\mathrm{illum}}^2}} e^{\frac{-(x-x_\mathrm{p})^2}{2\left(\sigma_{\mathrm{PSF}}^2+\sigma_{\mathrm{illum}}^2\right)}} \mathrm{d}x \right)}_{E\left(x_{\mathrm{M},j}-x_\mathrm{p}, \Delta x_\mathrm{M}, \sigma_{\mathrm{PSF}}^2+\sigma_{\mathrm{illum}}^2\right)} \cdot \underbrace{\left(\int_{y_{\mathrm{M},j}-\frac{\Delta y_\mathrm{M}}{2}}^{y_{\mathrm{M},j}+\frac{\Delta y_\mathrm{M}}{2}} \frac{1}{\sqrt{2\pi}\sqrt{\sigma_{\mathrm{PSF}}^2 + \sigma_{\mathrm{illum}}^2}} e^{\frac{-(y-y_\mathrm{p})^2}{2\left(\sigma_{\mathrm{PSF}}^2+\sigma_{\mathrm{illum}}^2\right)}} \mathrm{d}y \right)}_{E\left(y_{\mathrm{M},j}-y_\mathrm{p}, \Delta y_\mathrm{M}, \sigma_{\mathrm{PSF}}^2+\sigma_{\mathrm{illum}}^2\right)}. \tag{S33}$$

Note that Equation (S33) requires us to compute definite integrals over Gaussian functions, as was also necessary in Equation (S20). As such, we can again use error functions to simplify the expression. Using the definition of $E(x, \Delta x, \sigma^2)$ from Equation (S21), we find:

$$E\left(x_{\mathrm{M},j}-x_{\mathrm{p}},\Delta x_{\mathrm{M}},\sigma_{\mathrm{PSF}}^2+\sigma_{\mathrm{illum}}^2\right)=\frac{1}{2}\mathrm{erf}\left(\frac{x_{\mathrm{M},j}-x_{\mathrm{p}}+\frac{\Delta x_{\mathrm{M}}}{2}}{\sqrt{2\sigma_{\mathrm{PSF}}^2+2\sigma_{\mathrm{illum}}^2}}\right)-\frac{1}{2}\mathrm{erf}\left(\frac{x_{\mathrm{M},j}-x_{\mathrm{p}}-\frac{\Delta x_{\mathrm{M}}}{2}}{\sqrt{2\sigma_{\mathrm{PSF}}^2+2\sigma_{\mathrm{illum}}^2}}\right), \tag{S34}$$

$$E\left(y_{\mathrm{M},j}-y_{\mathrm{p}},\Delta y_{\mathrm{M}},\sigma_{\mathrm{PSF}}^2+\sigma_{\mathrm{illum}}^2\right)=\frac{1}{2}\mathrm{erf}\left(\frac{y_{\mathrm{M},j}-y_{\mathrm{p}}+\frac{\Delta y_{\mathrm{M}}}{2}}{\sqrt{2\sigma_{\mathrm{PSF}}^2+2\sigma_{\mathrm{illum}}^2}}\right)-\frac{1}{2}\mathrm{erf}\left(\frac{y_{\mathrm{M},j}-y_{\mathrm{p}}-\frac{\Delta y_{\mathrm{M}}}{2}}{\sqrt{2\sigma_{\mathrm{PSF}}^2+2\sigma_{\mathrm{illum}}^2}}\right). \tag{S35}$$

In the end, we find the following expression for the effective background under Gaussian illumination:

$$B_{i,\mathrm{Gaussian}}=2\pi\sigma_{\mathrm{illum}}^2\sum_{(x_{\mathrm{M},j},y_{\mathrm{M},j})\in\left(S_{\mathrm{c},i}\cap\tilde{S}_{\mathrm{p}}\right)}E\left(x_{\mathrm{M},j}-x_{\mathrm{p}},\Delta x_{\mathrm{M}},\sigma_{\mathrm{PSF}}^2+\sigma_{\mathrm{illum}}^2\right)E\left(y_{\mathrm{M},j}-y_{\mathrm{p}},\Delta y_{\mathrm{M}},\sigma_{\mathrm{PSF}}^2+\sigma_{\mathrm{illum}}^2\right). \tag{S36}$$

The donut-shaped illumination pattern is not separable in x and y, which means we cannot separate the convolutions as was done in Equation (S28). For the convolution between the donut-shaped illumination pattern and the PSF, we find:

$$\iint_{\mathbb{R}^2} h(\tau,\gamma)P_{\mathrm{donut}}(x-x_{\mathrm{p}}-\tau,y-y_{\mathrm{p}}-\gamma)\mathrm{d}\tau\mathrm{d}\gamma \tag{S37}$$

$$=\iint_{\mathbb{R}^2}\frac{1}{2\pi\sigma_{\mathrm{PSF}}^2}e\cdot\left(\frac{(x-x_{\mathrm{p}})^2+(y-y_{\mathrm{p}})^2}{2\sigma_{\mathrm{illum}}^2}\right)e^{\frac{-(x-x_{\mathrm{p}})^2-(y-y_{\mathrm{p}})^2}{2\sigma_{\mathrm{illum}}^2}}\mathrm{d}\tau\mathrm{d}\gamma \tag{S38}$$

$$=e\cdot\frac{\sigma_{\mathrm{illum}}^2\left(\sigma_{\mathrm{illum}}^2\left(\left(x-x_{\mathrm{p}}\right)^2+\left(y-y_{\mathrm{p}}\right)^2+2\sigma_{\mathrm{PSF}}^2\right)+2\sigma_{\mathrm{PSF}}^4\right)}{2\left(\sigma_{\mathrm{PSF}}^2+\sigma_{\mathrm{illum}}^2\right)^3}e^{\frac{-(x-x_{\mathrm{p}})^2-(y-y_{\mathrm{p}})^2}{2\left(\sigma_{\mathrm{PSF}}^2+\sigma_{\mathrm{illum}}^2\right)}}. \tag{S39}$$

For the effective background $B_{i,\mathrm{donut}}$, we now have:

$$\begin{aligned}B_{i,\mathrm{donut}}=\sum_{(x_{\mathrm{M},j},y_{\mathrm{M},j})\in\left(S_{\mathrm{c},i}\cap\tilde{S}_{\mathrm{p}}\right)}&F\left(x_{\mathrm{M},j}+\frac{\Delta x_{\mathrm{M}}}{2},y_{\mathrm{M},j}+\frac{\Delta y_{\mathrm{M}}}{2}\right)-F\left(x_{\mathrm{M},j}+\frac{\Delta x_{\mathrm{M}}}{2},y_{\mathrm{M},j}-\frac{\Delta y_{\mathrm{M}}}{2}\right)\\&-F\left(x_{\mathrm{M},j}-\frac{\Delta x_{\mathrm{M}}}{2},y_{\mathrm{M},j}+\frac{\Delta y_{\mathrm{M}}}{2}\right)+F\left(x_{\mathrm{M},j}-\frac{\Delta x_{\mathrm{M}}}{2},y_{\mathrm{M},j}-\frac{\Delta y_{\mathrm{M}}}{2}\right).\end{aligned} \tag{S40}$$

where $F(x,y)$ is the function:

$$\begin{aligned}F(x,y)=&\frac{e\pi}{2}\sigma_{\mathrm{illum}}^2\mathrm{erf}\left(\frac{x-x_{\mathrm{p}}}{\sqrt{2\sigma_{\mathrm{PSF}}^2+2\sigma_{\mathrm{illum}}^2}}\right)\mathrm{erf}\left(\frac{y-y_{\mathrm{p}}}{\sqrt{2\sigma_{\mathrm{PSF}}^2+2\sigma_{\mathrm{illum}}^2}}\right)\\&-\frac{e\sqrt{\pi}\sigma_{\mathrm{illum}}^4}{\sqrt{\left(2\sigma_{\mathrm{PSF}}^2+2\sigma_{\mathrm{illum}}^2\right)^3}}\left(\left(x-x_{\mathrm{p}}\right)e^{\frac{-(x-x_{\mathrm{p}})^2}{2\sigma_{\mathrm{PSF}}^2+2\sigma_{\mathrm{illum}}^2}}\mathrm{erf}\left(\frac{y-y_{\mathrm{p}}}{\sqrt{2\sigma_{\mathrm{PSF}}^2+2\sigma_{\mathrm{illum}}^2}}\right)+\left(y-y_{\mathrm{p}}\right)e^{\frac{-(y-y_{\mathrm{p}})^2}{2\left(\sigma_{\mathrm{PSF}}^2+\sigma_{\mathrm{illum}}^2\right)}}\mathrm{erf}\left(\frac{x-x_{\mathrm{p}}}{\sqrt{2\left(\sigma_{\mathrm{PSF}}^2+\sigma_{\mathrm{illum}}^2\right)}}\right)\right).\end{aligned} \tag{S41}$$

Multiple illumination patterns

In Equation (S8), we assumed that only one illumination pattern is used for illumination. In SpinFlux, we have the opportunity to use multiple pinholes and patterns for illumination. In this subsection, we extend our image formation model to this situation.

For the image formation, we assume that pinholes are separated far enough on the spinning disk, such that only one pinhole can appear in a region of interest during each camera frame. This assumption is valid for the magnifications, pinhole sizes and

pinhole separations in existing SDCM setups (2, 14, 15). Accordingly, we assume there is no crosstalk between emission signal coming from different pinholes. This allows us to describe the regions of interest on the camera frames as separate regions of interest from individual patterns.

In the K-pattern case, the object on each camera frame is the result of single-pattern illumination, where the illumination patterns are centered at pinhole positions $\boldsymbol{x}_{\mathrm{p}} = [x_{\mathrm{p},1}, \ldots, x_{\mathrm{p},K}]$, $\boldsymbol{y}_{\mathrm{p}} = [y_{\mathrm{p},1}, \ldots, y_{\mathrm{p},K}]$, each corresponding to the pinhole with area $S_{\mathrm{p},k} = \left\{(x, y) | (x - x_{\mathrm{p},k})^2 + (y - y_{\mathrm{p},k})^2 \leq r^2_{\mathrm{p},k}\right\}$. This gives rise to the following object function $f_k(x, x_{\mathrm{p},k}, y, y_{\mathrm{p},k})$ for the object corresponding to pattern k:

$$f_k(x, x_{\mathrm{p},k}, y, y_{\mathrm{p},k}) = A_k P(x - x_{\mathrm{p},k}, y - y_{\mathrm{p},k})(\theta_I \delta(x - \theta_x, y - \theta_y) + \theta_b). \tag{S42}$$

In existing work on meSMLM, such as in MINFLUX (13), it is assumed that meSMLM is able to record the same amount of signal photons as SMLM. This assumption allows benchmarking between methods on the same signal photon count. However, the assumption is not trivial, as additional illumination power or time is needed to exhaust the signal photon budget with non-maximum illumination intensity. Properly adjusting the illumination power to compensate for the reduced photon flux requires accurate prior knowledge about the emitter position, which is generally unavailable, and is limited by saturation of the illumination intensity profile. Increasing the illumination time increases the probability of sample degradation. As such, it is reasonable to assume that meSMLM will not exhaust the signal photon budget completely.

The normalizing constant A_k describes how the signal photon budget is affected by non-maximum illumination. This constant plays a vital role in benchmarking meSMLM (when the summed intensity over all patterns does not result in a uniform profile), as it gives a physical explanation of the fair signal photon count against which meSMLM should be compared (8). Specifically when comparing meSMLM to SMLM, the normalization constant models whether meSMLM would have had recorded the same amount of signal photons as SMLM, despite the additional illumination power or time needed to do so. Results on the improvement of meSMLM compared to SMLM should thus only be given in the context of the normalizing constant A_k.

We choose A_k to model two different scenarios in this work, to explore how SpinFlux is affected by these conditions. In the first scenario, the entire signal photon budget is exhausted after illumination with all patterns (aside from signal photons that are blocked by the spinning disk), disregarding the illumination power and time needed to accomplish this for each pattern. This scenario is consistent with the assumption used in e.g. MINFLUX (13), stating that meSMLM will record the same amount of photons as SMLM. For this scenario, the sum of the illumination patterns should satisfy the conditions $\sum_{k=1}^{K} P(x - x_{\mathrm{p},k}, y - y_{\mathrm{p},k}) \geq 0$ for all $(x, y) \in \mathbb{R}^2$ and $\sum_{k=1}^{K} P(\theta_x - x_{\mathrm{p},k}, \theta_y - y_{\mathrm{p},k}) = 1$ to exhaust the expected signal photon budget on the emitter position. Under these conditions, $A_k = A$ is a constant applied equally to all patterns. If the individual patterns satisfy $P(x - x_{\mathrm{p},k}, y - y_{\mathrm{p},k}) \geq 0$ for all $(x, y) \in \mathbb{R}^2$, A must be given by Equation (S43) to satisfy the constraints on $\sum_{k=1}^{K} P(\theta_x - x_{\mathrm{p},k}, \theta_y - y_{\mathrm{p},k})$:

$$A_k = A = \frac{1}{\sum_{k=1}^{K} P(\theta_x - x_{\mathrm{p},k}, \theta_y - y_{\mathrm{p},k})} \tag{S43}$$

In the second scenario, the illumination power and time are constant for each pattern such that the total illumination power and time equal that of SMLM, even though this does not exhaust the signal photon budget for non-maximum illumination. For this scenario, the sum of the illumination patterns should satisfy the conditions $0 \leq \sum_{k=1}^{K} P(x - x_{\mathrm{p},k}, y - y_{\mathrm{p},k}) \leq 1$ for all $(x, y) \in \mathbb{R}^2$. If the individual patterns satisfy $0 \leq P(x - x_{\mathrm{p},k}, y - y_{\mathrm{p},k}) \leq 1$ for all $(x, y) \in \mathbb{R}^2$ and $P(x - x_{\mathrm{p},k}, y - y_{\mathrm{p},k}) = 1$ for some $(x, y) \in \mathbb{R}^2$, A must be given by Equation (S44) to satisfy the constraint on $\sum_{k=1}^{K} P(x - x_{\mathrm{p},k}, y - y_{\mathrm{p},k})$ for arbitrary x and y:

$$A_k = A = \frac{1}{K} \tag{S44}$$

We now continue the derivation of the image formation model. We approximate every pinhole area $S_{\mathrm{p},k}$ by the discretized pinhole $\tilde{S}_{\mathrm{p},k}$ following the discretization procedure described before. This gives the following model for the Poisson mean $\mu_{i,k}(x_i, x_{\mathrm{p}}, y_i, y_{\mathrm{p}})$ on pixel i with pinhole and pattern k:

$$\mu_{i,k}(x_i, x_{\mathrm{p},k}, y_i, y_{\mathrm{p},k}) = A\theta_I P(\theta_x - x_{\mathrm{p},k}, \theta_y - y_{\mathrm{p},k}) \sum_{(x_{\mathrm{M},j}, y_{\mathrm{M},j}) \in \left(S_{\mathrm{c},i} \cap \tilde{S}_{\mathrm{p},k}\right)} E(x_{\mathrm{M},j} - \theta_x, \Delta x_{\mathrm{M}}, \sigma^2_{\mathrm{PSF}}) E(y_{\mathrm{M},j} - \theta_y, \Delta y_{\mathrm{M}}, \sigma^2_{\mathrm{PSF}}) + A\theta_b B_{i,k}, \tag{S45}$$

$$B_{i,k} = 2\pi\sigma^2_{\mathrm{illum}} \sum_{(x_{\mathrm{M},j}, y_{\mathrm{M},j}) \in \left(S_{\mathrm{c},i} \cap \tilde{S}_{\mathrm{p},k}\right)} E\left(x_{\mathrm{M},j} - x_{\mathrm{p},k}, \Delta x_{\mathrm{M}}, \sigma^2_{\mathrm{PSF}} + \sigma^2_{\mathrm{illum}}\right) E\left(y_{\mathrm{M},j} - y_{\mathrm{p},k}, \Delta y_{\mathrm{M}}, \sigma^2_{\mathrm{PSF}} + \sigma^2_{\mathrm{illum}}\right) \tag{S46}$$

Pattern-independent background

In Equation (S8), we assumed that the illumination pattern modulates both the signal coming from the emitter PSF, as well as the background. In existing meSMLM work, such as in the analysis of e.g. MINFLUX (13), the pattern-dependency of the background is neglected. To allow for a fair comparison between these methods and SpinFlux, we derive and adapted image formation model, where the background is assumed to be pattern-independent. In this scenario, the object $f(x, x_\mathrm{p}, y, y_\mathrm{p})$ for a single pinhole and illumination pattern is modeled as follows:

$$f_{\text{patt.-indep. b.g.}}(x, x_\mathrm{p}, y, y_\mathrm{p}) = P(x - x_\mathrm{p}, y - y_\mathrm{p})(\theta_I \delta(x - \theta_x, y - \theta_y)) + \theta_b. \tag{S47}$$

Following the same derivation as for the pattern-dependent background, we find the following expression for the Poisson mean μ:

$$\mu_{i,\text{patt.-indep. b.g.}}(x_i, x_\mathrm{p}, y_i, y_\mathrm{p}) = \theta_I P(\theta_x - x_\mathrm{p}, \theta_y - y_\mathrm{p}) \sum_{(x_{\mathrm{M},j}, y_{\mathrm{M},j}) \in \left(S_{\mathrm{c},i} \cap \tilde{S}_{\mathrm{p}}\right)} E(x_{\mathrm{M},j} - \theta_x, \Delta x_{\mathrm{M}}, \sigma^2_{\mathrm{PSF}}) E(y_{\mathrm{M},j} - \theta_y, \Delta y_{\mathrm{M}}, \sigma^2_{\mathrm{PSF}}) + \theta_b B_{i,\text{patt.-indep. b.g.}}, \tag{S48}$$

$$B_{i,\text{patt.-indep. b.g.}} = \sum_{(x_{\mathrm{M},j}, y_{\mathrm{M},j}) \in \left(S_{\mathrm{c},i} \cap \tilde{S}_{\mathrm{p},k}\right)} \Delta x_\mathrm{M} \cdot \Delta y_\mathrm{M}. \tag{S49}$$

Note that for this case, the constant $B_{i,\text{patt.-indep. b.g.}}$ only depends on the intersection area $\left(S_{\mathrm{c},i} \cap \tilde{S}_\mathrm{p}\right)$ between the camera pixel i and the approximation of pinhole k.

To extend the model with pattern-independent background to multiple illumination patterns, we formulate the following object function:

$$f_{k,\text{patt.-indep. b.g.}}(x, x_{\mathrm{p},k}, y, y_{\mathrm{p},k}) = A_k P(x - x_{\mathrm{p},k}, y - y_{\mathrm{p},k})(\theta_I \delta(x - \theta_x, y - \theta_y)) + \theta_b. \tag{S50}$$

For the Poisson mean $\mu_{i,k,\text{patt.-indep. b.g.}}(x_i, x_\mathrm{p}, y_i, y_\mathrm{p})$ on pixel i for the camera frame with pattern k, this gives the following model:

$$\mu_{i,k,\text{patt.-indep. b.g.}}(x_i, x_{\mathrm{p},k}, y_i, y_{\mathrm{p},k}) = A\theta_I P(\theta_x - x_{\mathrm{p},k}, \theta_y - y_{\mathrm{p},k}) \sum_{(x_{\mathrm{M},j}, y_{\mathrm{M},j}) \in \left(S_{\mathrm{c},i} \cap \tilde{S}_{\mathrm{p},k}\right)} E(x_{\mathrm{M},j} - \theta_x, \Delta x_{\mathrm{M}}, \sigma^2_{\mathrm{PSF}}) E(y_{\mathrm{M},j} - \theta_y, \Delta y_{\mathrm{M}}, \sigma^2_{\mathrm{PSF}}) + \theta_b B_{i,\text{patt.-indep. b.g.}}, \tag{S51}$$

$$B_{i,\text{patt.-indep. b.g.}} = \sum_{(x_{\mathrm{M},j}, y_{\mathrm{M},j}) \in \left(S_{\mathrm{c},i} \cap \tilde{S}_{\mathrm{p},k}\right)} \Delta x_\mathrm{M} \cdot \Delta y_\mathrm{M}. \tag{S52}$$

Here, A is as in Equation (S43) for the scenario where the entire signal photon budget is exhausted after illumination with all patterns.

NOTE S3: CRAMÉR-RAO LOWER BOUND FOR SPINFLUX LOCALIZATION

In this note, we derive the Cramér-Rao lower bound (CRLB) for SpinFlux modulation enhanced single molecule localization microscopy.

Log-likelihood function for SpinFlux localization

In this subsection, we describe a statistical model for photon collection. The model should describe the amount of photons that are recorded by a camera pixel during a measurement, in the absence of readout noise. From (5), we infer that such a process can be modeled by the Poisson distribution. The Poisson distribution describes the amount of event occurrences within a certain time interval and it is therefore a proper probabilistic model for photon collection.
The Poisson process is dependent on a single parameter μ, which in our case describes the expected amount of photons that fall on a camera pixel during a measurement (see Supplementaty Note 1). The probability mass function $p(c)$ of the Poisson distribution is given by Equation (S53).

$$p(c) = \mathbb{P}(C = c) = \frac{\mu^c e^{-\mu}}{c!}. \tag{S53}$$

We start by considering single-pinhole and single-pattern SpinFlux. Let the random variables $\{C_i\}_{i=1}^{N_{\text{pixels}}}$ describe the amount of photons acquired by camera pixels $i \in \{1, 2, \ldots, N_{\text{pixels}}\}$ during a measurement with a pattern $P(x - x_{\text{p}}, y - y_{\text{p}})$. Therefore, $\{C_i\}_{i=1}^{N_{\text{pixels}}}$ can be considered mutually independent (5) and they all have a Poisson distribution with Poisson parameter $\mu_i(x_i, x_{\text{p}}, y_i, y_{\text{p}})$. Furthermore, let $\boldsymbol{c} = \{c_i\}_{i=1}^{N_{\text{pixels}}}$ denote the acquired measurements, which can be seen as realizations of $\{C_i\}_{i=1}^{N_{\text{pixels}}}$. We then find the Poisson likelihood $L(\boldsymbol{\theta}|\boldsymbol{c})$ and log-likelihood $\ell(\boldsymbol{\theta}|\boldsymbol{c})$ of Equations (S54) and (S55), respectively.

$$L(\boldsymbol{\theta}|\boldsymbol{c}) = \prod_{i=1}^{N_{\text{pixels}}} \frac{\mu_i^{c_i}(x_i, x_{\text{p}}, y_i, y_{\text{p}}) e^{-\mu_i(x_i, x_{\text{p}}, y_i, y_{\text{p}})}}{c_i!}, \tag{S54}$$

$$\ell(\boldsymbol{\theta}|\boldsymbol{c}) = \sum_{i=1}^{N_{\text{pixels}}} \left(c_i \log(\mu_i(x_i, x_{\text{p}}, y_i, y_{\text{p}})) - \log(c_i!) - \mu_i(x_i, x_{\text{p}}, y_i, y_{\text{p}}) \right). \tag{S55}$$

Cramér-Rao lower bound for SpinFlux localization

In this subsection, we compute the CRLB for single-pinhole and single-pattern SpinFlux, using the log-likelihood function of Equation (S55). For notation convenience, we leave out the arguments $(x_i, x_{\text{p}}, y_i, y_{\text{p}})$ of μ_i. We compute the partial derivative of $\ell(\boldsymbol{\theta}|\boldsymbol{c})$ with respect to the u'th element of $\boldsymbol{\theta}$, $\boldsymbol{\theta}_u$:

$$\frac{\partial \ell(\boldsymbol{\theta}|\boldsymbol{c})}{\partial \boldsymbol{\theta}_u} = \sum_{i=1}^{N_{\text{pixels}}} \left(c_i \frac{1}{\mu_i} \frac{\partial \mu_i}{\partial \boldsymbol{\theta}_u} - \frac{\partial \mu_i}{\partial \boldsymbol{\theta}_u} \right) \tag{S56}$$

$$= \sum_{k=1}^{N_{\text{pixels}}} \left((c_i - \mu_i) \frac{1}{\mu_i} \frac{\partial \mu_i}{\partial \boldsymbol{\theta}_u} \right) \tag{S57}$$

We can compute entry (u, v) of the Fisher information matrix as shown in Equation (S58) (16, 17).

$$I_{uv}(\boldsymbol{\theta}) = \mathbb{E}\left[\frac{\partial \ell(\boldsymbol{\theta}|\boldsymbol{c})}{\partial \boldsymbol{\theta}_u} \frac{\partial \ell(\boldsymbol{\theta}|\boldsymbol{c})}{\partial \boldsymbol{\theta}_v} \right] \tag{S58}$$

The Fisher information for single-pinhole and single-pattern SpinFlux is then given by:

$$I_{uv}(\boldsymbol{\theta}) = \mathbb{E}\left[\left(\sum_{i=1}^{N_{\text{pixels}}} (c_i - \mu_i)\frac{1}{\mu_i}\frac{\partial \mu_i}{\partial \boldsymbol{\theta}_u}\right)\left(\sum_{j=1}^{N_{\text{pixels}}} (c_j - \mu_j)\frac{1}{\mu_j}\frac{\partial \mu_j}{\partial \boldsymbol{\theta}_v}\right)\right] \tag{S59}$$

$$= \sum_{i=1}^{N_{\text{pixels}}}\sum_{j=1}^{N_{\text{pixels}}} \mathbb{E}\left[(c_i - \mu_i)(c_j - \mu_j)\frac{1}{\mu_i\mu_j}\frac{\partial \mu_i}{\partial \boldsymbol{\theta}_u}\frac{\partial \mu_j}{\partial \boldsymbol{\theta}_v}\right] \tag{S60}$$

$$= \sum_{i=1}^{N_{\text{pixels}}}\sum_{j=1}^{N_{\text{pixels}}} \frac{1}{\mu_i\mu_j}\frac{\partial \mu_i}{\partial \boldsymbol{\theta}_u}\frac{\partial \mu_j}{\partial \boldsymbol{\theta}_v}\mathbb{E}\left[(c_i - \mu_i)(c_j - \mu_j)\right] \tag{S61}$$

Note that $\mathbb{E}\left[(c_i - \mu_i)(c_j - \mu_j)\right]$ denotes the covariance of C_i and C_j. As $\{C_i\}_{i=1}^{N_{\text{pixels}}}$ were assumed to be mutually independent, the covariance is 0 if $i \neq j$ and it is equal to the variance if $i = j$. Furthermore, recall that the variance of a Poisson distribution is equal to its mean. We can hence express the Fisher information as shown in Equation (S62).

$$I_{uv}(\boldsymbol{\theta}) = \sum_{i=1}^{N_{\text{pixels}}} \frac{1}{\mu_i}\frac{\partial \mu_i}{\partial \boldsymbol{\theta}_u}\frac{\partial \mu_i}{\partial \boldsymbol{\theta}_v} \tag{S62}$$

The CRLB states that for any unbiased estimator $\hat{\boldsymbol{\theta}}$ of the parameter vector $\boldsymbol{\theta}$, $\left(\Sigma_{\hat{\boldsymbol{\theta}}} - I^{-1}(\boldsymbol{\theta})\right)$ is positive semi-definite (16). Here, $\Sigma_{\hat{\boldsymbol{\theta}}}$ denotes the estimator covariance, $I(\boldsymbol{\theta})$ is the Fisher information and $I^{-1}(\boldsymbol{\theta})$ is the CRLB. In particular, the diagonal of $I^{-1}(\boldsymbol{\theta})$ thus bounds the estimator variance from below.

Log-likelihood function and Cramér-Rao lower bound for multiple-pattern SpinFlux

In the multiple pattern case, the use of multiple single-pattern camera frames leads to an additional product term in the likelihood function of Equation (S54). This leads to the following log-likelihood function:

$$\ell(\boldsymbol{\theta}|\boldsymbol{c}) = \sum_{i=1}^{N_{\text{pixels}}}\sum_{k=1}^{K} \left(c_{i,k}\log(\mu_{i,k}(x_i, x_{\text{p},k}, y_i, y_{\text{p},k})) - \log(c_{i,k}!) - \mu_{i,k}(x_i, x_{\text{p},k}, y_i, y_{\text{p},k})\right). \tag{S63}$$

Analogous to the derivation of the single-pattern Fisher information, we find the following expression for the multiple-pattern case:

$$I_{uv}(\boldsymbol{\theta}) = \sum_{i=1}^{N_{\text{pixels}}}\sum_{k=1}^{K} \frac{1}{\mu_{i,k}}\frac{\partial \mu_{i,k}}{\partial \boldsymbol{\theta}_u}\frac{\partial \mu_{i,k}}{\partial \boldsymbol{\theta}_v} \tag{S64}$$

NOTE S4: DERIVATIVES OF THE SPINFLUX IMAGE FORMATION MODEL, NEEDED TO COMPUTE THE CRAMÉR-RAO LOWER BOUND

In this note, we derive expressions for $\frac{\partial \mu_i}{\partial \theta_x}$, $\frac{\partial \mu_i}{\partial \theta_y}$, $\frac{\partial \mu_i}{\partial \theta_I}$, and $\frac{\partial \mu_i}{\partial \theta_b}$, which allow us to compute the Fisher information from Equation (S62).

Derivative with respect to x-position (single pattern)

$$\begin{aligned}\frac{\partial \mu_i}{\partial \theta_x} &= \theta_I \frac{\partial P(\theta_x - x_\mathrm{p}, \theta_y - y_\mathrm{p})}{\partial \theta_x} \sum_{(x_{\mathrm{M},j}, y_{\mathrm{M},j}) \in (S_{\mathrm{c},i} \cap \tilde{S}_\mathrm{p})} E(x_{\mathrm{M},j} - \theta_x, \Delta x_\mathrm{M}, \sigma_\mathrm{PSF}^2) E(y_{\mathrm{M},j} - \theta_y, \Delta y_\mathrm{M}, \sigma_\mathrm{PSF}^2) \\ &+ \theta_I P(\theta_x - x_\mathrm{p}, \theta_y - y_\mathrm{p}) \sum_{(x_{\mathrm{M},j}, y_{\mathrm{M},j}) \in (S_{\mathrm{c},i} \cap \tilde{S}_\mathrm{p})} \frac{\partial E(x_{\mathrm{M},j} - \theta_x, \Delta x_\mathrm{M}, \sigma_\mathrm{PSF}^2)}{\partial \theta_x} E(y_{\mathrm{M},j} - \theta_y, \Delta y_\mathrm{M}, \sigma_\mathrm{PSF}^2)\end{aligned} \tag{S65}$$

Here, $\frac{\partial P(\theta_x - x_\mathrm{p}, \theta_y - y_\mathrm{p})}{\partial \theta_x}$ and $\frac{\partial E(x_{\mathrm{M},j} - \theta_x, \Delta x_\mathrm{M}, \sigma_\mathrm{PSF}^2)}{\partial \theta_x}$ are as follows:

$$\frac{\partial P_\mathrm{Gaussian}(\theta_x - x_\mathrm{p}, \theta_y - y_\mathrm{p})}{\partial \theta_x} = \left(\frac{x_\mathrm{p} - \theta_x}{\sigma_\mathrm{illum}^2}\right) e^{\frac{-(\theta_x - x_\mathrm{p})^2 - (\theta_y - y_\mathrm{p})^2}{2\sigma_\mathrm{illum}^2}} \tag{S66}$$

$$\frac{\partial P_\mathrm{donut}(\theta_x - x_\mathrm{p}, \theta_y - y_\mathrm{p})}{\partial \theta_x} = e\left(\frac{\theta_x - x_\mathrm{p}}{\sigma_\mathrm{illum}^2}\right) e^{\frac{-(\theta_x - x_\mathrm{p})^2 - (\theta_y - y_\mathrm{p})^2}{2\sigma_\mathrm{illum}^2}} + e\left(\frac{(\theta_x - x_\mathrm{p})^2 + (\theta_y - y_\mathrm{p})^2}{2\sigma_\mathrm{illum}^2}\right)\left(\frac{x_\mathrm{p} - \theta_x}{\sigma_\mathrm{illum}^2}\right) e^{\frac{-(\theta_x - x_\mathrm{p})^2 - (\theta_y - y_\mathrm{p})^2}{2\sigma_\mathrm{illum}^2}} \tag{S67}$$

$$\frac{\partial E(x_{\mathrm{M},j} - \theta_x, \Delta x_\mathrm{M}, \sigma_\mathrm{PSF}^2)}{\partial \theta_x} = \frac{1}{\sqrt{2\pi}\sigma_\mathrm{PSF}}\left(e^{\frac{-\left(x_{\mathrm{M},j} - \theta_x - \frac{\Delta x_\mathrm{M}}{2}\right)^2}{2\sigma_\mathrm{PSF}^2}} - e^{\frac{-\left(x_{\mathrm{M},j} - \theta_x + \frac{\Delta x_\mathrm{M}}{2}\right)^2}{2\sigma_\mathrm{PSF}^2}}\right) \tag{S68}$$

Derivative with respect to y-position (single pattern)

$$\begin{aligned}\frac{\partial \mu_i}{\partial \theta_y} &= \theta_I \frac{\partial P(\theta_x - x_\mathrm{p}, \theta_y - y_\mathrm{p})}{\partial \theta_y} \sum_{(x_{\mathrm{M},j}, y_{\mathrm{M},j}) \in (S_{\mathrm{c},i} \cap \tilde{S}_\mathrm{p})} E(x_{\mathrm{M},j} - \theta_x, \Delta x_\mathrm{M}, \sigma_\mathrm{PSF}^2) E(y_{\mathrm{M},j} - \theta_y, \Delta y_\mathrm{M}, \sigma_\mathrm{PSF}^2) \\ &+ \theta_I P(\theta_x - x_\mathrm{p}, \theta_y - y_\mathrm{p}) \sum_{(x_{\mathrm{M},j}, y_{\mathrm{M},j}) \in (S_{\mathrm{c},i} \cap \tilde{S}_\mathrm{p})} E(x_{\mathrm{M},j} - \theta_x, \Delta x_\mathrm{M}, \sigma_\mathrm{PSF}^2) \frac{\partial E(y_{\mathrm{M},j} - \theta_y, \Delta y_\mathrm{M}, \sigma_\mathrm{PSF}^2)}{\partial \theta_y}\end{aligned} \tag{S69}$$

Here, $\frac{\partial P(\theta_x - x_\mathrm{p}, \theta_y - y_\mathrm{p})}{\partial \theta_y}$ and $\frac{\partial E(y_{\mathrm{M},j} - \theta_y, \Delta y_\mathrm{M}, \sigma_\mathrm{PSF}^2)}{\partial \theta_y}$ are as follows:

$$\frac{\partial P_\mathrm{Gaussian}(\theta_x - x_\mathrm{p}, \theta_y - y_\mathrm{p})}{\partial \theta_y} = \left(\frac{y_\mathrm{p} - \theta_y}{\sigma_\mathrm{illum}^2}\right) e^{\frac{-(\theta_x - x_\mathrm{p})^2 - (\theta_y - y_\mathrm{p})^2}{2\sigma_\mathrm{illum}^2}} \tag{S70}$$

$$\frac{\partial P_\mathrm{donut}(\theta_x - x_\mathrm{p}, \theta_y - y_\mathrm{p})}{\partial \theta_y} = e\left(\frac{\theta_y - y_\mathrm{p}}{\sigma_\mathrm{illum}^2}\right) e^{\frac{-(\theta_x - x_\mathrm{p})^2 - (\theta_y - y_\mathrm{p})^2}{2\sigma_\mathrm{illum}^2}} + e\left(\frac{(\theta_x - x_\mathrm{p})^2 + (\theta_y - y_\mathrm{p})^2}{2\sigma_\mathrm{illum}^2}\right)\left(\frac{y_\mathrm{p} - \theta_y}{\sigma_\mathrm{illum}^2}\right) e^{\frac{-(\theta_x - x_\mathrm{p})^2 - (\theta_y - y_\mathrm{p})^2}{2\sigma_\mathrm{illum}^2}} \tag{S71}$$

$$\frac{\partial E(y_{\mathrm{M},j} - \theta_y, \Delta y_\mathrm{M}, \sigma_\mathrm{PSF}^2)}{\partial \theta_y} = \frac{1}{\sqrt{2\pi}\sigma_\mathrm{PSF}}\left(e^{\frac{-\left(y_{\mathrm{M},j} - \theta_y - \frac{\Delta y_\mathrm{M}}{2}\right)^2}{2\sigma_\mathrm{PSF}^2}} - e^{\frac{-\left(y_{\mathrm{M},j} - \theta_y + \frac{\Delta y_\mathrm{M}}{2}\right)^2}{2\sigma_\mathrm{PSF}^2}}\right) \tag{S72}$$

Derivative with respect to expected signal photon count (single pattern)

$$\frac{\partial \mu_i}{\partial \theta_I} = P(\theta_x - x_\mathrm{p}, \theta_y - y_\mathrm{p}) \sum_{(x_{\mathrm{M},j}, y_{\mathrm{M},j}) \in (S_{\mathrm{c},i} \cap \tilde{S}_\mathrm{p})} E(x_{\mathrm{M},j} - \theta_x, \Delta x_\mathrm{M}, \sigma^2_\mathrm{PSF}) E(y_{\mathrm{M},j} - \theta_y, \Delta y_\mathrm{M}, \sigma^2_\mathrm{PSF}) \tag{S73}$$

Derivative with respect to expected background count (single pattern)

$$\frac{\partial \mu_i}{\partial \theta_b} = B_i \tag{S74}$$

$$= 2\pi\sigma^2_\mathrm{illum} \sum_{(x_{\mathrm{M},j}, y_{\mathrm{M},j}) \in (S_{\mathrm{c},i} \cap \tilde{S}_\mathrm{p})} E\left(x_{\mathrm{M},j} - x_\mathrm{p}, \Delta x_\mathrm{M}, \sigma^2_\mathrm{PSF} + \sigma^2_\mathrm{illum}\right) E\left(y_{\mathrm{M},j} - y_\mathrm{p}, \Delta y_\mathrm{M}, \sigma^2_\mathrm{PSF} + \sigma^2_\mathrm{illum}\right) \tag{S75}$$

Derivative with respect to x-position (multiple pattern)

$$\begin{aligned} \frac{\partial \mu_{i,k}}{\partial \theta_x} &= A\theta_I \frac{\partial P(\theta_x - x_{\mathrm{p},k}, \theta_y - y_{\mathrm{p},k})}{\partial \theta_x} \sum_{(x_{\mathrm{M},j}, y_{\mathrm{M},j}) \in (S_{\mathrm{c},i} \cap \tilde{S}_{\mathrm{p},k})} E(x_{\mathrm{M},j} - \theta_x, \Delta x_\mathrm{M}, \sigma^2_\mathrm{PSF}) E(y_{\mathrm{M},j} - \theta_y, \Delta y_\mathrm{M}, \sigma^2_\mathrm{PSF}) \\ &+ A\theta_I P(\theta_x - x_{\mathrm{p},k}, \theta_y - y_{\mathrm{p},k}) \sum_{(x_{\mathrm{M},j}, y_{\mathrm{M},j}) \in (S_{\mathrm{c},i} \cap \tilde{S}_{\mathrm{p},k})} \frac{\partial E(x_{\mathrm{M},j} - \theta_x, \Delta x_\mathrm{M}, \sigma^2_\mathrm{PSF})}{\partial \theta_x} E(y_{\mathrm{M},j} - \theta_y, \Delta y_\mathrm{M}, \sigma^2_\mathrm{PSF}) \end{aligned} \tag{S76}$$

Here, $\frac{\partial P(\theta_x - x_{\mathrm{p},k}, \theta_y - y_{\mathrm{p},k})}{\partial \theta_x}$ is as follows:

$$\frac{\partial P_\mathrm{Gaussian}(\theta_x - x_{\mathrm{p},k}, \theta_y - y_{\mathrm{p},k})}{\partial \theta_x} = \left(\frac{x_{\mathrm{p},k} - \theta_x}{\sigma^2_\mathrm{illum}}\right) e^{\frac{-(\theta_x - x_{\mathrm{p},k})^2 - (\theta_y - y_{\mathrm{p},k})^2}{2\sigma^2_\mathrm{illum}}} \tag{S77}$$

$$\begin{aligned} \frac{\partial P_\mathrm{donut}(\theta_x - x_{\mathrm{p},k}, \theta_y - y_{\mathrm{p},k})}{\partial \theta_x} &= e\left(\frac{\theta_x - x_{\mathrm{p},k}}{\sigma^2_\mathrm{illum}}\right) e^{\frac{-(\theta_x - x_{\mathrm{p},k})^2 - (\theta_y - y_{\mathrm{p},k})^2}{2\sigma^2_\mathrm{illum}}} \\ &+ e\left(\frac{(\theta_x - x_{\mathrm{p},k})^2 + (\theta_y - y_{\mathrm{p},k})^2}{2\sigma^2_\mathrm{illum}}\right)\left(\frac{x_{\mathrm{p},k} - \theta_x}{\sigma^2_\mathrm{illum}}\right) e^{\frac{-(\theta_x - x_{\mathrm{p},k})^2 - (\theta_y - y_{\mathrm{p},k})^2}{2\sigma^2_\mathrm{illum}}} \end{aligned} \tag{S78}$$

$\frac{\partial E(x_{\mathrm{M},j} - \theta_x, \Delta x_\mathrm{M}, \sigma^2_\mathrm{PSF})}{\partial \theta_x}$ remains unchanged from Equation (S68).

Derivative with respect to y-position (multiple pattern)

$$\begin{aligned} \frac{\partial \mu_{i,k}}{\partial \theta_y} &= A\theta_I \frac{\partial P(\theta_x - x_{\mathrm{p},k}, \theta_y - y_{\mathrm{p},k})}{\partial \theta_y} \sum_{(x_{\mathrm{M},j}, y_{\mathrm{M},j}) \in (S_{\mathrm{c},i} \cap \tilde{S}_{\mathrm{p},k})} E(x_{\mathrm{M},j} - \theta_x, \Delta x_\mathrm{M}, \sigma^2_\mathrm{PSF}) E(y_{\mathrm{M},j} - \theta_y, \Delta y_\mathrm{M}, \sigma^2_\mathrm{PSF}) \\ &+ A\theta_I P(\theta_x - x_{\mathrm{p},k}, \theta_y - y_{\mathrm{p},k}) \sum_{(x_{\mathrm{M},j}, y_{\mathrm{M},j}) \in (S_{\mathrm{c},i} \cap \tilde{S}_{\mathrm{p},k})} E(x_{\mathrm{M},j} - \theta_x, \Delta x_\mathrm{M}, \sigma^2_\mathrm{PSF}) \frac{\partial E(y_{\mathrm{M},j} - \theta_y, \Delta y_\mathrm{M}, \sigma^2_\mathrm{PSF})}{\partial \theta_y} \end{aligned} \tag{S79}$$

Here, $\frac{\partial P(\theta_x - x_{\mathrm{p},k}, \theta_y - y_{\mathrm{p},k})}{\partial \theta_y}$ is as follows:

$$\frac{\partial P_{\text{Gaussian}}(\theta_x - x_{\text{p},k}, \theta_y - y_{\text{p},k})}{\partial \theta_y} = \left(\frac{y_{\text{p},k} - \theta_y}{\sigma^2_{\text{illum}}}\right) e^{\frac{-(\theta_x - x_{\text{p},k})^2 - (\theta_y - y_{\text{p},k})^2}{2\sigma^2_{\text{illum}}}} \tag{S80}$$

$$\begin{aligned}\frac{\partial P_{\text{donut}}(\theta_x - x_{\text{p},k}, \theta_y - y_{\text{p},k})}{\partial \theta_y} &= e\left(\frac{\theta_y - y_{\text{p},k}}{\sigma^2_{\text{illum}}}\right) e^{\frac{-(\theta_x - x_{\text{p},k})^2 - (\theta_y - y_{\text{p},k})^2}{2\sigma^2_{\text{illum}}}} \\ &+ e\left(\frac{(\theta_x - x_{\text{p},k})^2 + (\theta_y - y_{\text{p},k})^2}{2\sigma^2_{\text{illum}}}\right)\left(\frac{y_{\text{p},k} - \theta_y}{\sigma^2_{\text{illum}}}\right) e^{\frac{-(\theta_x - x_{\text{p},k})^2 - (\theta_y - y_{\text{p},k})^2}{2\sigma^2_{\text{illum}}}}\end{aligned} \tag{S81}$$

$\frac{\partial E(y_{\text{M},j} - \theta_y, \Delta y_{\text{M}}, \sigma^2_{\text{PSF}})}{\partial \theta_y}$ remains unchanged from Equation (S72).

Derivative with respect to expected signal photon count (multiple pattern)

$$\frac{\partial \mu_{i,k}}{\partial \theta_I} = AP(\theta_x - x_{\text{p},k}, \theta_y - y_{\text{p},k}) \sum_{(x_{\text{M},j}, y_{\text{M},j}) \in (S_{\text{c},i} \cap \tilde{S}_{\text{p},k})} E(x_{\text{M},j} - \theta_x, \Delta x_{\text{M}}, \sigma^2_{\text{PSF}}) E(y_{\text{M},j} - \theta_y, \Delta y_{\text{M}}, \sigma^2_{\text{PSF}}) \tag{S82}$$

Derivative with respect to expected background count (multiple pattern)

$$\frac{\partial \mu_{i,k}}{\partial \theta_b} = AB_{i,k} \tag{S83}$$

$$= 2\pi\sigma^2_{\text{illum}} A \sum_{(x_{\text{M},j}, y_{\text{M},j}) \in (S_{\text{c},i} \cap \tilde{S}_{\text{p},k})} E\left(x_{\text{M},j} - x_{\text{p},k}, \Delta x_{\text{M}}, \sigma^2_{\text{PSF}} + \sigma^2_{\text{illum}}\right) E\left(y_{\text{M},j} - y_{\text{p},k}, \Delta y_{\text{M}}, \sigma^2_{\text{PSF}} + \sigma^2_{\text{illum}}\right) \tag{S84}$$

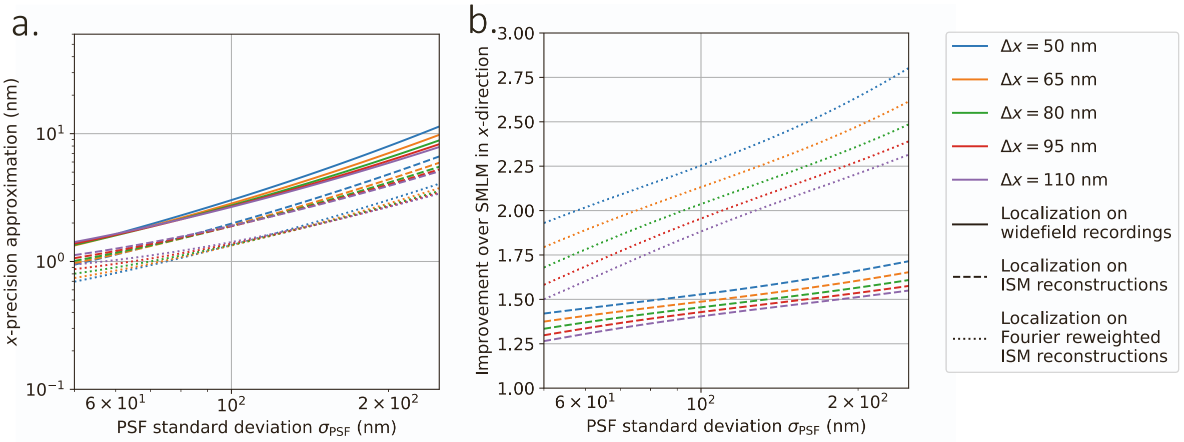

Figure S1: Approximation of the theoretical minimum localization uncertainty of single-molecule localization microscopy (SMLM) reconstructions acquired from (Fourier reweighted) image scanning microscopy (ISM). For this simulation, 2000 expected signal photons and 8 expected background photons per pixel were used. **(a)** Approximate CRLB in x-direction as a function of the PSF standard deviation for varying camera pixel sizes. **(b)** Improvement of the approximate CRLB over SMLM as a function of the PSF standard deviation for varying camera pixel sizes.

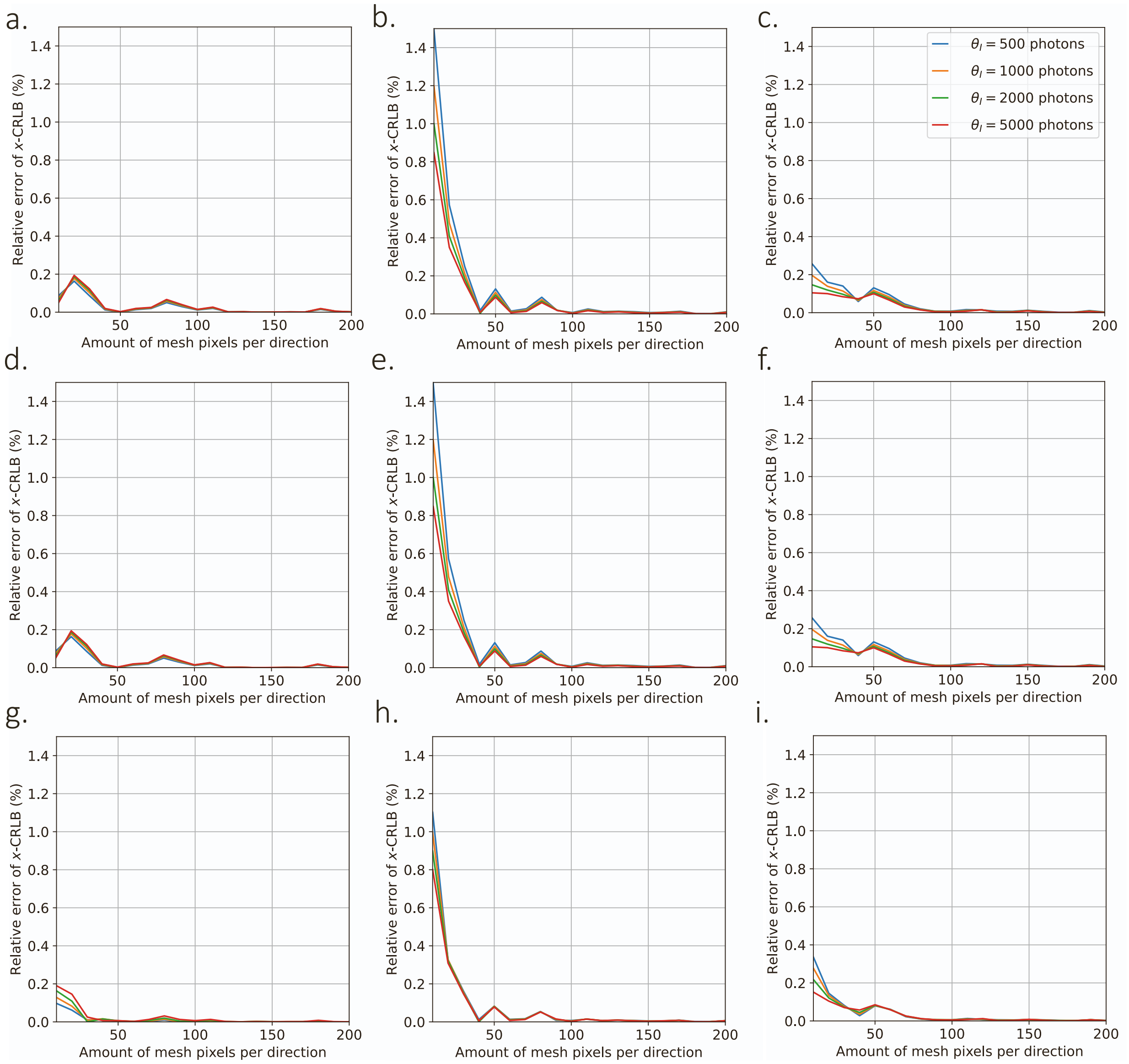

Figure S2: Relative error in the x-Cramér-Rao lower bound (CRLB) resulting from the discretized pinhole approximation as a function of the amount of mesh pixels $N_{\mathrm{M},x}$, $N_{\mathrm{M},y}$ in each direction. To determine the error, the CRLB approximation for a fine-mesh pinhole approximation with $N_{\mathrm{M},x}, N_{\mathrm{M},y} = 1000$ mesh pixels was assumed as the ground truth. $\theta_b = 8$ expected background photons per pixel were used and the expected signal photon count θ_I is varied. **(a, b, c)** Relative errors for the scenario where the entire signal photon budget is exhausted after illumination with all patterns (disregarding signal photons blocked by the spinning disk). **(d, e, f)** Relative errors for the scenario where the illumination power and time are constant during illumination with all patterns. **(g, h, i)** Relative errors for the scenario where the entire signal photon budget is exhausted after illumination with all patterns (disregarding signal photons blocked by the spinning disk), neglecting the effects of pattern-dependent background. **(a, d, g)** Relative errors for the one-pattern configuration, with pinhole radius $r_{\mathrm{p}} = 3\sigma_{\mathrm{PSF}}$ and pinhole position $(x_{\mathrm{p}}, y_{\mathrm{p}}) = (\theta_x, \theta_y)$. **(b, e, h)** Relative errors for the two-pattern configuration, separated in x, with pinhole radius $r_{\mathrm{p}} = 3\sigma_{\mathrm{PSF}}$, pinhole separation $s = 4\sigma_{\mathrm{PSF}}$ and focus position $(x_{\mathrm{f}}, y_{\mathrm{f}}) = (\theta_x, \theta_y)$. **(c, f, g)** Relative errors for the non-rotated equilateral triangle pattern configuration, with pinhole radius $r_{\mathrm{p}} = 3\sigma_{\mathrm{PSF}}$, pinhole spacing $r = 2\sigma_{\mathrm{PSF}}$ and focus position $(x_{\mathrm{f}}, y_{\mathrm{f}}) = (\theta_x, \theta_y)$.

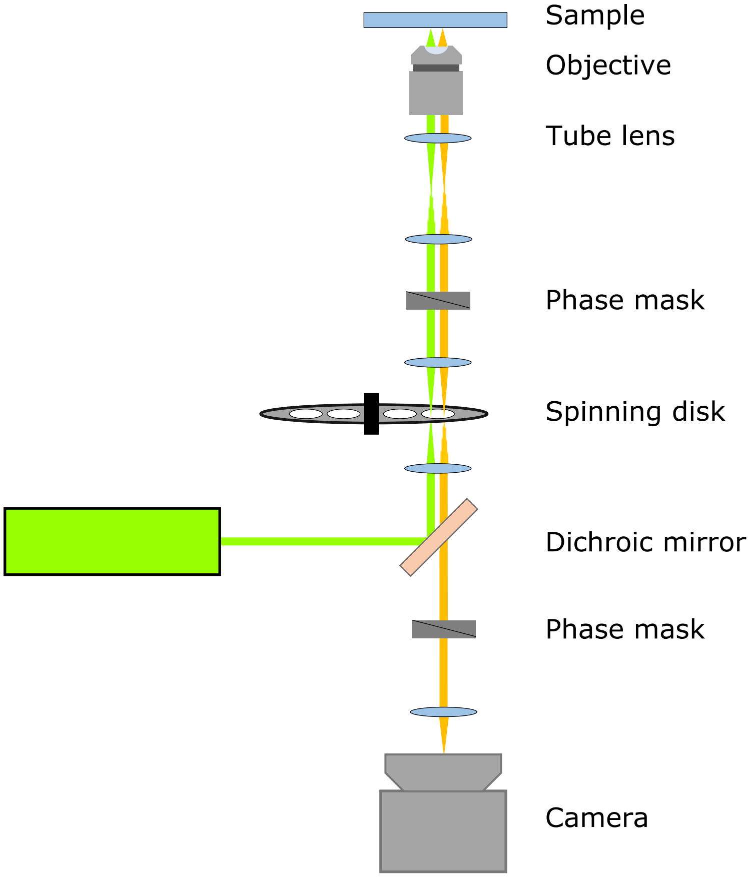

Figure S3: Schematic overview of SpinFlux image formation with donut-shaped illumination patterns. A spinning disk is placed in the illumination- and emission paths. This causes patterned illumination of emitters in the sample and subsequent windowing of the emission signal. Rapidly switching the laser on and off causes stroboscopic illumination of emitters in the sample with stationary illumination patterns. A phase mask in the illumination path modulates the illumination pattern into a donut-shaped beam. As the emission path also passes through the phase mask, the emission signal is demodulated using an additional phase mask in the emission path.

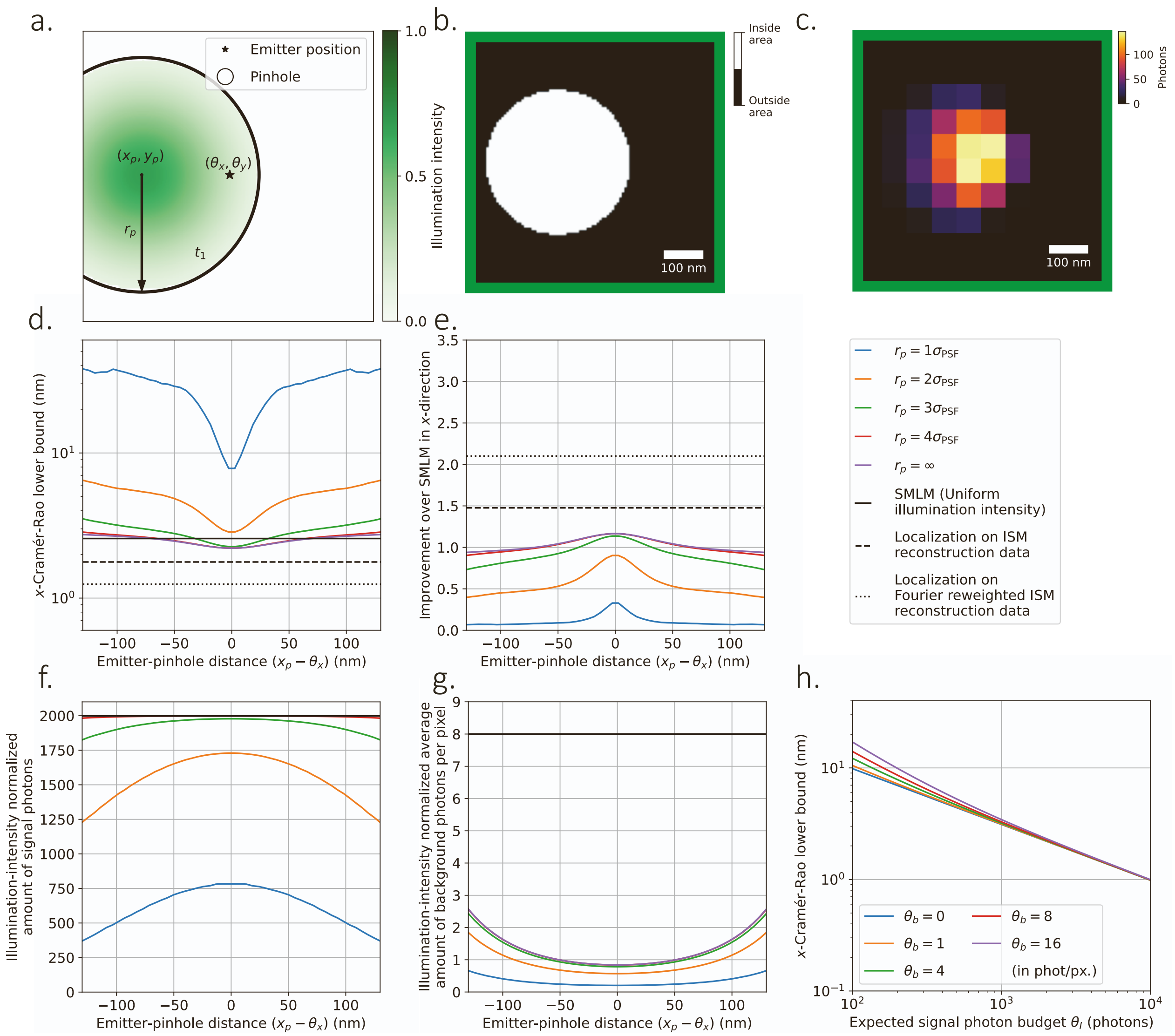

Figure S4: Theoretical minimum localization uncertainty of SpinFlux localization with one x-offset pinhole and pattern. In (c-g), 2000 expected signal photons and 8 expected background photons per pixel were used. Results are evaluated for the scenario where the entire signal photon budget is exhausted after illumination with the pattern (disregarding signal photons blocked by the spinning disk). **(a)** Schematic overview of SpinFlux localization with one pinhole with radius r_p, centered at coordinates (x_p, y_p). In (d-g), the x-distance $(x_\text{p} - \theta_x)$ between the pinhole and the emitter is varied, where $y_\text{p} = \theta_y$. **(b)** Example of pinhole in the region of interest (650 × 650 nm). The pinhole radius $r_\text{p} = 2\sigma_\text{PSF}$ was used. The pinhole mask was discretized with $N_{\text{M},x}, N_{\text{M},y} = 100$ mesh pixels in each direction. **(c)** Example of fluorescent response in the region of interest, resulting from illumination and emission through the pinhole in (b). **(d)** Cramér-Rao lower bound (CRLB) in x-direction as a function of the emitter-pinhole x-distance. Simulations show SpinFlux with varying pinhole sizes and widefield single-molecule localization microscopy (SMLM). **(e)** Improvement of the SpinFlux CRLB over SMLM as a function of the emitter-pinhole x-distance for varying pinhole sizes. **(f)** Average amount of signal photons after compensation for non-maximum illumination intensity as a function of the emitter-pinhole x-distance, for SpinFlux with varying pinhole sizes and widefield single molecule localization microscopy (SMLM). **(g)** Average amount of background photons per pixel after compensation for non-maximum illumination intensity as a function of the emitter-pinhole x-distance, for SpinFlux with varying pinhole sizes and widefield single molecule localization microscopy (SMLM). **(h)** CRLB in x-direction as a function of the expected signal photon count for varying values of the expected background photon count. The pinhole radius $r_\text{p} = 3\sigma_\text{PSF}$ was used and $(x_\text{p}, y_\text{p}) = (\theta_x, \theta_y)$.

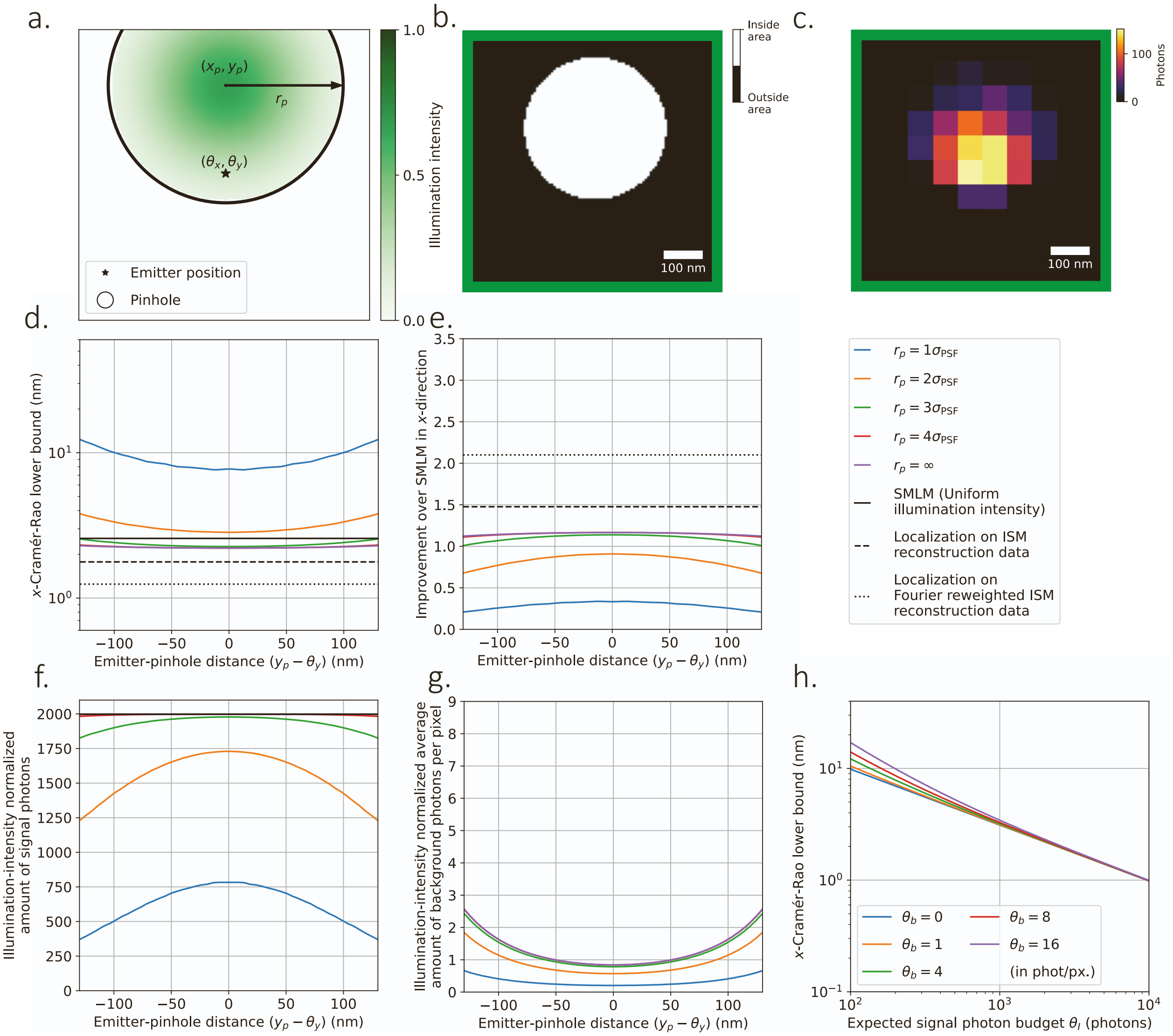

Figure S5: Theoretical minimum localization uncertainty of SpinFlux localization with one y-offset pinhole and pattern. In (c-g), 2000 expected signal photons and 8 expected background photons per pixel were used. Results are evaluated for the scenario where the entire signal photon budget is exhausted after illumination with the pattern (disregarding signal photons blocked by the spinning disk). **(a)** Schematic overview of SpinFlux localization with one pinhole with radius r_p, centered at coordinates $(x_\mathrm{p}, y_\mathrm{p})$. In (d-g), the y-distance $(y_\mathrm{p} - \theta_y)$ between the pinhole and the emitter is varied, where $x_\mathrm{p} = \theta_x$. **(b)** Example of pinhole in the region of interest (650 × 650 nm). The pinhole radius $r_\mathrm{p} = 2\sigma_\mathrm{PSF}$ was used. The pinhole mask was discretized with $N_{\mathrm{M},x}$, $N_{\mathrm{M},y}$ = 100 mesh pixels in each direction. **(c)** Example of fluorescent response in the region of interest, resulting from illumination and emission through the pinhole in (b). **(d)** Cramér-Rao lower bound (CRLB) in x-direction as a function of the emitter-pinhole y-distance. Simulations show SpinFlux with varying pinhole sizes and widefield single-molecule localization microscopy (SMLM). **(e)** Improvement of the SpinFlux CRLB over SMLM as a function of the emitter-pinhole y-distance for varying pinhole sizes. **(f)** Average amount of signal photons after compensation for non-maximum illumination intensity as a function of the emitter-pinhole y-distance, for SpinFlux with varying pinhole sizes and widefield single molecule localization microscopy (SMLM). **(g)** Average amount of background photons per pixel after compensation for non-maximum illumination intensity as a function of the emitter-pinhole y-distance, for SpinFlux with varying pinhole sizes and widefield single molecule localization microscopy (SMLM). **(h)** CRLB in x-direction as a function of the expected signal photon count for varying values of the expected background photon count. The pinhole radius $r_\mathrm{p} = 3\sigma_\mathrm{PSF}$ was used and $(x_\mathrm{p}, y_\mathrm{p}) = (\theta_x, \theta_y)$.

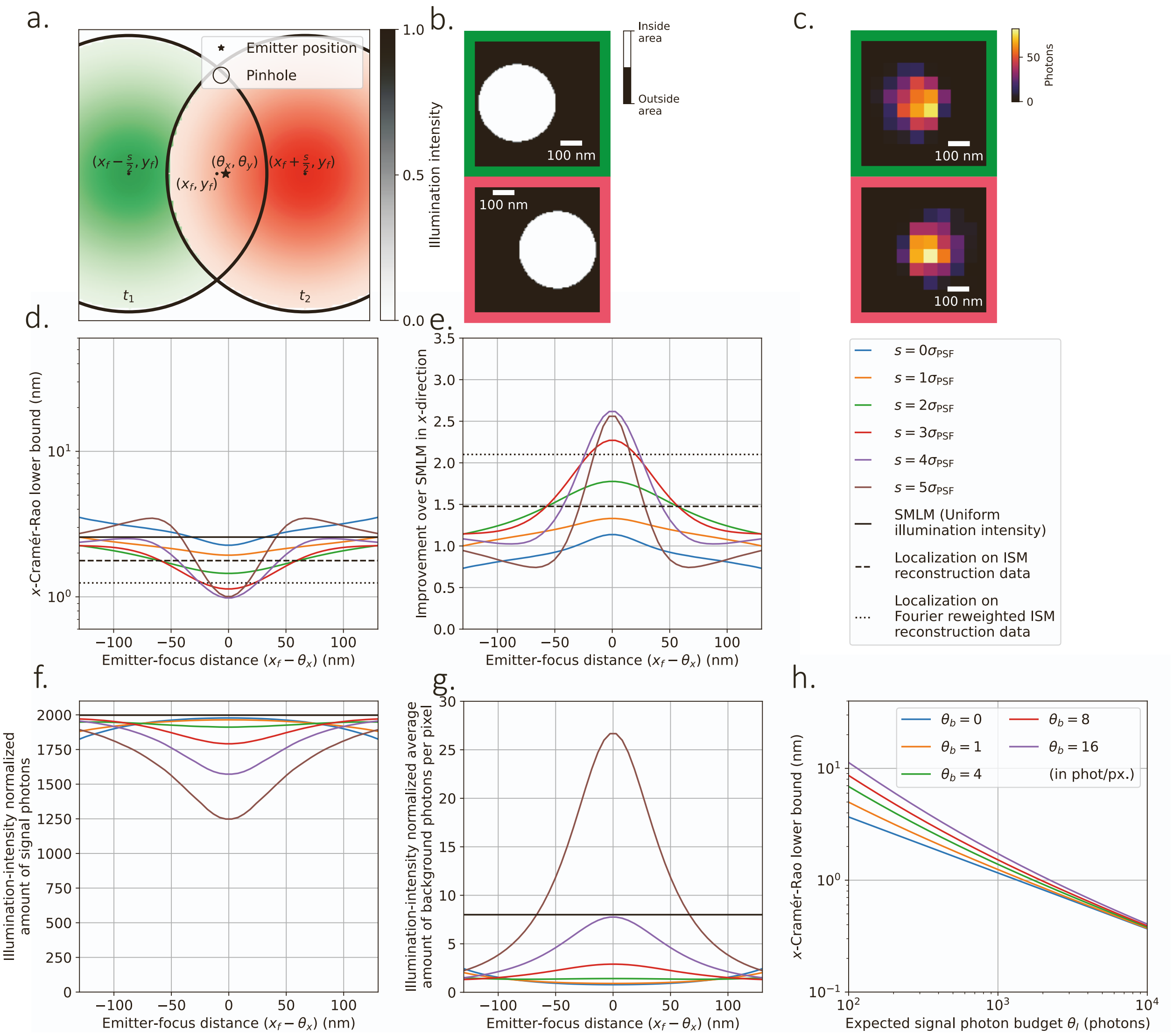

Figure S6: Theoretical minimum localization uncertainty of SpinFlux localization with two pinholes and patterns separated in the x-direction. In (c-g), 2000 expected signal photons and 8 expected background photons per pixel were used, with pinhole radius $r_{\mathrm{p}} = 3\sigma_{\mathrm{PSF}}$. Results are evaluated for the scenario where the entire signal photon budget is exhausted after illumination with all patterns (disregarding signal photons blocked by the spinning disk). **(a)** Schematic overview of SpinFlux localization with two pinholes, separated in x and centered around the focus coordinates $(x_{\mathrm{f}}, y_{\mathrm{f}})$. In (d-g), the x-distance $(x_{\mathrm{f}} - \theta_x)$ between the pattern focus and the emitter is varied, where $y_{\mathrm{f}} = \theta_y$. **(b)** Example of pinholes in the region of interest (650×650 nm). The pinhole radius $r_{\mathrm{p}} = 2\sigma_{\mathrm{PSF}}$ and pinhole separation $s = 2\sigma_{\mathrm{PSF}}$ were used. The pinhole masks were discretized with $N_{\mathrm{M},x}, N_{\mathrm{M},y} = 100$ mesh pixels in each direction. **(c)** Example of fluorescent response in the region of interest, resulting from illumination and emission through each pinhole in (b). **(d)** Cramér-Rao lower bound (CRLB) in x-direction as a function of the emitter-focus x-distance. Simulations show SpinFlux with varying pinhole separations and widefield single molecule localization microscopy (SMLM). **(e)** Improvement of the SpinFlux CRLB over SMLM as a function of the emitter-focus x-distance for varying pinhole separations. **(f)** Average amount of signal photons after compensation for non-maximum illumination intensity as a function of the emitter-focus x-distance, for SpinFlux with varying pinhole separations and widefield single molecule localization microscopy (SMLM). **(g)** Average amount of background photons per pixel after compensation for non-maximum illumination intensity as a function of the emitter-focus x-distance, for SpinFlux with varying pinhole separations and widefield single molecule localization microscopy (SMLM). **(h)** CRLB in x-direction as a function of expected signal photon count for varying values of the expected background photon count. The pinhole radius $r_{\mathrm{p}} = 3\sigma_{\mathrm{PSF}}$ and pinhole separation $s = 4\sigma_{\mathrm{PSF}}$ were used and $(x_{\mathrm{f}}, y_{\mathrm{f}}) = (\theta_x, \theta_y)$.

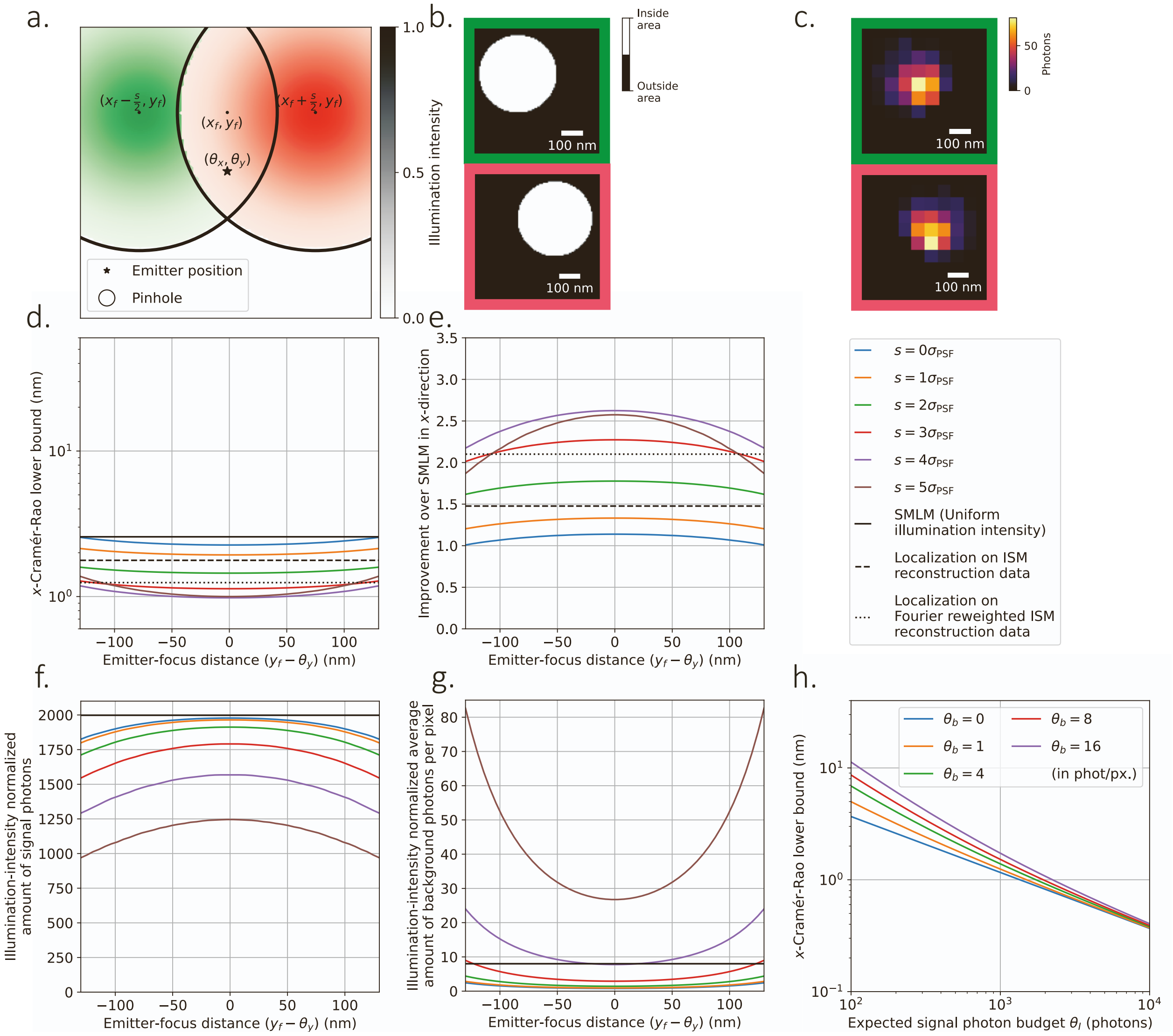

Figure S7: Theoretical minimum localization uncertainty of SpinFlux localization with two y-offset pinholes and patterns separated in the x-direction. In (c-g), 2000 expected signal photons and 8 expected background photons per pixel were used, with pinhole radius $r_p = 3\sigma_{PSF}$. Results are evaluated for the scenario where the entire signal photon budget is exhausted after illumination with all patterns (disregarding signal photons blocked by the spinning disk). **(a)** Schematic overview of SpinFlux localization with two pinholes, separated in x and centered around the focus coordinates (x_f, y_f). In (d-g), the y-distance $(y_f - \theta_y)$ between the pattern focus and the emitter is varied, where $x_f = \theta_x$. **(b)** Example of pinholes in the region of interest (650×650 nm). The pinhole radius $r_p = 2\sigma_{PSF}$ and pinhole separation $s = 2\sigma_{PSF}$ were used. The pinhole masks were discretized with $N_{M,x}$, $N_{M,y} = 100$ mesh pixels in each direction. **(c)** Example of fluorescent response in the region of interest, resulting from illumination and emission through each pinhole in (b). **(d)** Cramér-Rao lower bound (CRLB) in x-direction as a function of the emitter-focus y-distance. Simulations show SpinFlux with varying pinhole separations and widefield single molecule localization microscopy (SMLM). **(e)** Improvement of the SpinFlux CRLB over SMLM as a function of the emitter-focus y-distance for varying pinhole separations. **(f)** Average amount of signal photons after compensation for non-maximum illumination intensity as a function of the emitter-focus y-distance, for SpinFlux with varying pinhole separations and widefield single molecule localization microscopy (SMLM). **(g)** Average amount of background photons per pixel after compensation for non-maximum illumination intensity as a function of the emitter-focus y-distance, for SpinFlux with varying pinhole separations and widefield single molecule localization microscopy (SMLM). **(h)** CRLB in x-direction as a function of expected signal photon count for varying values of the expected background photon count. The pinhole radius $r_p = 3\sigma_{PSF}$ and pinhole separation $s = 4\sigma_{PSF}$ were used and $(x_f, y_f) = (\theta_x, \theta_y)$.

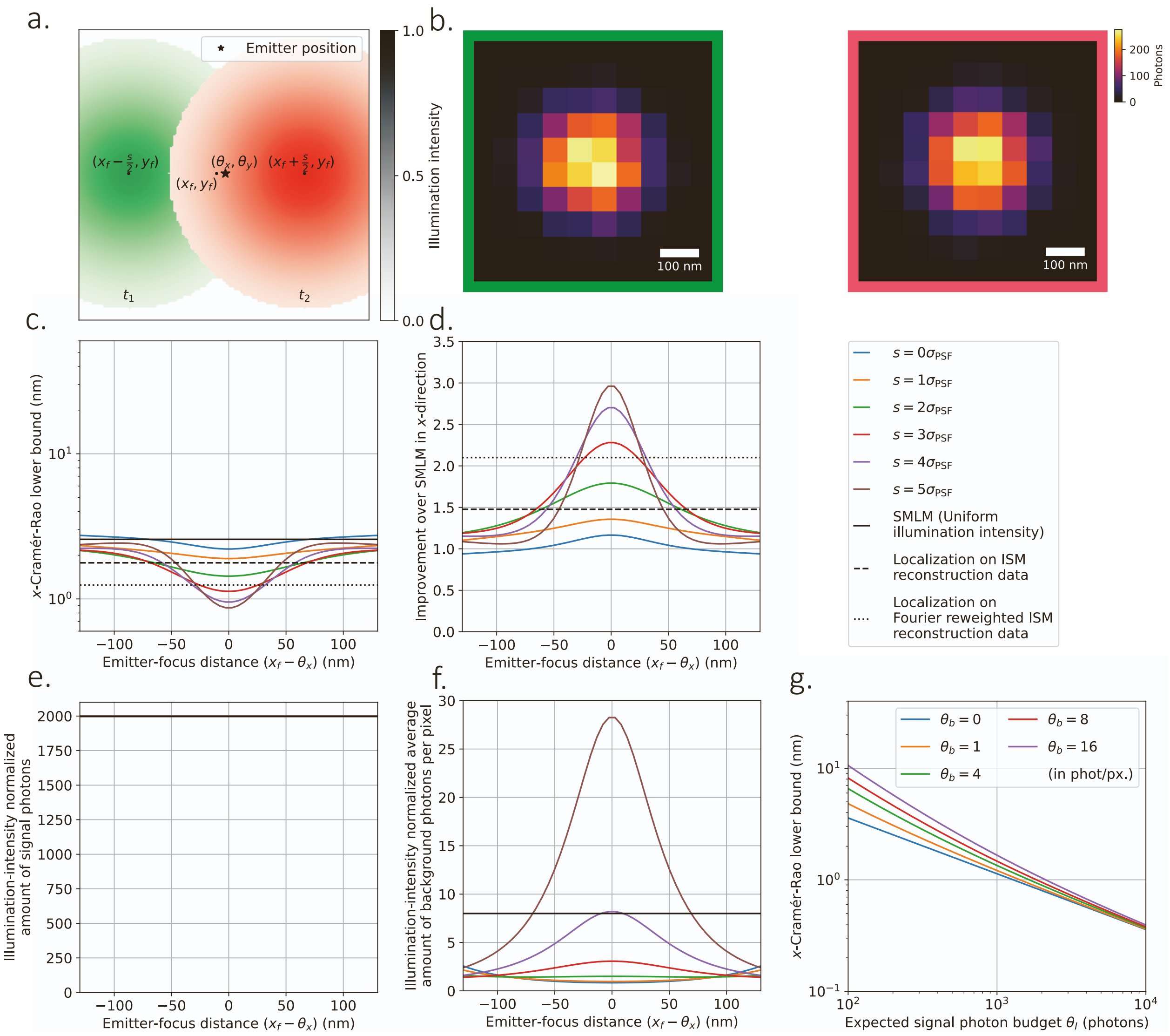

Figure S8: Theoretical minimum localization uncertainty of SpinFlux localization with two patterns without pinholes separated in the x-direction. In (b-f), 2000 expected signal photons and 8 expected background photons per pixel were used. Results are evaluated for the scenario where the entire signal photon budget is exhausted after illumination with all patterns. **(a)** Schematic overview of SpinFlux localization with two pinholes, separated in x and centered around the focus coordinates (x_f, y_f). In (c-f), the x-distance $(x_\text{f} - \theta_x)$ between the pattern focus and the emitter is varied, where $y_\text{f} = \theta_y$. **(b)** Example of fluorescent response in the region of interest, resulting from illumination and emission by each pattern in (a). **(c)** Cramér-Rao lower bound (CRLB) in x-direction as a function of the emitter-focus x-distance. Simulations show SpinFlux with varying pinhole separations and widefield single molecule localization microscopy (SMLM). **(d)** Improvement of the SpinFlux CRLB over SMLM as a function of the emitter-focus x-distance for varying pinhole separations. **(e)** Average amount of signal photons after compensation for non-maximum illumination intensity as a function of the emitter-focus x-distance, for SpinFlux with varying pinhole separations and widefield single molecule localization microscopy (SMLM). **(f)** Average amount of background photons per pixel after compensation for non-maximum illumination intensity as a function of the emitter-focus x-distance, for SpinFlux with varying pinhole separations and widefield single molecule localization microscopy (SMLM). **(g)** CRLB in x-direction as a function of expected signal photon count for varying values of the expected background photon count. The pattern separation $s = 4\sigma_\text{PSF}$ was used and $(x_\text{f}, y_\text{f}) = (\theta_x, \theta_y)$.

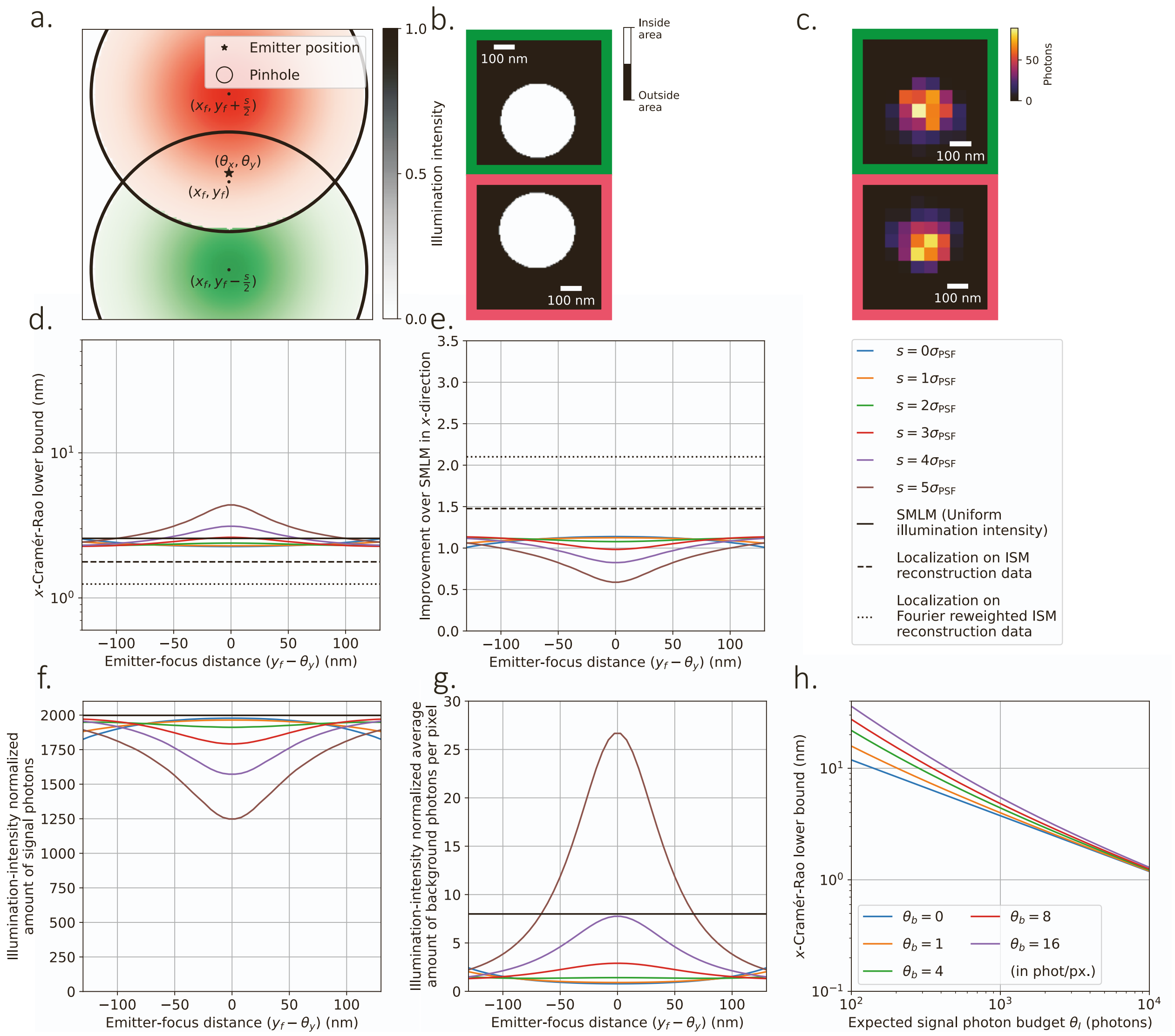

Figure S9: Theoretical minimum localization uncertainty of SpinFlux localization with two pinholes and patterns separated in the y-direction. In (c-g), 2000 expected signal photons and 8 expected background photons per pixel were used, with pinhole radius $r_p = 3\sigma_{\mathrm{PSF}}$. Results are evaluated for the scenario where the entire signal photon budget is exhausted after illumination with all patterns (disregarding signal photons blocked by the spinning disk). **(a)** Schematic overview of SpinFlux localization with two pinholes, separated in y and centered around the focus coordinates (x_f, y_f). In (d-g), the y-distance $(y_f - \theta_y)$ between the pattern focus and the emitter is varied, where $x_f = \theta_x$. **(b)** Example of pinholes in the region of interest (650 × 650 nm). The pinhole radius $r_p = 2\sigma_{\mathrm{PSF}}$ and pinhole separation $s = 2\sigma_{\mathrm{PSF}}$ were used. The pinhole masks were discretized with $N_{\mathrm{M},x}, N_{\mathrm{M},y} = 100$ mesh pixels in each direction. **(c)** Example of fluorescent response in the region of interest, resulting from illumination and emission through each pinhole in (b). **(d)** Cramér-Rao lower bound (CRLB) in x-direction as a function of the emitter-focus y-distance. Simulations show SpinFlux with varying pinhole separations and widefield single molecule localization microscopy (SMLM). **(e)** Improvement of the SpinFlux CRLB over SMLM as a function of the emitter-focus y-distance for varying pinhole separations. **(f)** Average amount of signal photons after compensation for non-maximum illumination intensity as a function of the emitter-focus y-distance, for SpinFlux with varying pinhole separations and widefield single molecule localization microscopy (SMLM). **(g)** Average amount of background photons per pixel after compensation for non-maximum illumination intensity as a function of the emitter-focus y-distance, for SpinFlux with varying pinhole separations and widefield single molecule localization microscopy (SMLM). **(h)** CRLB in x-direction as a function of expected signal photon count for varying values of the expected background photon count. The pinhole radius $r_p = 3\sigma_{\mathrm{PSF}}$ and pinhole separation $s = 4\sigma_{\mathrm{PSF}}$ were used and $(x_f, y_f) = (\theta_x, \theta_y)$.

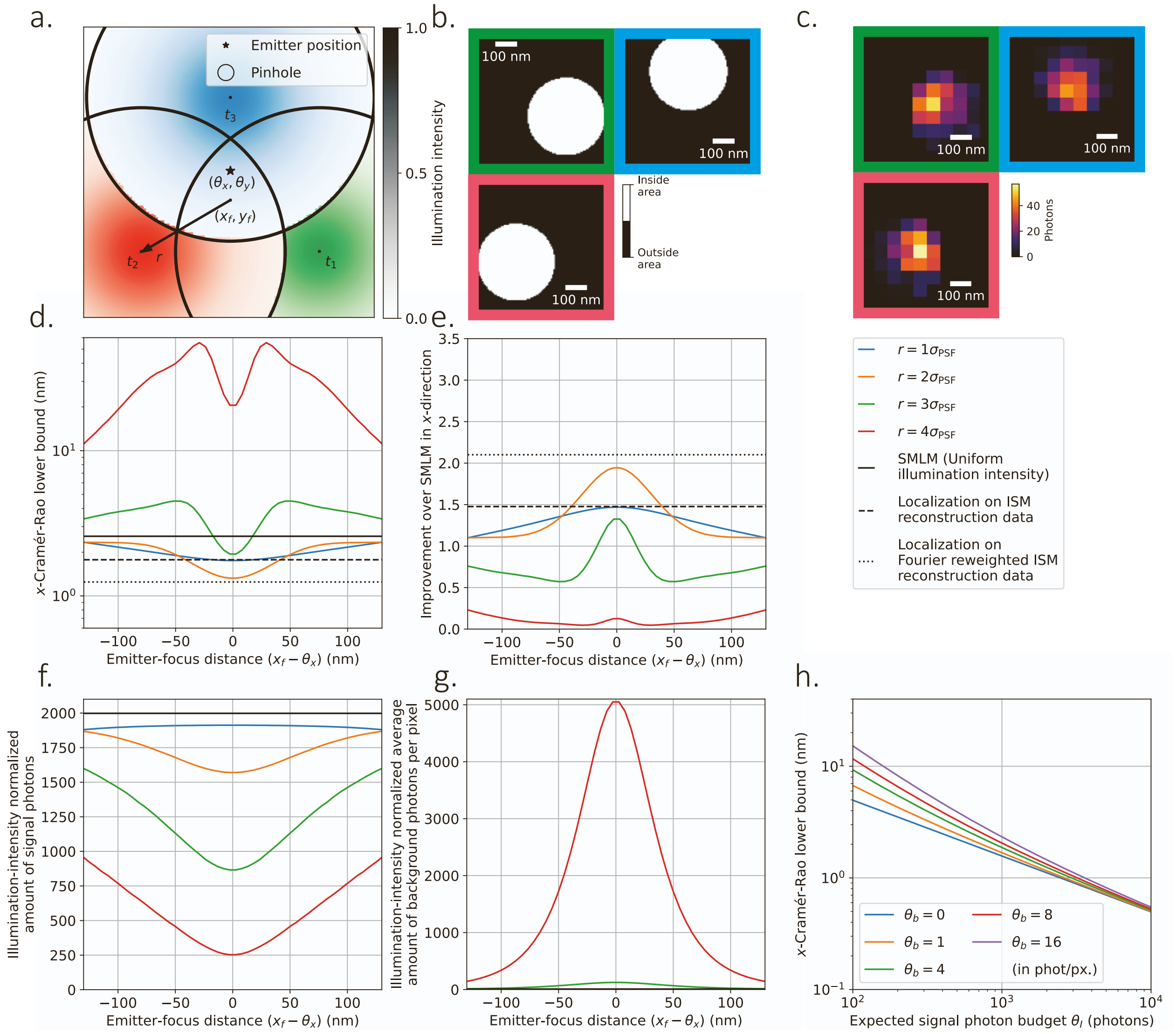

Figure S10: Theoretical minimum localization uncertainty of SpinFlux localization with three pinholes and patterns in an equilateral triangle configuration. In (c-g), we used 2000 expected signal photons and 8 expected background photons per pixel, with pinhole radius $r_p = 3\sigma_{PSF}$. Results are evaluated for the scenario where the entire signal photon budget is exhausted after illumination with all patterns (disregarding signal photons blocked by the spinning disk). **(a)** Schematic overview of SpinFlux localization with a triangle of three pinholes, centered at focus coordinates (x_f, y_f). In (d-g), the x-distance $(x_f - \theta_x)$ between the pattern focus and the emitter is varied, where $y_f = \theta_y$. **(b)** Example of pinholes in the region of interest (650 × 650 nm). The pinhole radius $r_p = 2\sigma_{PSF}$ and pinhole spacing $r = 1.5\sigma_{PSF}$ were used. The pinhole masks were discretized with $N_{M,x}, N_{M,y} = 100$ mesh pixels in each direction. **(c)** Example of fluorescent response in the region of interest, resulting from illumination and emission through each pinhole in (b). **(d)** Cramér-Rao lower bound (CRLB) in x-direction as a function of the emitter-focus x-distance. Simulations show SpinFlux with varying pinhole spacing and widefield single molecule localization microscopy (SMLM). **(e)** Improvement of the SpinFlux CRLB over SMLM as a function of the emitter-focus x-distance for varying pinhole spacing. **(f)** Average amount of signal photons after compensation for non-maximum illumination intensity as a function of the emitter-focus x-distance, for SpinFlux with varying pinhole spacing and widefield single molecule localization microscopy (SMLM). **(g)** Average amount of background photons per pixel after compensation for non-maximum illumination intensity as a function of the emitter-focus x-distance, for SpinFlux with varying pinhole spacing and widefield single molecule localization microscopy (SMLM). **(h)** CRLB in x-direction as a function of expected signal photon count for varying values of the expected background photon count. The pinhole radius $r_p = 3\sigma_{PSF}$ and pinhole spacing $r = 2\sigma_{PSF}$ were used and $(x_f, y_f) = (\theta_x, \theta_y)$.

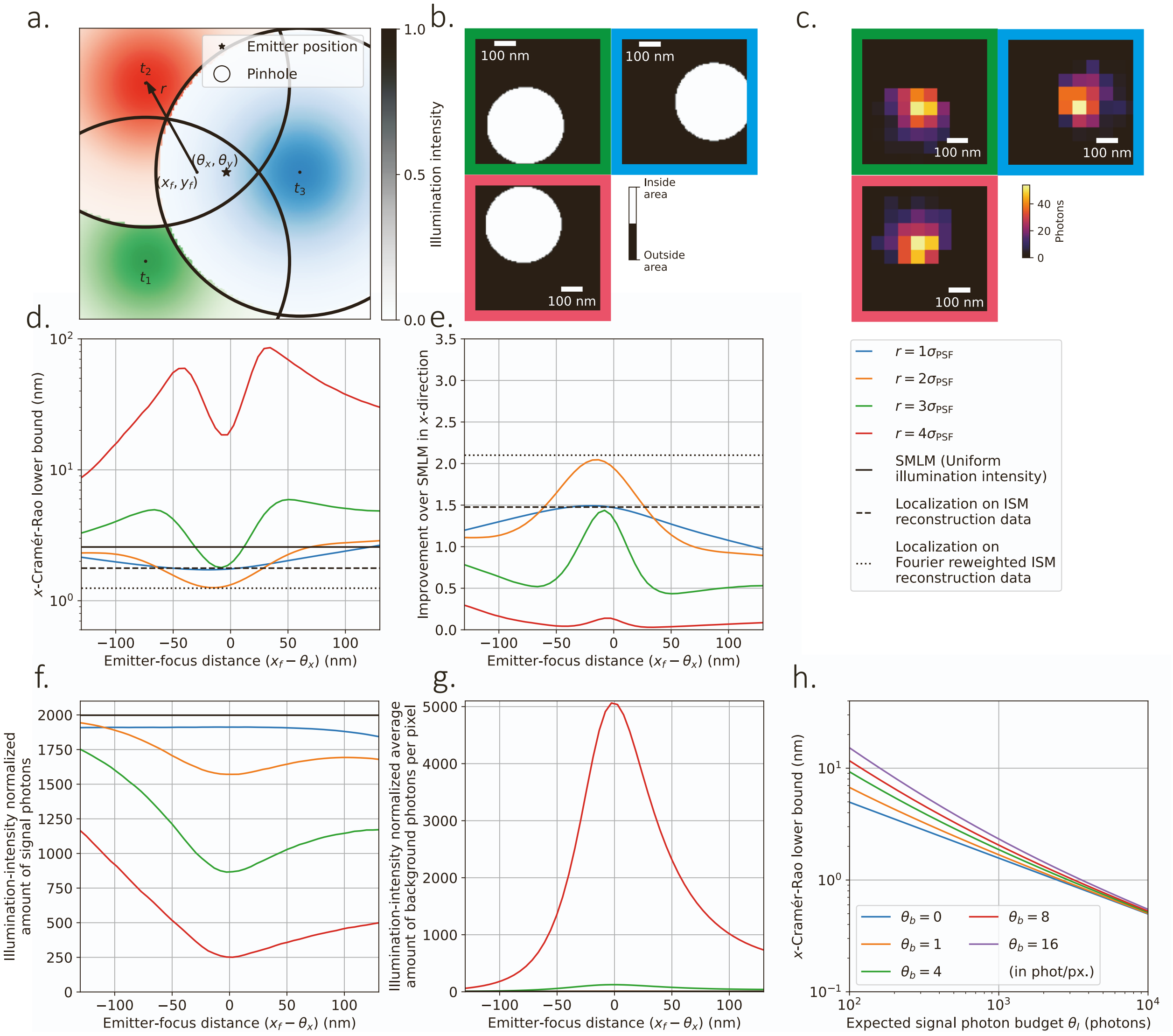

Figure S11: Theoretical minimum localization uncertainty of SpinFlux localization with three pinholes and patterns in a 90° rotated equilateral triangle configuration. The pattern is rotated clockwise by 90 degrees with respect Figure S10. In (c-g), we used 2000 expected signal photons and 8 expected background photons per pixel, with pinhole radius $r_p = 3\sigma_{PSF}$. Results are evaluated for the scenario where the entire signal photon budget is exhausted after illumination with all patterns (disregarding signal photons blocked by the spinning disk). **(a)** Schematic overview of SpinFlux localization with a triangle of three pinholes, centered at focus coordinates (x_f, y_f). In (d-g), the x-distance $(x_f - \theta_x)$ between the pattern focus and the emitter is varied, where $y_f = \theta_y$. **(b)** Example of pinholes in the region of interest (650 × 650 nm). The pinhole radius $r_p = 2\sigma_{PSF}$ and pinhole spacing $r = 1.5\sigma_{PSF}$ were used. The pinhole masks were discretized with $N_{M,x}$, $N_{M,y}$ = 100 mesh pixels in each direction. **(c)** Example of fluorescent response in the region of interest, resulting from illumination and emission through each pinhole in (b). **(d)** Cramér-Rao lower bound (CRLB) in x-direction as a function of the emitter-focus x-distance. Simulations show SpinFlux with varying pinhole spacing and widefield single molecule localization microscopy (SMLM). **(e)** Improvement of the SpinFlux CRLB over SMLM as a function of the emitter-focus x-distance for varying pinhole spacing. **(f)** Average amount of signal photons after compensation for non-maximum illumination intensity as a function of the emitter-focus x-distance, for SpinFlux with varying pinhole spacing and widefield single molecule localization microscopy (SMLM). **(g)** Average amount of background photons per pixel after compensation for non-maximum illumination intensity as a function of the emitter-focus x-distance, for SpinFlux with varying pinhole spacing and widefield single molecule localization microscopy (SMLM). **(h)** CRLB in x-direction as a function of expected signal photon count for varying values of the expected background photon count. The pinhole radius $r_p = 3\sigma_{PSF}$ and pinhole spacing $r = 2\sigma_{PSF}$ were used and $(x_f, y_f) = (\theta_x, \theta_y)$.

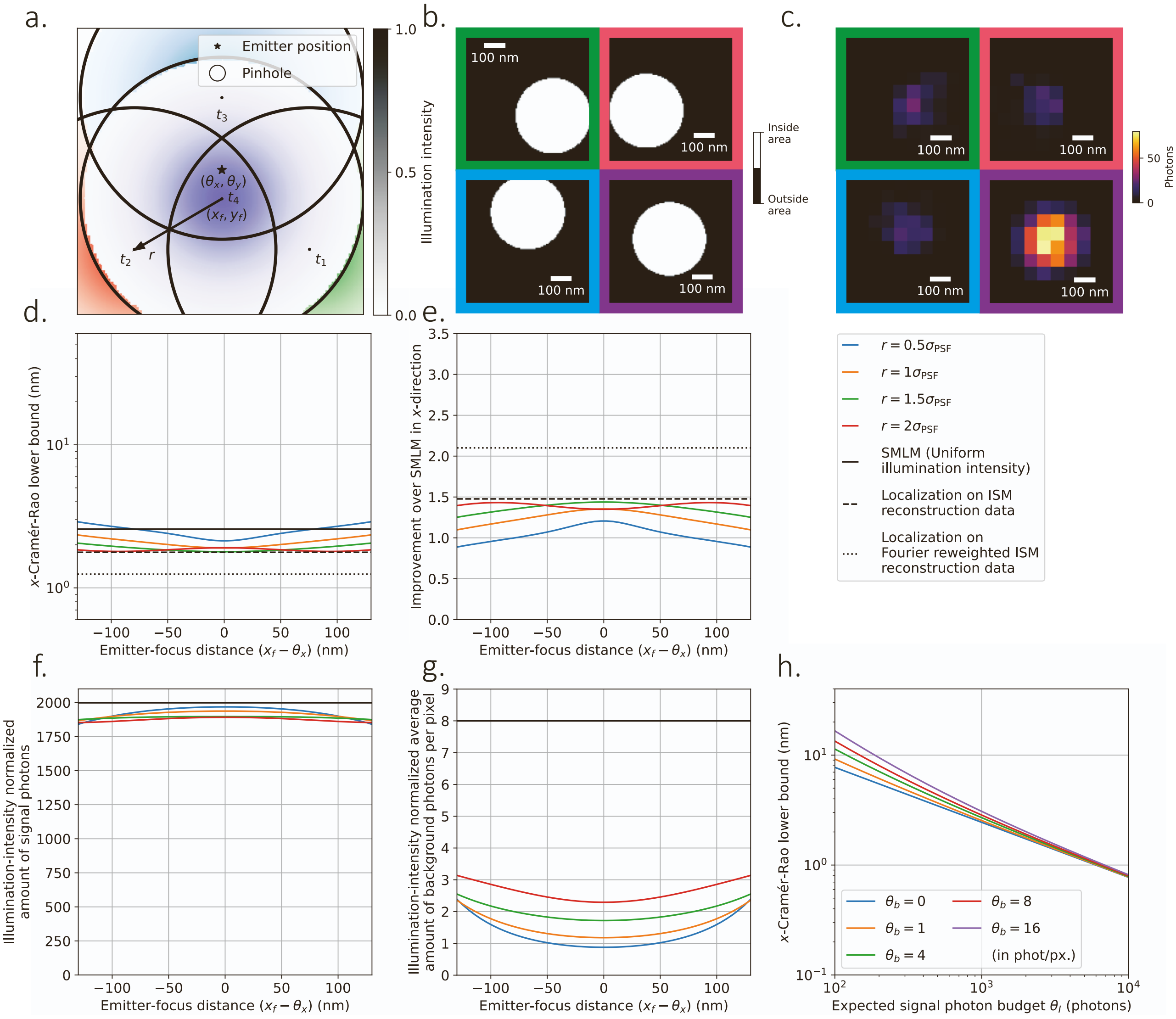

Figure S12: Theoretical minimum localization uncertainty of SpinFlux localization with four pinholes and patterns in an equilateral triangle configuration with a center pinhole. In (c-g), we used 2000 expected signal photons and 8 expected background photons per pixel, with pinhole radius $r_p = 3\sigma_{PSF}$. Results are evaluated for the scenario where the entire signal photon budget is exhausted after illumination with all patterns (disregarding signal photons blocked by the spinning disk). **(a)** Schematic overview of SpinFlux localization with a triangle of three pinholes with an additional center pinhole, centered at focus coordinates (x_f, y_f). In (d-g), the x-distance $(x_f - \theta_x)$ between the pattern focus and the emitter is varied, where $y_f = \theta_y$. **(b)** Example of pinholes in the region of interest (650 × 650 nm). The pinhole radius $r_p = 2\sigma_{PSF}$ and pinhole spacing $r = 1.5\sigma_{PSF}$ were used. The pinhole masks were discretized with $N_{M,x}, N_{M,y} = 100$ mesh pixels in each direction. **(c)** Example of fluorescent response in the region of interest, resulting from illumination and emission through each pinhole in (b). **(d)** Cramér-Rao lower bound (CRLB) in x-direction as a function of the emitter-focus x-distance. Simulations show SpinFlux with varying pinhole spacing and widefield single molecule localization microscopy (SMLM). **(e)** Improvement of the SpinFlux CRLB over SMLM as a function of the emitter-focus x-distance for varying pinhole spacing. **(f)** Average amount of signal photons after compensation for non-maximum illumination intensity as a function of the emitter-focus x-distance, for SpinFlux with varying pinhole spacing and widefield single molecule localization microscopy (SMLM). **(g)** Average amount of background photons per pixel after compensation for non-maximum illumination intensity as a function of the emitter-focus x-distance, for SpinFlux with varying pinhole spacing and widefield single molecule localization microscopy (SMLM). **(h)** CRLB in x-direction as a function of expected signal photon count for varying values of the expected background photon count. The pinhole radius $r_p = 3\sigma_{PSF}$ and pinhole spacing $r = 2\sigma_{PSF}$ were used and $(x_f, y_f) = (\theta_x, \theta_y)$.

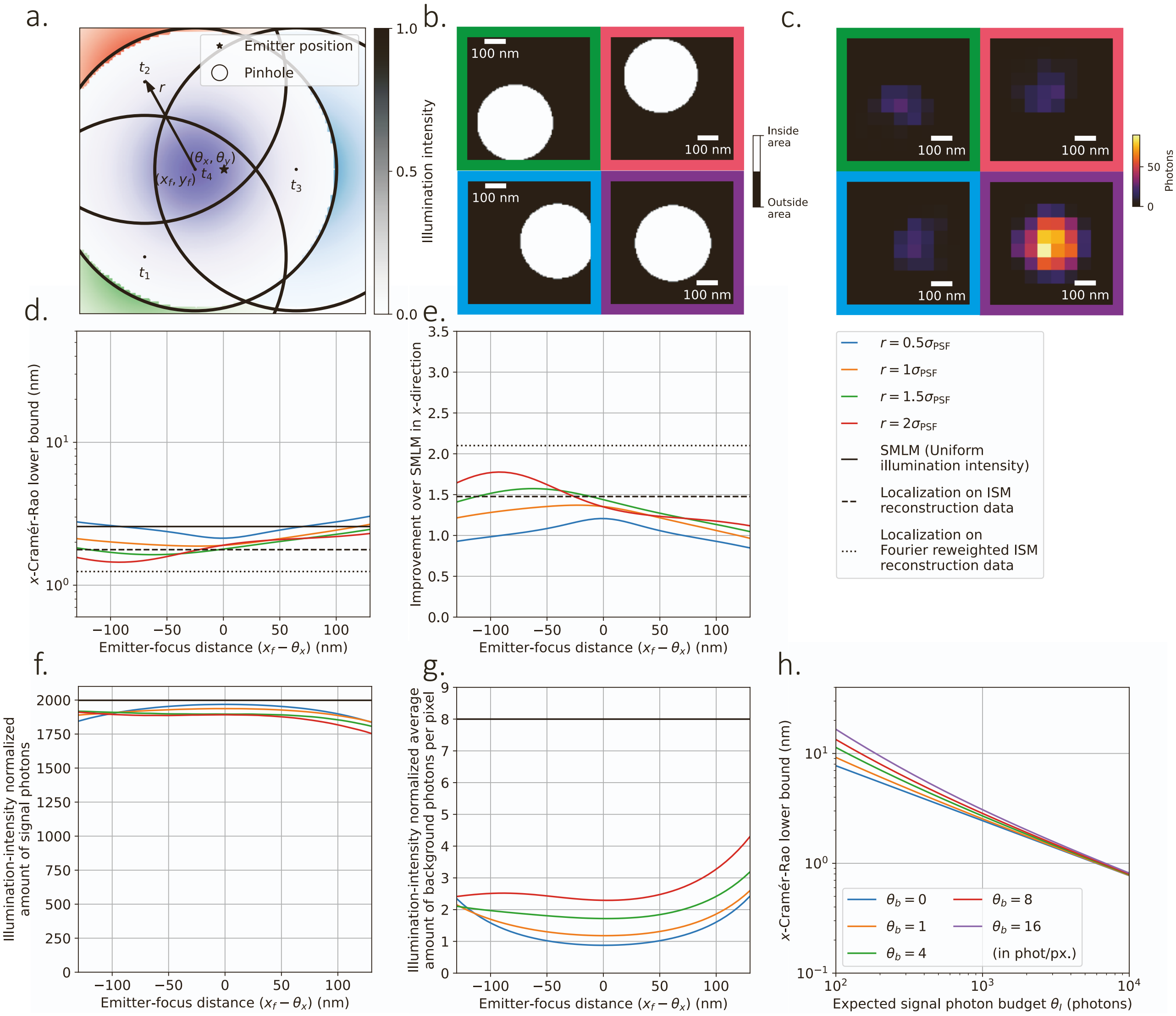

Figure S13: Theoretical minimum localization uncertainty of SpinFlux localization with four pinholes and patterns in a 90° rotated equilateral triangle configuration with a center pinhole. The pattern is rotated clockwise by 90 degrees with respect to Figure S12. In (c-g), we used 2000 expected signal photons and 8 expected background photons per pixel, with pinhole radius $r_\mathrm{p} = 3\sigma_\mathrm{PSF}$. Results are evaluated for the scenario where the entire signal photon budget is exhausted after illumination with all patterns (disregarding signal photons blocked by the spinning disk). **(a)** Schematic overview of SpinFlux localization with a triangle of three pinholes with an additional center pinhole, centered at focus coordinates $(x_\mathrm{f}, y_\mathrm{f})$. In (d-g), the x-distance $(x_\mathrm{f} - \theta_x)$ between the pattern focus and the emitter is varied, where $y_\mathrm{f} = \theta_y$. **(b)** Example of pinholes in the region of interest (650 × 650 nm). The pinhole radius $r_\mathrm{p} = 2\sigma_\mathrm{PSF}$ and pinhole spacing $r = 1.5\sigma_\mathrm{PSF}$ were used. The pinhole masks were discretized with $N_{\mathrm{M},x}$, $N_{\mathrm{M},y} = 100$ mesh pixels in each direction. **(c)** Example of fluorescent response in the region of interest, resulting from illumination and emission through each pinhole in (b). **(d)** Cramér-Rao lower bound (CRLB) in x-direction as a function of the emitter-focus x-distance. Simulations show SpinFlux with varying pinhole spacing and widefield single molecule localization microscopy (SMLM). **(e)** Improvement of the SpinFlux CRLB over SMLM as a function of the emitter-focus x-distance for varying pinhole spacing. **(f)** Average amount of signal photons after compensation for non-maximum illumination intensity as a function of the emitter-focus x-distance, for SpinFlux with varying pinhole spacing and widefield single molecule localization microscopy (SMLM). **(g)** Average amount of background photons per pixel after compensation for non-maximum illumination intensity as a function of the emitter-focus x-distance, for SpinFlux with varying pinhole spacing and widefield single molecule localization microscopy (SMLM). **(h)** CRLB in x-direction as a function of expected signal photon count for varying values of the expected background photon count. The pinhole radius $r_\mathrm{p} = 3\sigma_\mathrm{PSF}$ and pinhole spacing $r = 2\sigma_\mathrm{PSF}$ were used and $(x_\mathrm{f}, y_\mathrm{f}) = (\theta_x, \theta_y)$.

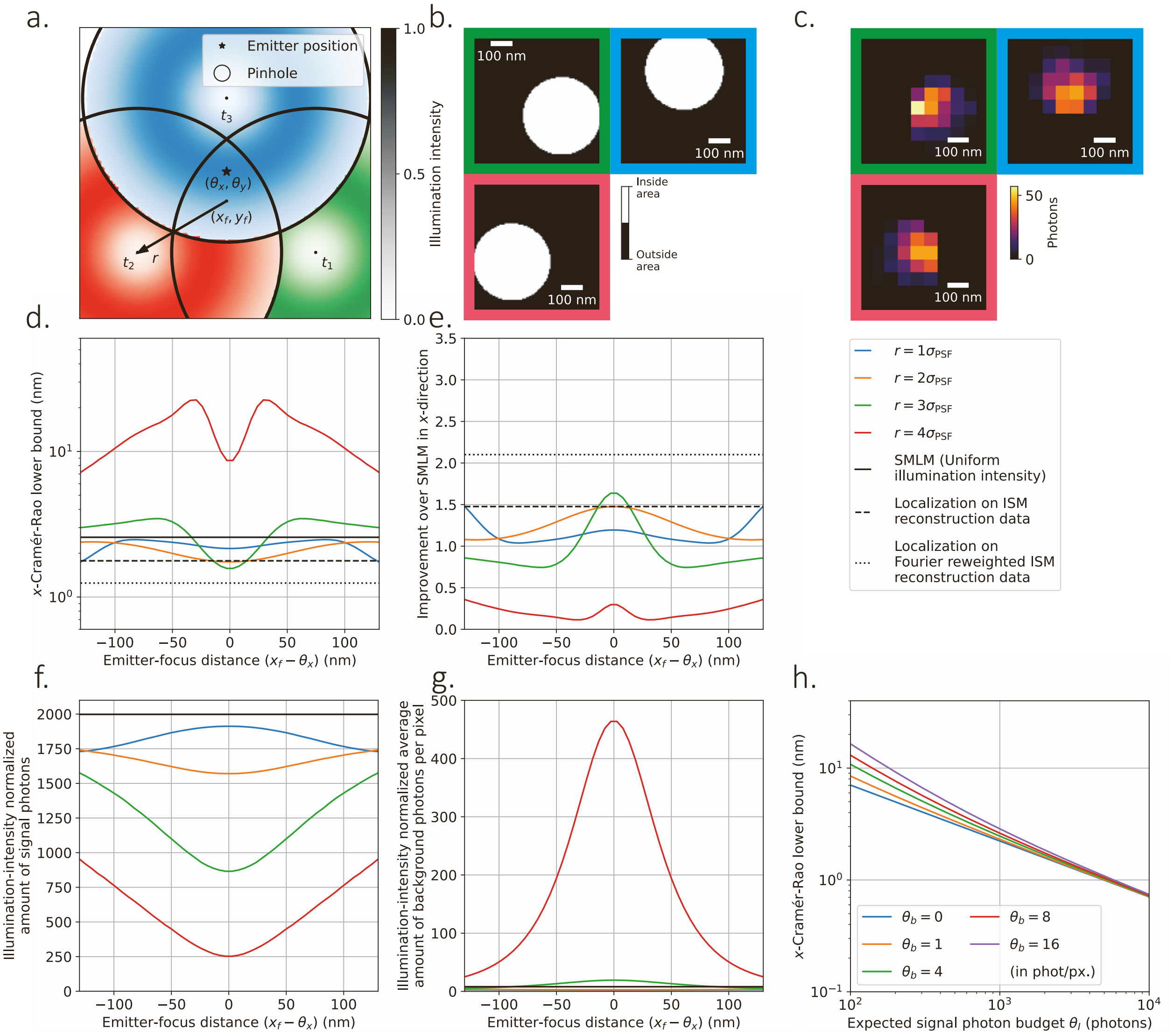

Figure S14: Theoretical minimum localization uncertainty of SpinFlux localization with three pinholes and donut-shaped patterns in an equilateral triangle configuration. In (c-g), we used 2000 expected signal photons and 8 expected background photons per pixel, with pinhole radius $r_p = 3\sigma_{PSF}$. Results are evaluated for the scenario where the entire signal photon budget is exhausted after illumination with all patterns (disregarding signal photons blocked by the spinning disk). **(a)** Schematic overview of SpinFlux localization with a triangle of three pinholes, centered at focus coordinates (x_f, y_f). In (d-g), the x-distance $(x_f - \theta_x)$ between the pattern focus and the emitter is varied, where $y_f = \theta_y$. **(b)** Example of pinholes in the region of interest (650×650 nm). The pinhole radius $r_p = 2\sigma_{PSF}$ and pinhole spacing $r = 1.5\sigma_{PSF}$ were used. The pinhole masks were discretized with $N_{M,x}, N_{M,y} = 100$ mesh pixels in each direction. **(c)** Example of fluorescent response in the region of interest, resulting from illumination and emission through each pinhole in (b). **(d)** Cramér-Rao lower bound (CRLB) in x-direction as a function of the emitter-focus x-distance. Simulations show SpinFlux with varying pinhole spacing and widefield single molecule localization microscopy (SMLM). **(e)** Improvement of the SpinFlux CRLB over SMLM as a function of the emitter-focus x-distance for varying pinhole spacing. **(f)** Average amount of signal photons after compensation for non-maximum illumination intensity as a function of the emitter-focus x-distance, for SpinFlux with varying pinhole spacing and widefield single molecule localization microscopy (SMLM). **(g)** Average amount of background photons per pixel after compensation for non-maximum illumination intensity as a function of the emitter-focus x-distance, for SpinFlux with varying pinhole spacing and widefield single molecule localization microscopy (SMLM). **(h)** CRLB in x-direction as a function of expected signal photon count for varying values of the expected background photon count. The pinhole radius $r_p = 3\sigma_{PSF}$ and pinhole spacing $r = 2\sigma_{PSF}$ were used and $(x_f, y_f) = (\theta_x, \theta_y)$.

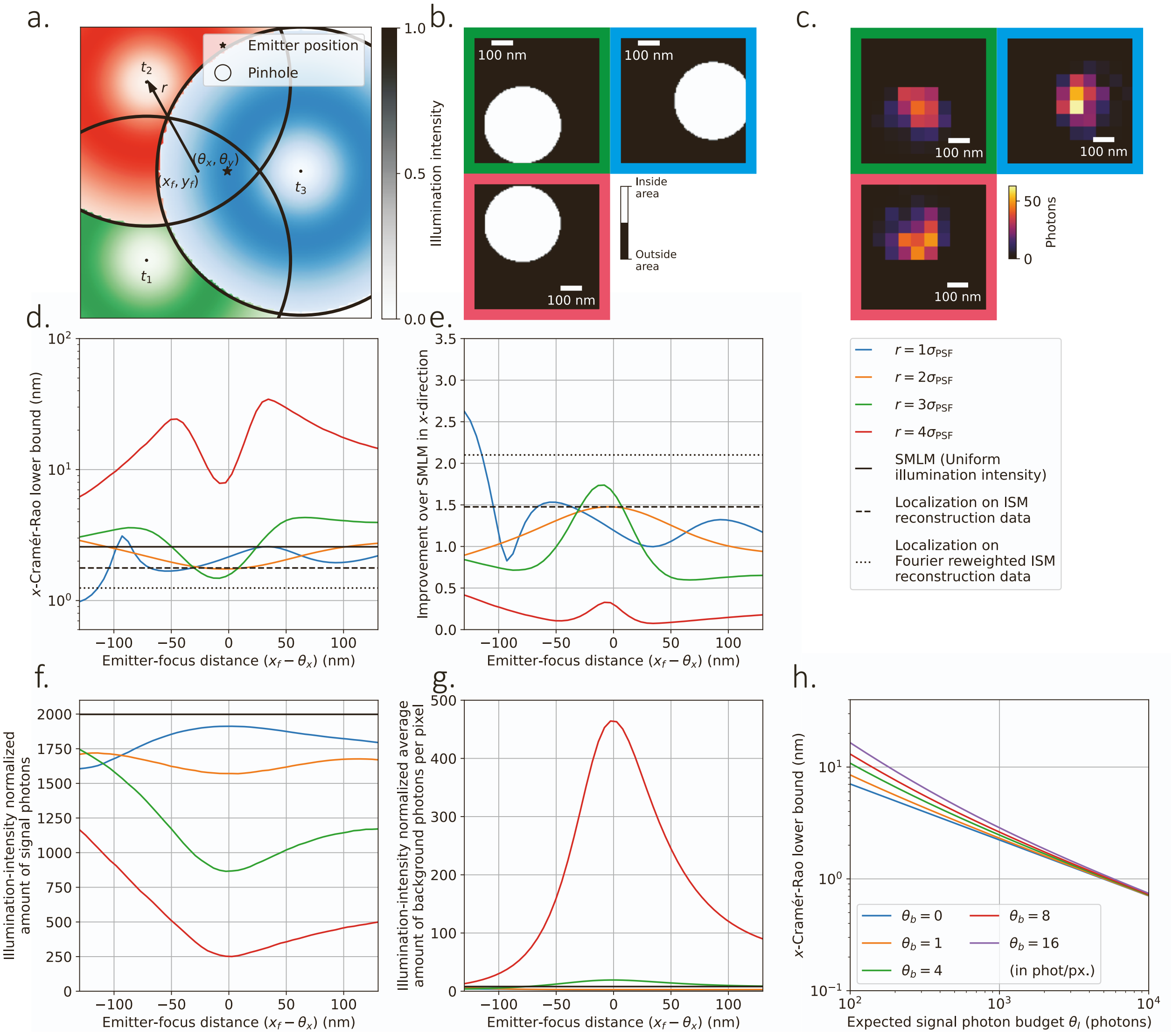

Figure S15: Theoretical minimum localization uncertainty of SpinFlux localization with three pinholes and donut-shaped patterns in a 90° rotated equilateral triangle configuration. The pattern is rotated clockwise by 90 degrees with respect to Figure 4 of the main text. In (c-g), we used 2000 expected signal photons and 8 expected background photons per pixel, with pinhole radius $r_p = 3\sigma_{PSF}$. Results are evaluated for the scenario where the entire signal photon budget is exhausted after illumination with all patterns (disregarding signal photons blocked by the spinning disk). **(a)** Schematic overview of SpinFlux localization with a triangle of three pinholes, centered at focus coordinates (x_f, y_f). In (d-g), the x-distance $(x_f - \theta_x)$ between the pattern focus and the emitter is varied, where $y_f = \theta_y$. **(b)** Example of pinholes in the region of interest (650 × 650 nm). The pinhole radius $r_p = 2\sigma_{PSF}$ and pinhole spacing $r = 1.5\sigma_{PSF}$ were used. The pinhole masks were discretized with $N_{M,x}$, $N_{M,y} = 100$ mesh pixels in each direction. **(c)** Example of fluorescent response in the region of interest, resulting from illumination and emission through each pinhole in (b). **(d)** Cramér-Rao lower bound (CRLB) in x-direction as a function of the emitter-focus x-distance. Simulations show SpinFlux with varying pinhole spacing and widefield single molecule localization microscopy (SMLM). **(e)** Improvement of the SpinFlux CRLB over SMLM as a function of the emitter-focus x-distance for varying pinhole spacing. **(f)** Average amount of signal photons after compensation for non-maximum illumination intensity as a function of the emitter-focus x-distance, for SpinFlux with varying pinhole spacing and widefield single molecule localization microscopy (SMLM). **(g)** Average amount of background photons per pixel after compensation for non-maximum illumination intensity as a function of the emitter-focus x-distance, for SpinFlux with varying pinhole spacing and widefield single molecule localization microscopy (SMLM). **(h)** CRLB in x-direction as a function of expected signal photon count for varying values of the expected background photon count. The pinhole radius $r_p = 3\sigma_{PSF}$ and pinhole spacing $r = 2\sigma_{PSF}$ were used and $(x_f, y_f) = (\theta_x, \theta_y)$.

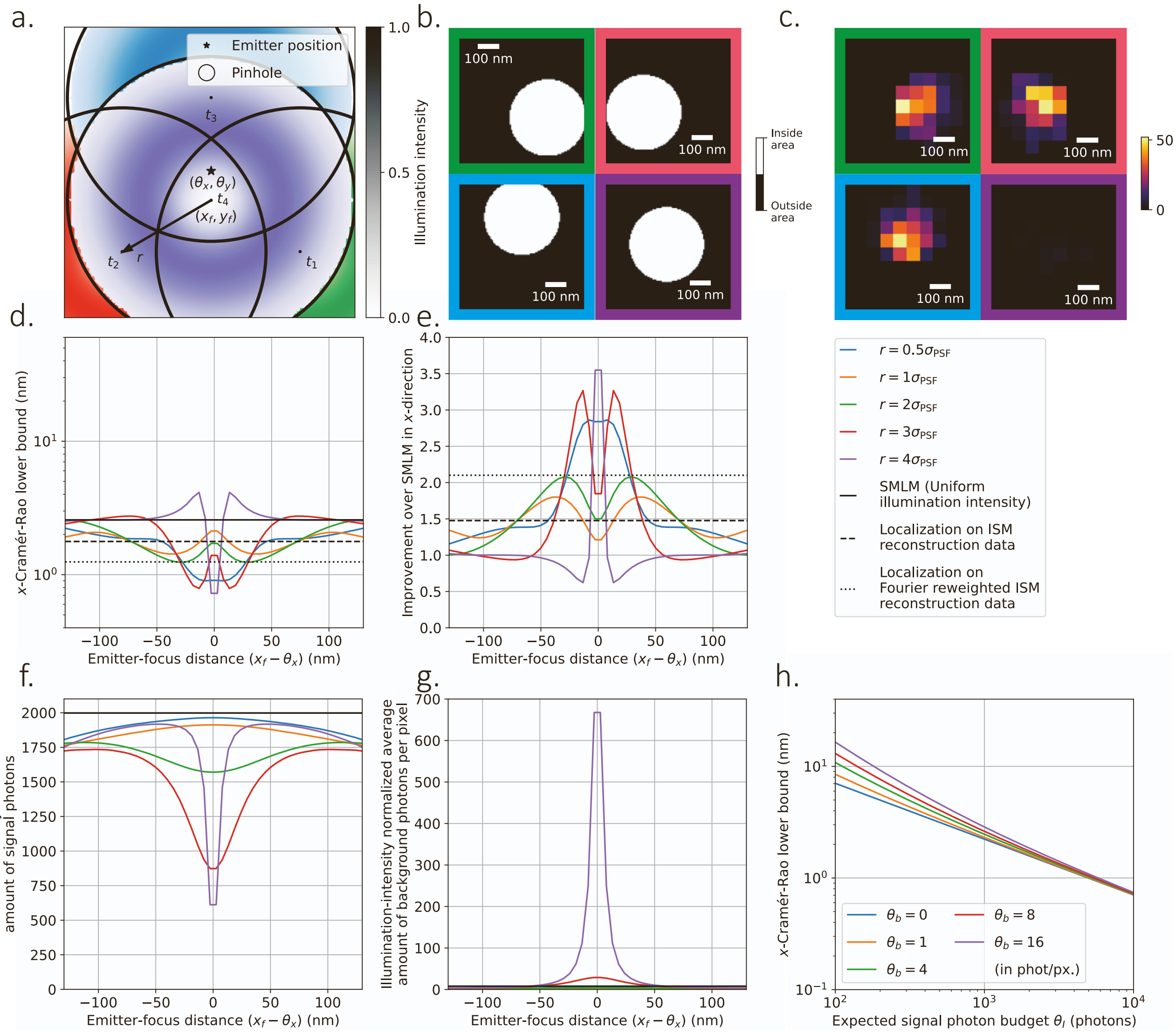

Figure S16: Theoretical minimum localization uncertainty of SpinFlux localization with four pinholes and donut-shaped patterns in an equilateral triangle configuration with a center pinhole. In (c-g), we used 2000 expected signal photons and 8 expected background photons per pixel, with pinhole radius $r_p = 3\sigma_{PSF}$. Results are evaluated for the scenario where the entire signal photon budget is exhausted after illumination with all patterns (disregarding signal photons blocked by the spinning disk). **(a)** Schematic overview of SpinFlux localization with a triangle of three pinholes with an additional center pinhole, centered at focus coordinates (x_f, y_f). In (d-g), the x-distance $(x_f - \theta_x)$ between the pattern focus and the emitter is varied, where $y_f = \theta_y$. **(b)** Example of pinholes in the region of interest (650 × 650 nm). The pinhole radius $r_p = 2\sigma_{PSF}$ and pinhole spacing $r = 1.5\sigma_{PSF}$ were used. The pinhole masks were discretized with $N_{M,x}$, $N_{M,y} = 100$ mesh pixels in each direction. **(c)** Example of fluorescent response in the region of interest, resulting from illumination and emission through each pinhole in (b). **(d)** Cramér-Rao lower bound (CRLB) in x-direction as a function of the emitter-focus x-distance. Simulations show SpinFlux with varying pinhole spacing and widefield single molecule localization microscopy (SMLM). **(e)** Improvement of the SpinFlux CRLB over SMLM as a function of the emitter-focus x-distance for varying pinhole spacing. **(f)** Average amount of signal photons after compensation for non-maximum illumination intensity as a function of the emitter-focus x-distance, for SpinFlux with varying pinhole spacing and widefield single molecule localization microscopy (SMLM). **(g)** Average amount of background photons per pixel after compensation for non-maximum illumination intensity as a function of the emitter-focus x-distance, for SpinFlux with varying pinhole spacing and widefield single molecule localization microscopy (SMLM). **(h)** CRLB in x-direction as a function of expected signal photon count for varying values of the expected background photon count. The pinhole radius $r_p = 3\sigma_{PSF}$ and pinhole spacing $r = 2\sigma_{PSF}$ were used and $(x_f, y_f) = (\theta_x, \theta_y)$.

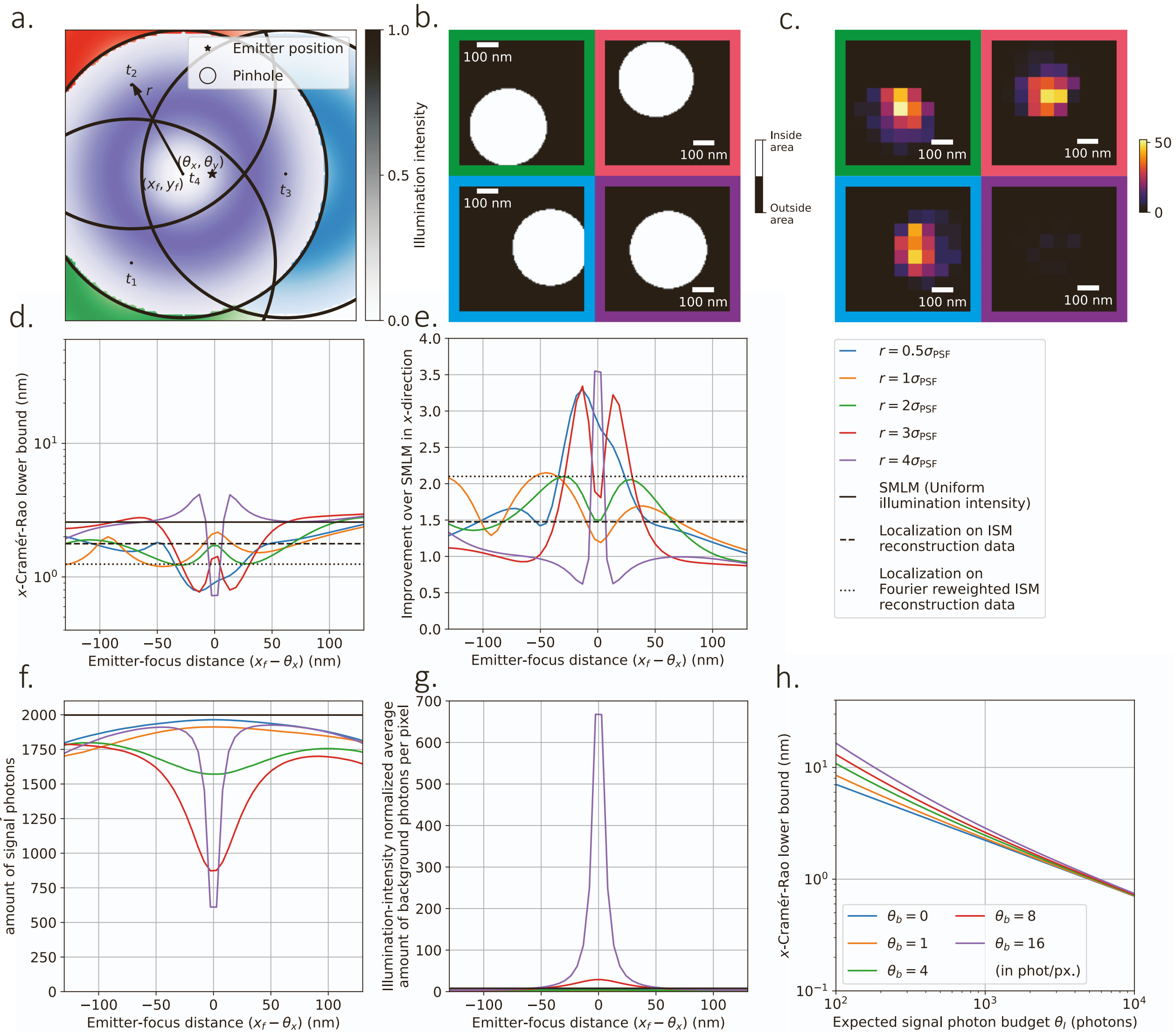

Figure S17: Theoretical minimum localization uncertainty of SpinFlux localization with four pinholes and donut-shaped patterns in a 90° rotated equilateral triangle configuration with a center pinhole. The pattern is rotated clockwise by 90 degrees with respect to Figure S12. In (c-g), we used 2000 expected signal photons and 8 expected background photons per pixel, with pinhole radius $r_p = 3\sigma_{PSF}$. Results are evaluated for the scenario where the entire signal photon budget is exhausted after illumination with all patterns (disregarding signal photons blocked by the spinning disk). **(a)** Schematic overview of SpinFlux localization with a triangle of three pinholes with an additional center pinhole, centered at focus coordinates (x_f, y_f). In (d-g), the x-distance $(x_f - \theta_x)$ between the pattern focus and the emitter is varied, where $y_f = \theta_y$. **(b)** Example of pinholes in the region of interest (650 × 650 nm). The pinhole radius $r_p = 2\sigma_{PSF}$ and pinhole spacing $r = 1.5\sigma_{PSF}$ were used. The pinhole masks were discretized with $N_{M,x}$, $N_{M,y} = 100$ mesh pixels in each direction. **(c)** Example of fluorescent response in the region of interest, resulting from illumination and emission through each pinhole in (b). **(d)** Cramér-Rao lower bound (CRLB) in x-direction as a function of the emitter-focus x-distance. Simulations show SpinFlux with varying pinhole spacing and widefield single molecule localization microscopy (SMLM). **(e)** Improvement of the SpinFlux CRLB over SMLM as a function of the emitter-focus x-distance for varying pinhole spacing. **(f)** Average amount of signal photons after compensation for non-maximum illumination intensity as a function of the emitter-focus x-distance, for SpinFlux with varying pinhole spacing and widefield single molecule localization microscopy (SMLM). **(g)** Average amount of background photons per pixel after compensation for non-maximum illumination intensity as a function of the emitter-focus x-distance, for SpinFlux with varying pinhole spacing and widefield single molecule localization microscopy (SMLM). **(h)** CRLB in x-direction as a function of expected signal photon count for varying values of the expected background photon count. The pinhole radius $r_p = 3\sigma_{PSF}$ and pinhole spacing $r = 2\sigma_{PSF}$ were used and $(x_f, y_f) = (\theta_x, \theta_y)$.

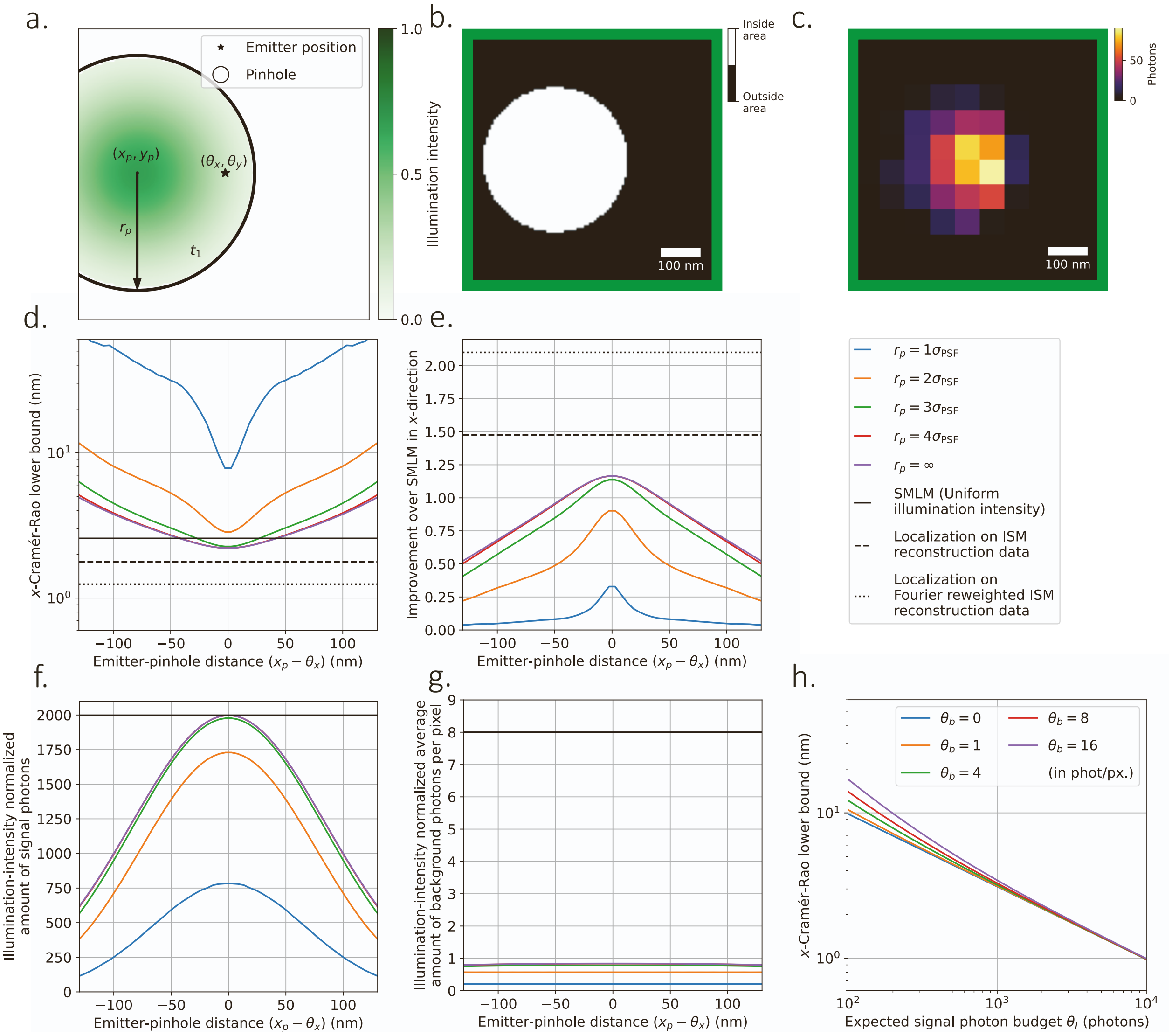

Figure S18: Theoretical minimum localization uncertainty of SpinFlux localization with one x-offset pinhole and pattern. In (c-g), 2000 expected signal photons and 8 expected background photons per pixel were used. Results are evaluated for the scenario where the illumination power and time are constant during illumination with this pattern. **(a)** Schematic overview of SpinFlux localization with one pinhole with radius r_p, centered at coordinates $(x_\mathrm{p}, y_\mathrm{p})$. In (d-g), the x-distance $(x_\mathrm{p} - \theta_x)$ between the pinhole and the emitter is varied, where $y_\mathrm{p} = \theta_y$. **(b)** Example of pinhole in the region of interest (650 × 650 nm). The pinhole radius $r_\mathrm{p} = 2\sigma_\mathrm{PSF}$ was used. The pinhole mask was discretized with $N_{\mathrm{M},x}$, $N_{\mathrm{M},y}$ = 100 mesh pixels in each direction. **(c)** Example of fluorescent response in the region of interest, resulting from illumination and emission through the pinhole in (b). **(d)** Cramér-Rao lower bound (CRLB) in x-direction as a function of the emitter-pinhole x-distance. Simulations show SpinFlux with varying pinhole sizes and widefield single-molecule localization microscopy (SMLM). **(e)** Improvement of the SpinFlux CRLB over SMLM as a function of the emitter-pinhole x-distance for varying pinhole sizes. **(f)** Average amount of signal photons after compensation for non-maximum illumination intensity as a function of the emitter-pinhole x-distance, for SpinFlux with varying pinhole sizes and widefield single molecule localization microscopy (SMLM). **(g)** Average amount of background photons per pixel after compensation for non-maximum illumination intensity as a function of the emitter-pinhole x-distance, for SpinFlux with varying pinhole sizes and widefield single molecule localization microscopy (SMLM). **(h)** CRLB in x-direction as a function of the expected signal photon count for varying values of the expected background photon count. The pinhole radius $r_\mathrm{p} = 3\sigma_\mathrm{PSF}$ was used and $(x_\mathrm{p}, y_\mathrm{p}) = (\theta_x, \theta_y)$.

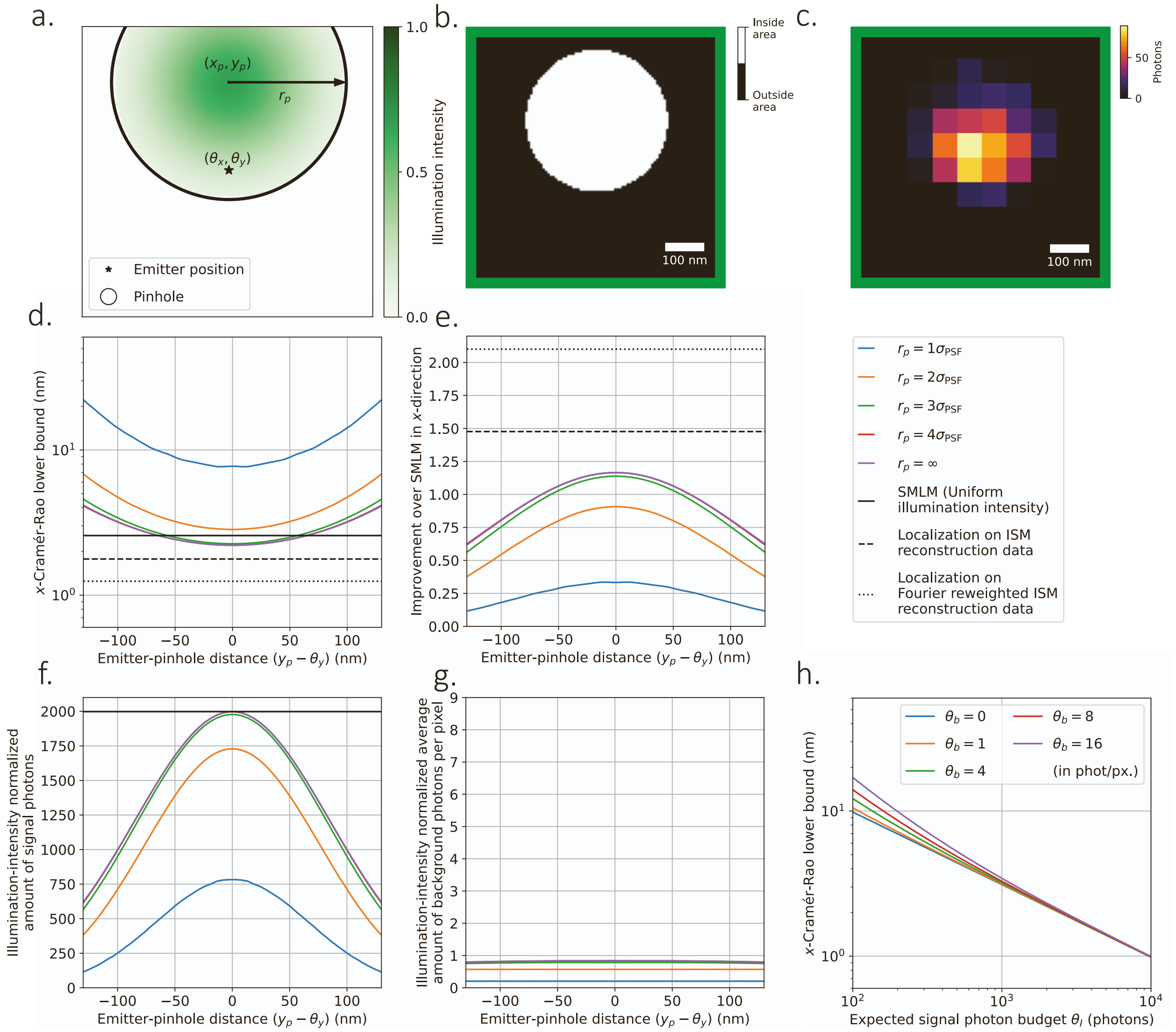

Figure S19: Theoretical minimum localization uncertainty of SpinFlux localization with one y-offset pinhole and pattern. In (c-g), 2000 expected signal photons and 8 expected background photons per pixel were used. Results are evaluated for the scenario where the illumination power and time are constant during illumination with this pattern. **(a)** Schematic overview of SpinFlux localization with one pinhole with radius r_{p}, centered at coordinates $(x_{\mathrm{p}}, y_{\mathrm{p}})$. In (d-g), the y-distance $(y_{\mathrm{p}} - \theta_y)$ between the pinhole and the emitter is varied, where $x_{\mathrm{p}} = \theta_x$. **(b)** Example of pinhole in the region of interest (650 × 650 nm). The pinhole radius $r_{\mathrm{p}} = 2\sigma_{\mathrm{PSF}}$ was used. The pinhole mask was discretized with $N_{\mathrm{M},x}$, $N_{\mathrm{M},y} = 100$ mesh pixels in each direction. **(c)** Example of fluorescent response in the region of interest, resulting from illumination and emission through the pinhole in (b). **(d)** Cramér-Rao lower bound (CRLB) in x-direction as a function of the emitter-pinhole y-distance. Simulations show SpinFlux with varying pinhole sizes and widefield single-molecule localization microscopy (SMLM). **(e)** Improvement of the SpinFlux CRLB over SMLM as a function of the emitter-pinhole y-distance for varying pinhole sizes. **(f)** Average amount of signal photons after compensation for non-maximum illumination intensity as a function of the emitter-pinhole y-distance, for SpinFlux with varying pinhole sizes and widefield single molecule localization microscopy (SMLM). **(g)** Average amount of background photons per pixel after compensation for non-maximum illumination intensity as a function of the emitter-pinhole y-distance, for SpinFlux with varying pinhole sizes and widefield single molecule localization microscopy (SMLM). **(h)** CRLB in x-direction as a function of the expected signal photon count for varying values of the expected background photon count. The pinhole radius $r_{\mathrm{p}} = 3\sigma_{\mathrm{PSF}}$ was used and $(x_{\mathrm{p}}, y_{\mathrm{p}}) = (\theta_x, \theta_y)$.

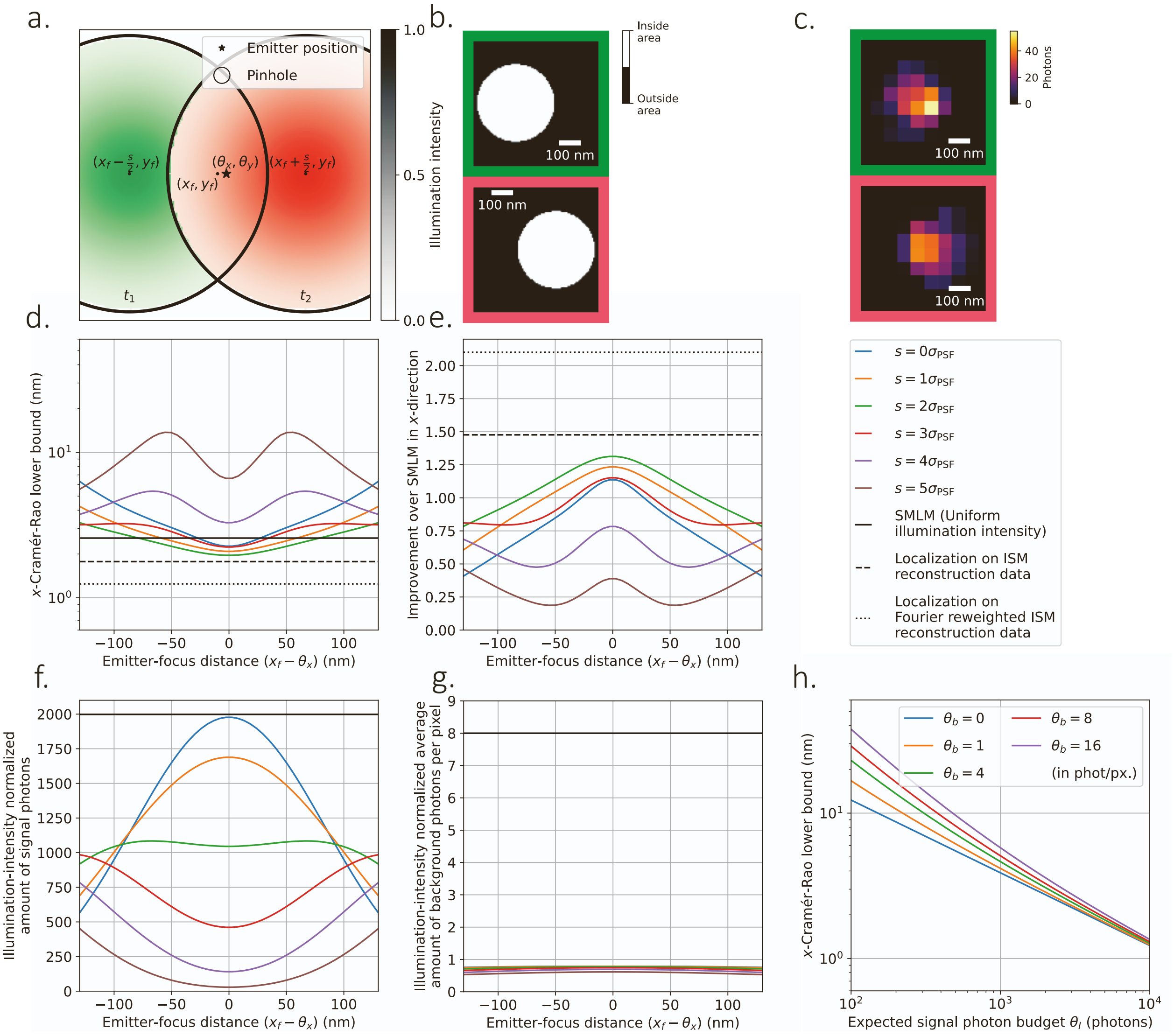

Figure S20: Theoretical minimum localization uncertainty of SpinFlux localization with two pinholes and patterns separated in the x-direction. In (c-g), 2000 expected signal photons and 8 expected background photons per pixel were used, with pinhole radius $r_p = 3\sigma_{PSF}$. Results are evaluated for the scenario where the illumination power and time are constant during illumination with all patterns. **(a)** Schematic overview of SpinFlux localization with two pinholes, separated in x and centered around the focus coordinates (x_f, y_f). In (d-g), the x-distance $(x_f - \theta_x)$ between the pattern focus and the emitter is varied, where $y_f = \theta_y$. **(b)** Example of pinholes in the region of interest (650 × 650 nm). The pinhole radius $r_p = 2\sigma_{PSF}$ and pinhole separation $s = 2\sigma_{PSF}$ were used. The pinhole masks were discretized with $N_{M,x}, N_{M,y} = 100$ mesh pixels in each direction. **(c)** Example of fluorescent response in the region of interest, resulting from illumination and emission through each pinhole in (b). **(d)** Cramér-Rao lower bound (CRLB) in x-direction as a function of the emitter-focus x-distance. Simulations show SpinFlux with varying pinhole separations and widefield single molecule localization microscopy (SMLM). **(e)** Improvement of the SpinFlux CRLB over SMLM as a function of the emitter-focus x-distance for varying pinhole separations. **(f)** Average amount of signal photons after compensation for non-maximum illumination intensity as a function of the emitter-focus x-distance, for SpinFlux with varying pinhole separations and widefield single molecule localization microscopy (SMLM). **(g)** Average amount of background photons per pixel after compensation for non-maximum illumination intensity as a function of the emitter-focus x-distance, for SpinFlux with varying pinhole separations and widefield single molecule localization microscopy (SMLM). **(h)** CRLB in x-direction as a function of expected signal photon count for varying values of the expected background photon count. The pinhole radius $r_p = 3\sigma_{PSF}$ and pinhole separation $s = 4\sigma_{PSF}$ were used and $(x_f, y_f) = (\theta_x, \theta_y)$.

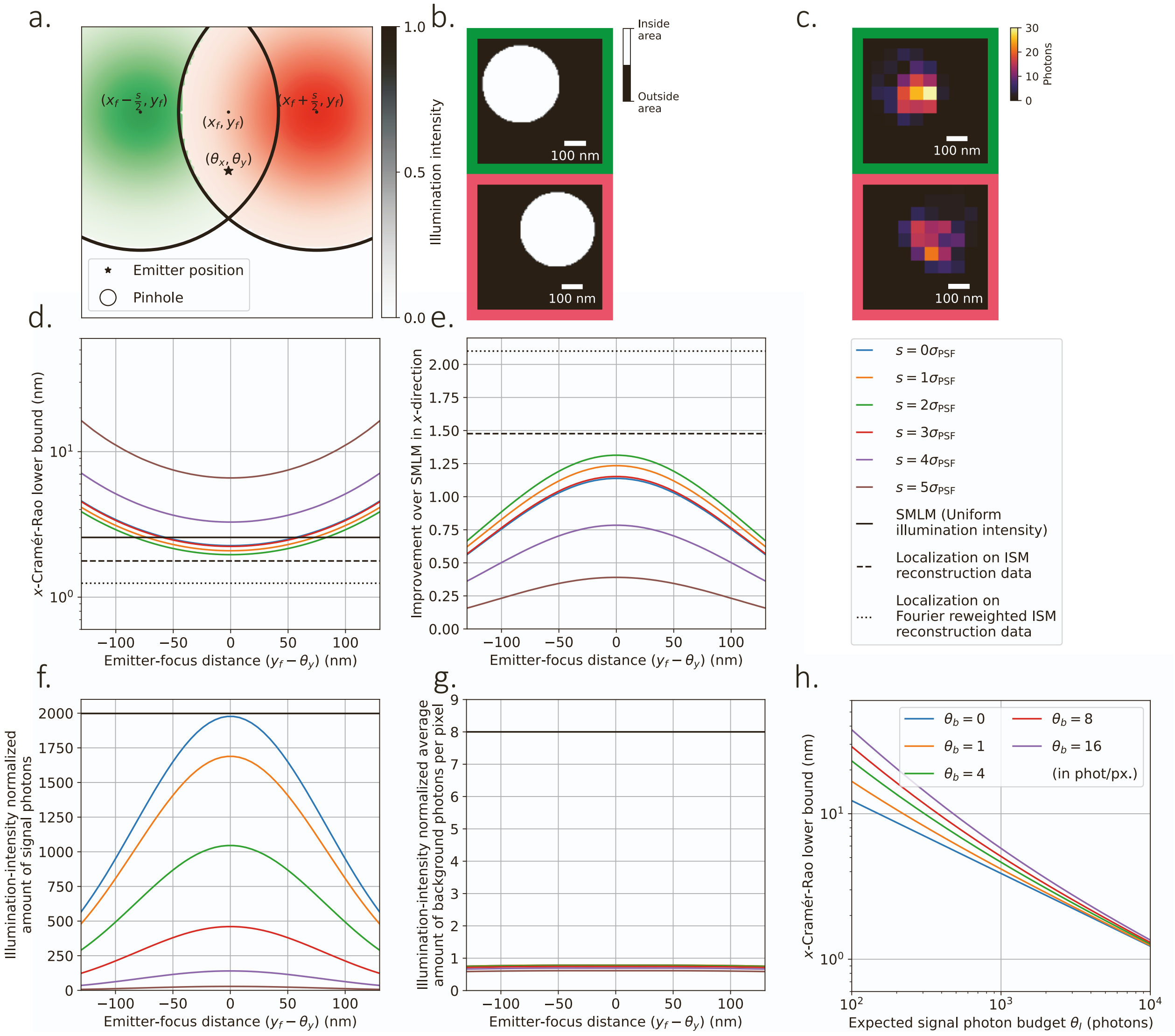

Figure S21: Theoretical minimum localization uncertainty of SpinFlux localization with two y-offset pinholes and patterns separated in the x-direction. In (c-g), 2000 expected signal photons and 8 expected background photons per pixel were used, with pinhole radius $r_\mathrm{p} = 3\sigma_\mathrm{PSF}$. Results are evaluated for the scenario where the illumination power and time are constant during illumination with all patterns. **(a)** Schematic overview of SpinFlux localization with two pinholes, separated in x and centered around the focus coordinates $(x_\mathrm{f}, y_\mathrm{f})$. In (d-g), the y-distance $(y_\mathrm{f} - \theta_y)$ between the pattern focus and the emitter is varied, where $x_\mathrm{f} = \theta_x$. **(b)** Example of pinholes in the region of interest (650 × 650 nm). The pinhole radius $r_\mathrm{p} = 2\sigma_\mathrm{PSF}$ and pinhole separation $s = 2\sigma_\mathrm{PSF}$ were used. The pinhole masks were discretized with $N_{\mathrm{M},x}$, $N_{\mathrm{M},y} = 100$ mesh pixels in each direction. **(c)** Example of fluorescent response in the region of interest, resulting from illumination and emission through each pinhole in (b). **(d)** Cramér-Rao lower bound (CRLB) in x-direction as a function of the emitter-focus y-distance. Simulations show SpinFlux with varying pinhole separations and widefield single molecule localization microscopy (SMLM). **(e)** Improvement of the SpinFlux CRLB over SMLM as a function of the emitter-focus y-distance for varying pinhole separations. **(f)** Average amount of signal photons after compensation for non-maximum illumination intensity as a function of the emitter-focus y-distance, for SpinFlux with varying pinhole separations and widefield single molecule localization microscopy (SMLM). **(g)** Average amount of background photons per pixel after compensation for non-maximum illumination intensity as a function of the emitter-focus y-distance, for SpinFlux with varying pinhole separations and widefield single molecule localization microscopy (SMLM). **(h)** CRLB in x-direction as a function of expected signal photon count for varying values of the expected background photon count. The pinhole radius $r_\mathrm{p} = 3\sigma_\mathrm{PSF}$ and pinhole separation $s = 4\sigma_\mathrm{PSF}$ were used and $(x_\mathrm{f}, y_\mathrm{f}) = (\theta_x, \theta_y)$.

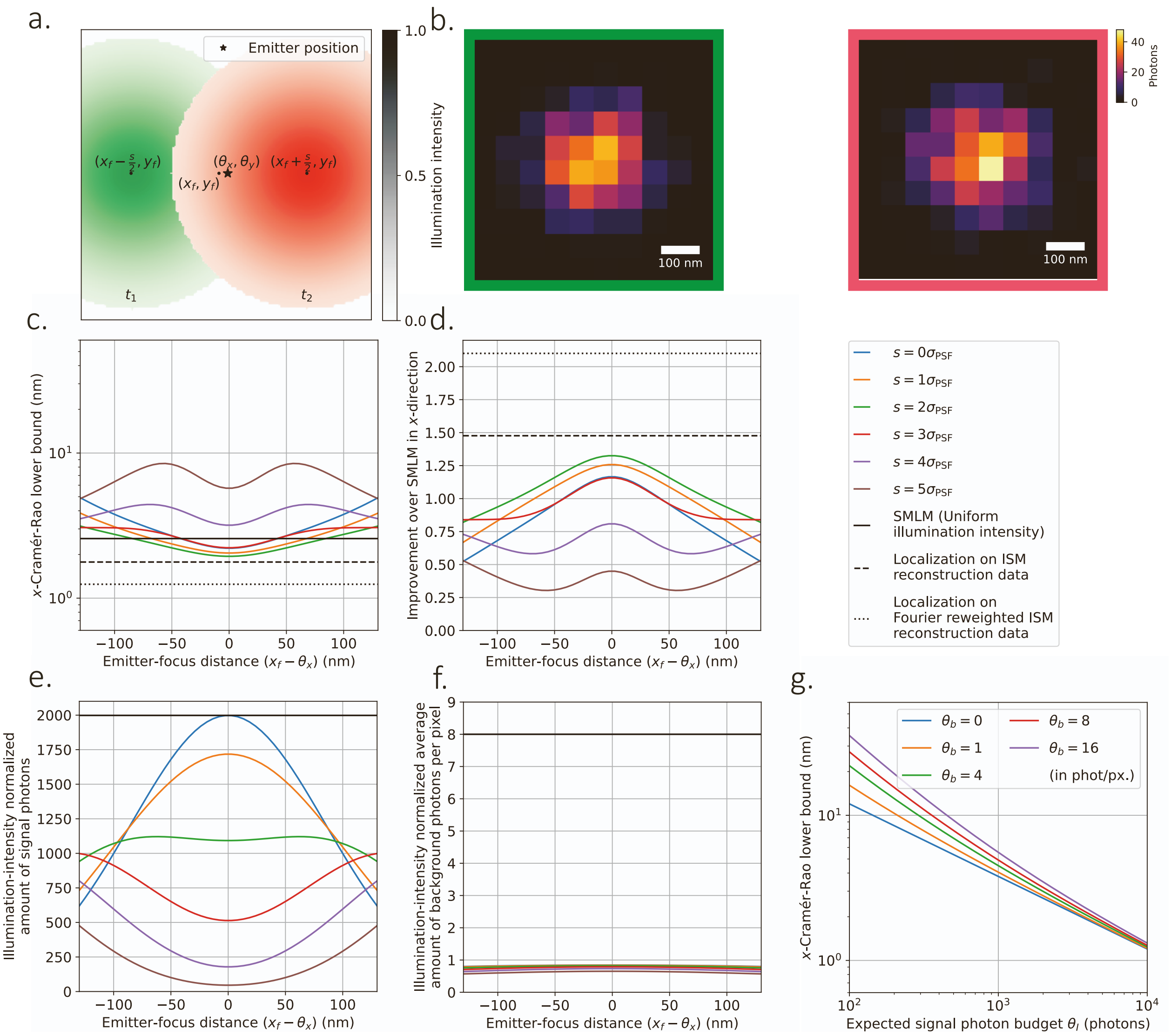

Figure S22: Theoretical minimum localization uncertainty of SpinFlux localization with two patterns without pinholes separated in the x-direction. In (b-f), 2000 expected signal photons and 8 expected background photons per pixel were used. Results are evaluated for the scenario where the illumination power and time are constant during illumination with all patterns. **(a)** Schematic overview of SpinFlux localization with two pinholes, separated in x and centered around the focus coordinates (x_f, y_f). In (c-f), the x-distance $(x_\text{f} - \theta_x)$ between the pattern focus and the emitter is varied, where $y_\text{f} = \theta_y$. **(b)** Example of fluorescent response in the region of interest, resulting from illumination and emission by each pattern in (a). **(c)** Cramér-Rao lower bound (CRLB) in x-direction as a function of the emitter-focus x-distance. Simulations show SpinFlux with varying pinhole separations and widefield single molecule localization microscopy (SMLM). **(d)** Improvement of the SpinFlux CRLB over SMLM as a function of the emitter-focus x-distance for varying pinhole separations. **(e)** Average amount of signal photons after compensation for non-maximum illumination intensity as a function of the emitter-focus x-distance, for SpinFlux with varying pinhole separations and widefield single molecule localization microscopy (SMLM). **(f)** Average amount of background photons per pixel after compensation for non-maximum illumination intensity as a function of the emitter-focus x-distance, for SpinFlux with varying pinhole separations and widefield single molecule localization microscopy (SMLM). **(g)** CRLB in x-direction as a function of expected signal photon count for varying values of the expected background photon count. The pattern separation $s = 4\sigma_\text{PSF}$ was used and $(x_\text{f}, y_\text{f}) = (\theta_x, \theta_y)$.

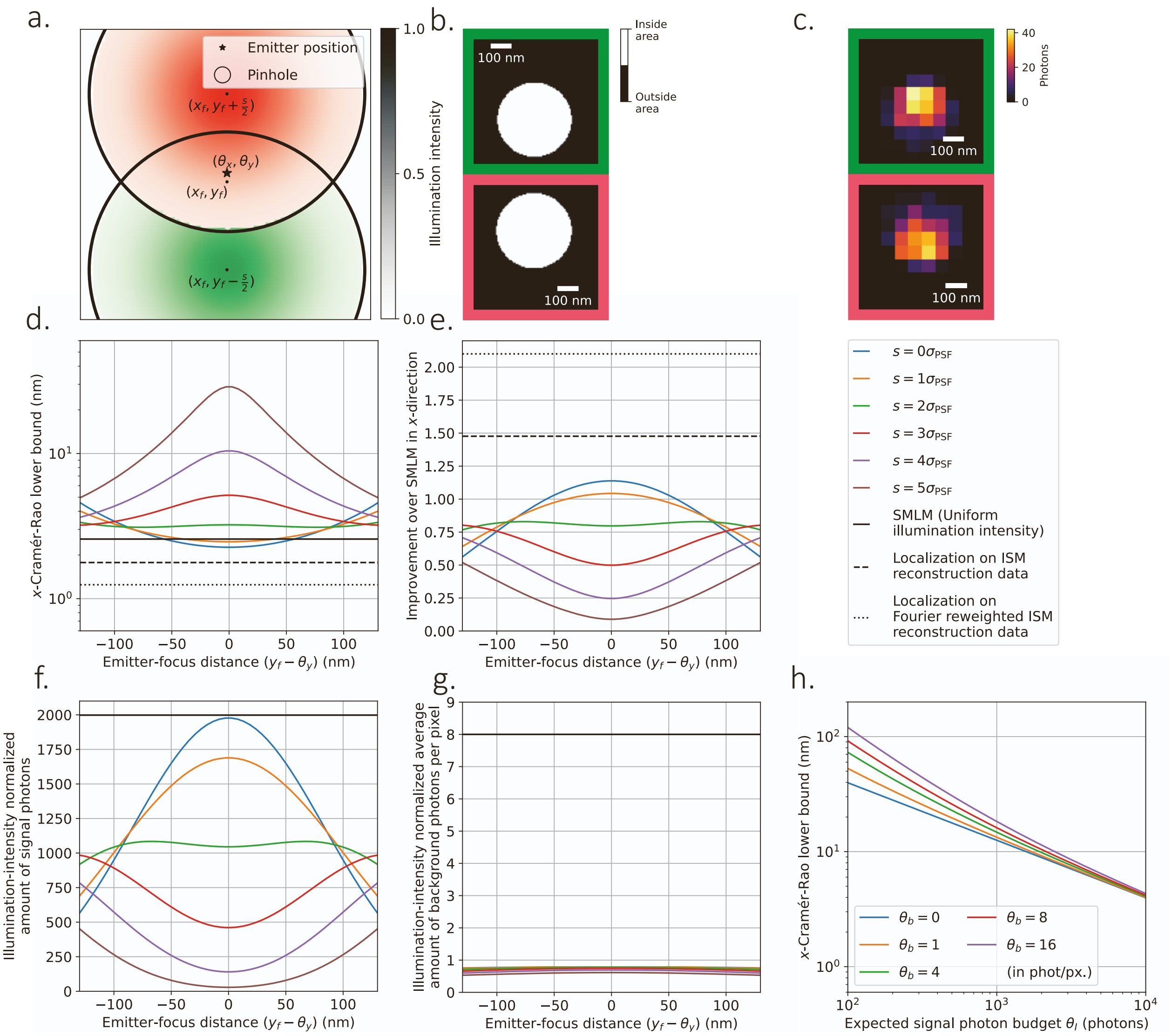

Figure S23: Theoretical minimum localization uncertainty of SpinFlux localization with two pinholes and patterns separated in the y-direction. In (c-g), 2000 expected signal photons and 8 expected background photons per pixel were used, with pinhole radius $r_p = 3\sigma_{PSF}$. Results are evaluated for the scenario where the illumination power and time are constant during illumination with all patterns. **(a)** Schematic overview of SpinFlux localization with two pinholes, separated in y and centered around the focus coordinates (x_f, y_f). In (d-g), the y-distance $(y_f - \theta_y)$ between the pattern focus and the emitter is varied, where $x_f = \theta_x$. **(b)** Example of pinholes in the region of interest (650×650 nm). The pinhole radius $r_p = 2\sigma_{PSF}$ and pinhole separation $s = 2\sigma_{PSF}$ were used. The pinhole masks were discretized with $N_{M,x}, N_{M,y} = 100$ mesh pixels in each direction. **(c)** Example of fluorescent response in the region of interest, resulting from illumination and emission through each pinhole in (b). **(d)** Cramér-Rao lower bound (CRLB) in x-direction as a function of the emitter-focus y-distance. Simulations show SpinFlux with varying pinhole separations and widefield single molecule localization microscopy (SMLM). **(e)** Improvement of the SpinFlux CRLB over SMLM as a function of the emitter-focus y-distance for varying pinhole separations. **(f)** Average amount of signal photons after compensation for non-maximum illumination intensity as a function of the emitter-focus y-distance, for SpinFlux with varying pinhole separations and widefield single molecule localization microscopy (SMLM). **(g)** Average amount of background photons per pixel after compensation for non-maximum illumination intensity as a function of the emitter-focus y-distance, for SpinFlux with varying pinhole separations and widefield single molecule localization microscopy (SMLM). **(h)** CRLB in x-direction as a function of expected signal photon count for varying values of the expected background photon count. The pinhole radius $r_p = 3\sigma_{PSF}$ and pinhole separation $s = 4\sigma_{PSF}$ were used and $(x_f, y_f) = (\theta_x, \theta_y)$.

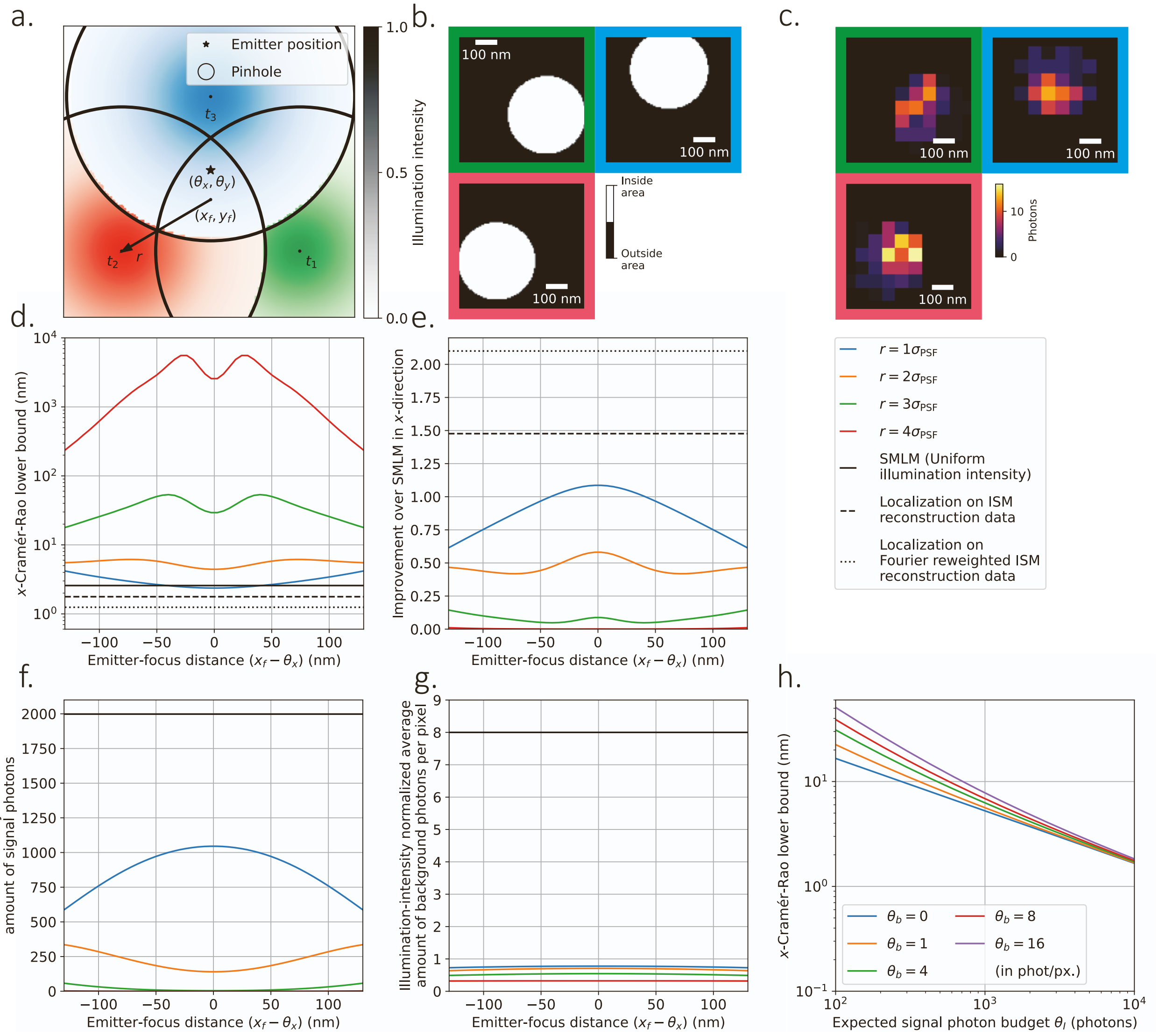

Figure S24: Theoretical minimum localization uncertainty of SpinFlux localization with three pinholes and patterns in an equilateral triangle configuration. In (c-g), we used 2000 expected signal photons and 8 expected background photons per pixel, with pinhole radius $r_p = 3\sigma_{PSF}$. Results are evaluated for the scenario where the illumination power and time are constant during illumination with all patterns. **(a)** Schematic overview of SpinFlux localization with a triangle of three pinholes, centered at focus coordinates (x_f, y_f). In (d-g), the x-distance $(x_f - \theta_x)$ between the pattern focus and the emitter is varied, where $y_f = \theta_y$. **(b)** Example of pinholes in the region of interest (650 × 650 nm). The pinhole radius $r_p = 2\sigma_{PSF}$ and pinhole spacing $r = 1.5\sigma_{PSF}$ were used. The pinhole masks were discretized with $N_{M,x}$, $N_{M,y} = 100$ mesh pixels in each direction. **(c)** Example of fluorescent response in the region of interest, resulting from illumination and emission through each pinhole in (b). **(d)** Cramér-Rao lower bound (CRLB) in x-direction as a function of the emitter-focus x-distance. Simulations show SpinFlux with varying pinhole spacing and widefield single molecule localization microscopy (SMLM). **(e)** Improvement of the SpinFlux CRLB over SMLM as a function of the emitter-focus x-distance for varying pinhole spacing. **(f)** Average amount of signal photons after compensation for non-maximum illumination intensity as a function of the emitter-focus x-distance, for SpinFlux with varying pinhole spacing and widefield single molecule localization microscopy (SMLM). **(g)** Average amount of background photons per pixel after compensation for non-maximum illumination intensity as a function of the emitter-focus x-distance, for SpinFlux with varying pinhole spacing and widefield single molecule localization microscopy (SMLM). **(h)** CRLB in x-direction as a function of expected signal photon count for varying values of the expected background photon count. The pinhole radius $r_p = 3\sigma_{PSF}$ and pinhole spacing $r = 2\sigma_{PSF}$ were used and $(x_f, y_f) = (\theta_x, \theta_y)$.

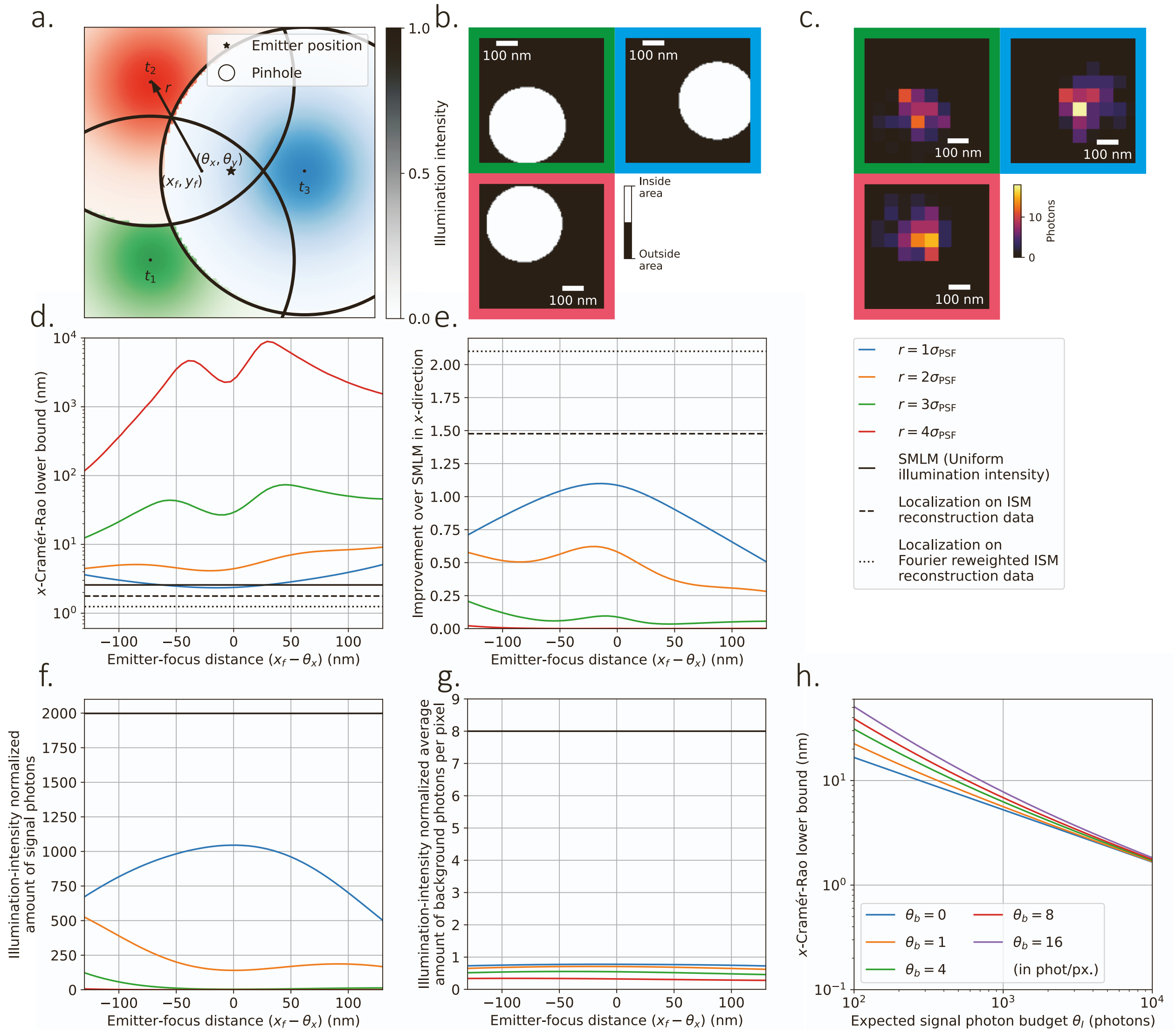

Figure S25: Theoretical minimum localization uncertainty of SpinFlux localization with three pinholes and patterns in a 90° rotated equilateral triangle configuration. The pattern is rotated clockwise by 90 degrees with respect to Figure S24. In (c-g), we used 2000 expected signal photons and 8 expected background photons per pixel, with pinhole radius $r_p = 3\sigma_{PSF}$. Results are evaluated for the scenario where the illumination power and time are constant during illumination with all patterns. **(a)** Schematic overview of SpinFlux localization with a triangle of three pinholes, centered at focus coordinates (x_f, y_f). In (d-g), the x-distance $(x_f - \theta_x)$ between the pattern focus and the emitter is varied, where $y_f = \theta_y$. **(b)** Example of pinholes in the region of interest (650 × 650 nm). The pinhole radius $r_p = 2\sigma_{PSF}$ and pinhole spacing $r = 1.5\sigma_{PSF}$ were used. The pinhole masks were discretized with $N_{M,x}$, $N_{M,y}$ = 100 mesh pixels in each direction. **(c)** Example of fluorescent response in the region of interest, resulting from illumination and emission through each pinhole in (b). **(d)** Cramér-Rao lower bound (CRLB) in x-direction as a function of the emitter-focus x-distance. Simulations show SpinFlux with varying pinhole spacing and widefield single molecule localization microscopy (SMLM). **(e)** Improvement of the SpinFlux CRLB over SMLM as a function of the emitter-focus x-distance for varying pinhole spacing. **(f)** Average amount of signal photons after compensation for non-maximum illumination intensity as a function of the emitter-focus x-distance, for SpinFlux with varying pinhole spacing and widefield single molecule localization microscopy (SMLM). **(g)** Average amount of background photons per pixel after compensation for non-maximum illumination intensity as a function of the emitter-focus x-distance, for SpinFlux with varying pinhole spacing and widefield single molecule localization microscopy (SMLM). **(h)** CRLB in x-direction as a function of expected signal photon count for varying values of the expected background photon count. The pinhole radius $r_p = 3\sigma_{PSF}$ and pinhole spacing $r = 2\sigma_{PSF}$ were used and $(x_f, y_f) = (\theta_x, \theta_y)$.

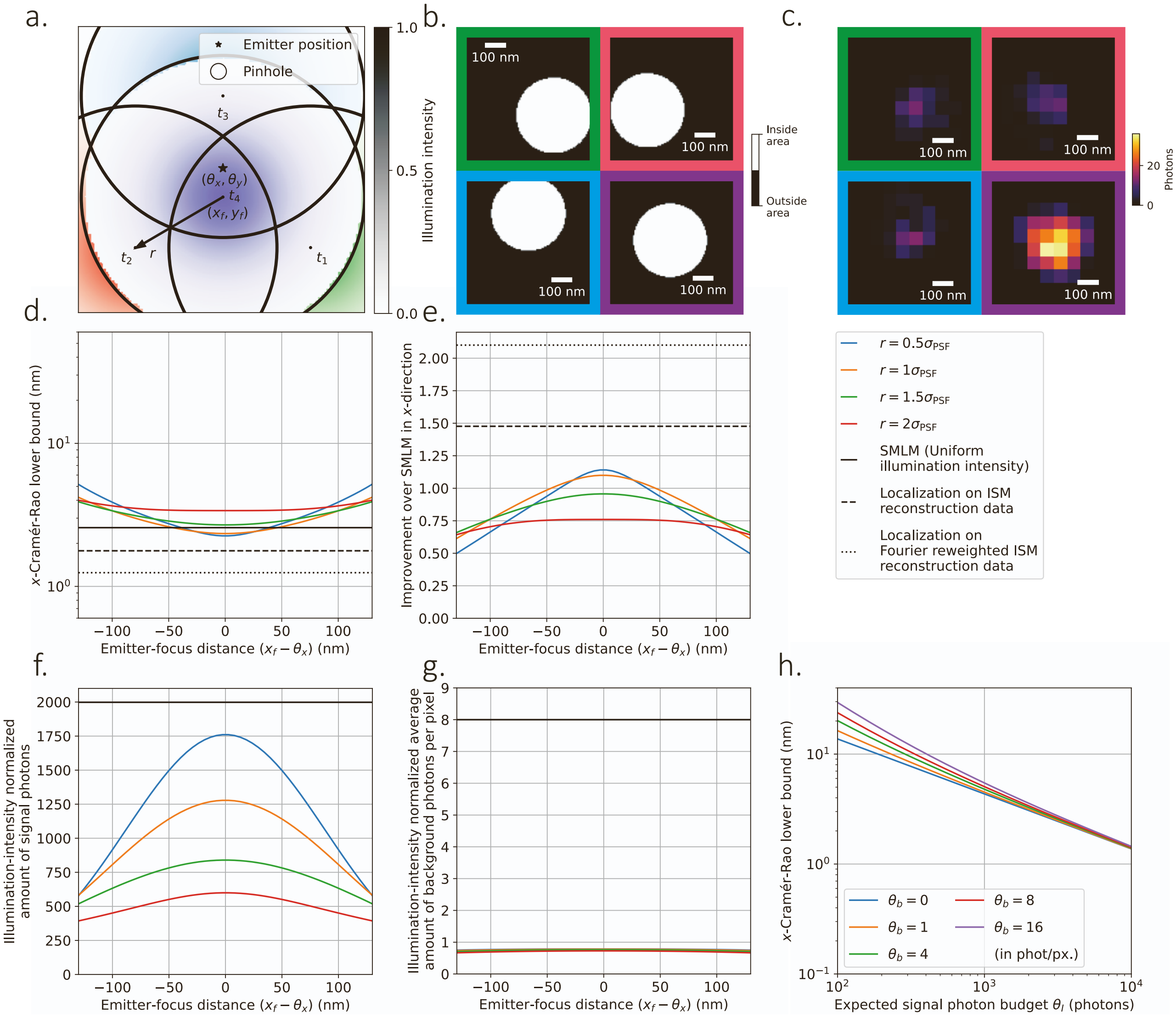

Figure S26: Theoretical minimum localization uncertainty of SpinFlux localization with four pinholes and patterns in an equilateral triangle configuration with a center pinhole. In (c-g), we used 2000 expected signal photons and 8 expected background photons per pixel, with pinhole radius $r_p = 3\sigma_{PSF}$. Results are evaluated for the scenario where the illumination power and time are constant during illumination with all patterns. **(a)** Schematic overview of SpinFlux localization with a triangle of three pinholes with an additional center pinhole, centered at focus coordinates (x_f, y_f). In (d-g), the x-distance $(x_f - \theta_x)$ between the pattern focus and the emitter is varied, where $y_f = \theta_y$. **(b)** Example of pinholes in the region of interest (650×650 nm). The pinhole radius $r_p = 2\sigma_{PSF}$ and pinhole spacing $r = 1.5\sigma_{PSF}$ were used. The pinhole masks were discretized with $N_{M,x}$, $N_{M,y} = 100$ mesh pixels in each direction. **(c)** Example of fluorescent response in the region of interest, resulting from illumination and emission through each pinhole in (b). **(d)** Cramér-Rao lower bound (CRLB) in x-direction as a function of the emitter-focus x-distance. Simulations show SpinFlux with varying pinhole spacing and widefield single molecule localization microscopy (SMLM). **(e)** Improvement of the SpinFlux CRLB over SMLM as a function of the emitter-focus x-distance for varying pinhole spacing. **(f)** Average amount of signal photons after compensation for non-maximum illumination intensity as a function of the emitter-focus x-distance, for SpinFlux with varying pinhole spacing and widefield single molecule localization microscopy (SMLM). **(g)** Average amount of background photons per pixel after compensation for non-maximum illumination intensity as a function of the emitter-focus x-distance, for SpinFlux with varying pinhole spacing and widefield single molecule localization microscopy (SMLM). **(h)** CRLB in x-direction as a function of expected signal photon count for varying values of the expected background photon count. The pinhole radius $r_p = 3\sigma_{PSF}$ and pinhole spacing $r = 2\sigma_{PSF}$ were used and $(x_f, y_f) = (\theta_x, \theta_y)$.

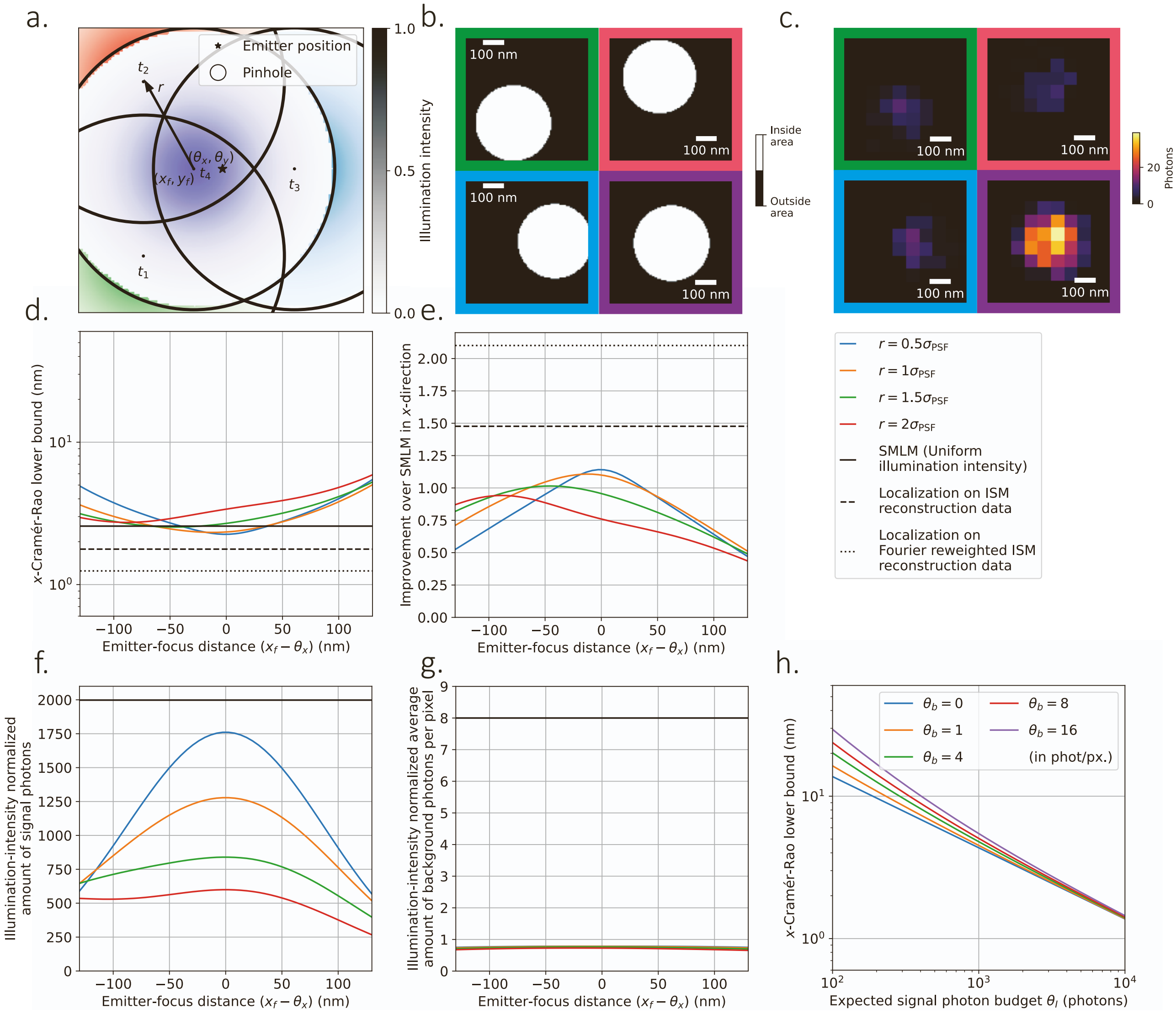

Figure S27: Theoretical minimum localization uncertainty of SpinFlux localization with four pinholes and patterns in a 90° rotated equilateral triangle configuration with a center pinhole. The pattern is rotated clockwise by 90 degrees with respect to Figure S26. In (c-g), we used 2000 expected signal photons and 8 expected background photons per pixel, with pinhole radius $r_p = 3\sigma_{PSF}$. Results are evaluated for the scenario where the illumination power and time are constant during illumination with all patterns. **(a)** Schematic overview of SpinFlux localization with a triangle of three pinholes with an additional center pinhole, centered at focus coordinates (x_f, y_f). In (d-g), the x-distance $(x_f - \theta_x)$ between the pattern focus and the emitter is varied, where $y_f = \theta_y$. **(b)** Example of pinholes in the region of interest (650 × 650 nm). The pinhole radius $r_p = 2\sigma_{PSF}$ and pinhole spacing $r = 1.5\sigma_{PSF}$ were used. The pinhole masks were discretized with $N_{M,x}$, $N_{M,y} = 100$ mesh pixels in each direction. **(c)** Example of fluorescent response in the region of interest, resulting from illumination and emission through each pinhole in (b). **(d)** Cramér-Rao lower bound (CRLB) in x-direction as a function of the emitter-focus x-distance. Simulations show SpinFlux with varying pinhole spacing and widefield single molecule localization microscopy (SMLM). **(e)** Improvement of the SpinFlux CRLB over SMLM as a function of the emitter-focus x-distance for varying pinhole spacing. **(f)** Average amount of signal photons after compensation for non-maximum illumination intensity as a function of the emitter-focus x-distance, for SpinFlux with varying pinhole spacing and widefield single molecule localization microscopy (SMLM). **(g)** Average amount of background photons per pixel after compensation for non-maximum illumination intensity as a function of the emitter-focus x-distance, for SpinFlux with varying pinhole spacing and widefield single molecule localization microscopy (SMLM). **(h)** CRLB in x-direction as a function of expected signal photon count for varying values of the expected background photon count. The pinhole radius $r_p = 3\sigma_{PSF}$ and pinhole spacing $r = 2\sigma_{PSF}$ were used and $(x_f, y_f) = (\theta_x, \theta_y)$.

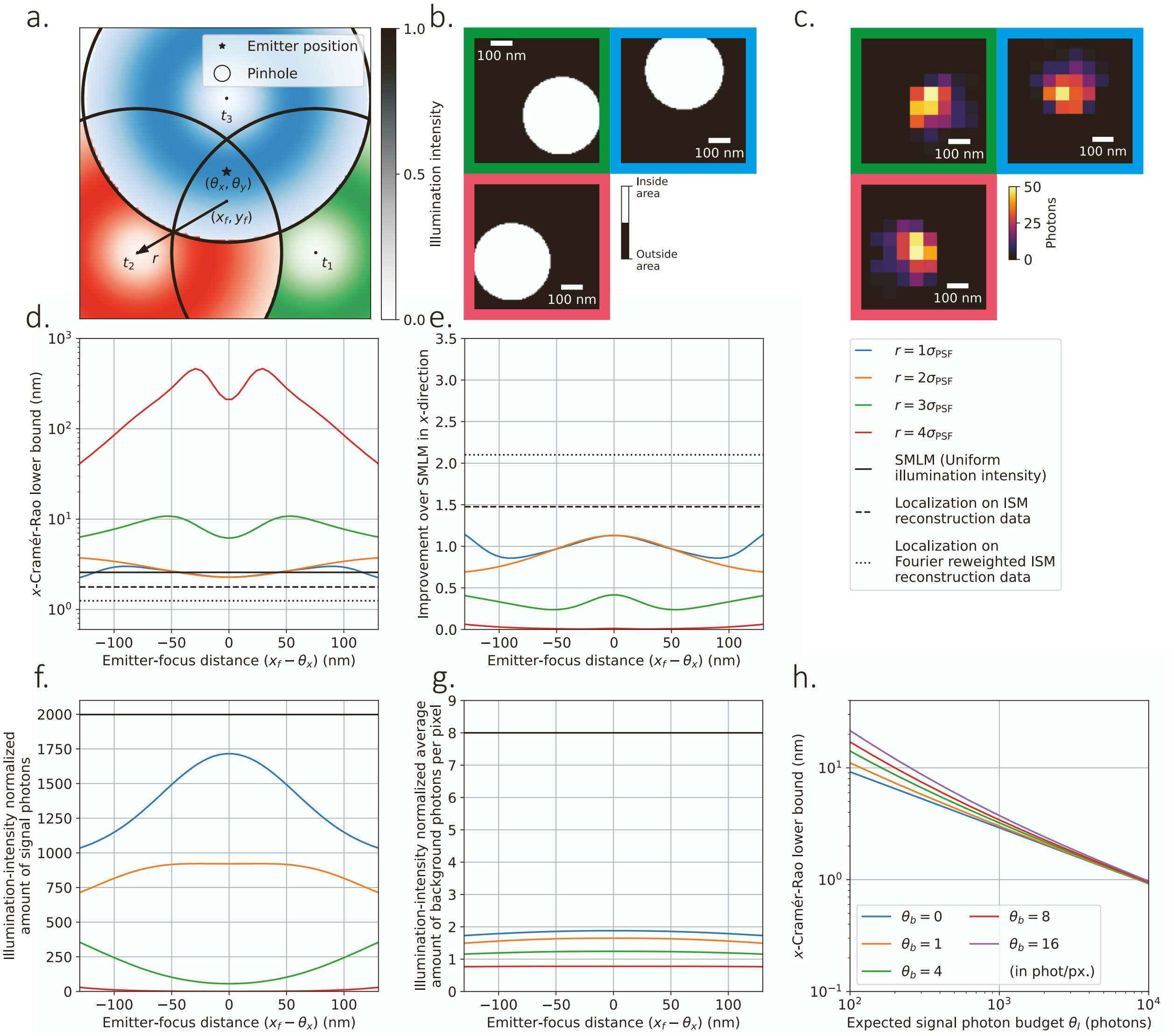

Figure S28: Theoretical minimum localization uncertainty of SpinFlux localization with three pinholes and donut-shaped patterns in an equilateral triangle configuration. In (c-g), we used 2000 expected signal photons and 8 expected background photons per pixel, with pinhole radius $r_p = 3\sigma_{PSF}$. Results are evaluated for the scenario where the illumination power and time are constant during illumination with all patterns. **(a)** Schematic overview of SpinFlux localization with a triangle of three pinholes, centered at focus coordinates (x_f, y_f). In (d-g), the x-distance $(x_f - \theta_x)$ between the pattern focus and the emitter is varied, where $y_f = \theta_y$. **(b)** Example of pinholes in the region of interest (650 × 650 nm). The pinhole radius $r_p = 2\sigma_{PSF}$ and pinhole spacing $r = 1.5\sigma_{PSF}$ were used. The pinhole masks were discretized with $N_{M,x}, N_{M,y} = 100$ mesh pixels in each direction. **(c)** Example of fluorescent response in the region of interest, resulting from illumination and emission through each pinhole in (b). **(d)** Cramér-Rao lower bound (CRLB) in x-direction as a function of the emitter-focus x-distance. Simulations show SpinFlux with varying pinhole spacing and widefield single molecule localization microscopy (SMLM). **(e)** Improvement of the SpinFlux CRLB over SMLM as a function of the emitter-focus x-distance for varying pinhole spacing. **(f)** Average amount of signal photons after compensation for non-maximum illumination intensity as a function of the emitter-focus x-distance, for SpinFlux with varying pinhole spacing and widefield single molecule localization microscopy (SMLM). **(g)** Average amount of background photons per pixel after compensation for non-maximum illumination intensity as a function of the emitter-focus x-distance, for SpinFlux with varying pinhole spacing and widefield single molecule localization microscopy (SMLM). **(h)** CRLB in x-direction as a function of expected signal photon count for varying values of the expected background photon count. The pinhole radius $r_p = 3\sigma_{PSF}$ and pinhole spacing $r = 2\sigma_{PSF}$ were used and $(x_f, y_f) = (\theta_x, \theta_y)$.

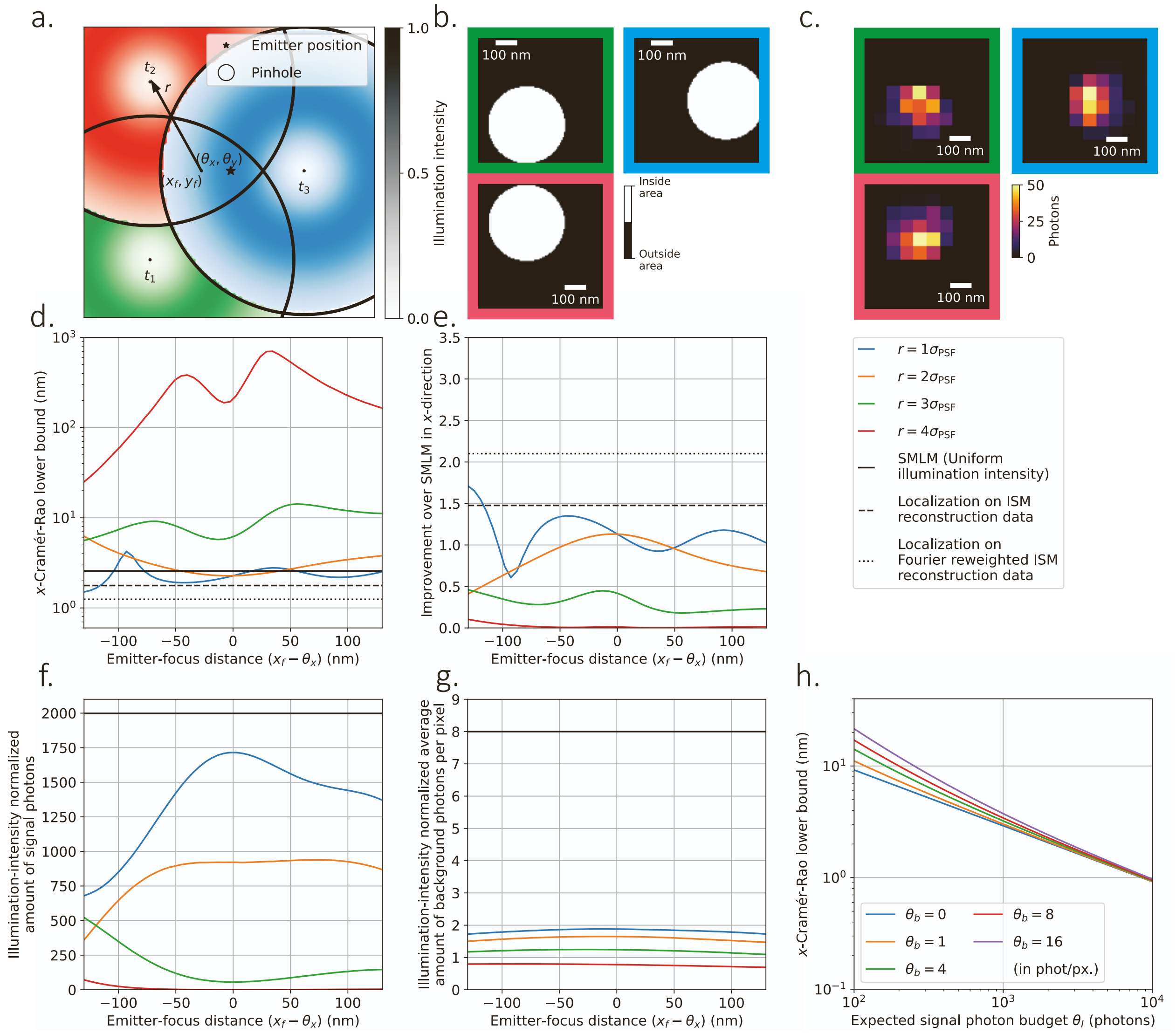

Figure S29: Theoretical minimum localization uncertainty of SpinFlux localization with three pinholes and donut-shaped patterns in a 90° rotated equilateral triangle configuration. The pattern is rotated clockwise by 90 degrees with respect to Figure S24. In (c-g), we used 2000 expected signal photons and 8 expected background photons per pixel, with pinhole radius $r_p = 3\sigma_{PSF}$. Results are evaluated for the scenario where the illumination power and time are constant during illumination with all patterns. **(a)** Schematic overview of SpinFlux localization with a triangle of three pinholes, centered at focus coordinates (x_f, y_f). In (d-g), the x-distance $(x_f - \theta_x)$ between the pattern focus and the emitter is varied, where $y_f = \theta_y$. **(b)** Example of pinholes in the region of interest (650 × 650 nm). The pinhole radius $r_p = 2\sigma_{PSF}$ and pinhole spacing $r = 1.5\sigma_{PSF}$ were used. The pinhole masks were discretized with $N_{M,x}, N_{M,y} = 100$ mesh pixels in each direction. **(c)** Example of fluorescent response in the region of interest, resulting from illumination and emission through each pinhole in (b). **(d)** Cramér-Rao lower bound (CRLB) in x-direction as a function of the emitter-focus x-distance. Simulations show SpinFlux with varying pinhole spacing and widefield single molecule localization microscopy (SMLM). **(e)** Improvement of the SpinFlux CRLB over SMLM as a function of the emitter-focus x-distance for varying pinhole spacing. **(f)** Average amount of signal photons after compensation for non-maximum illumination intensity as a function of the emitter-focus x-distance, for SpinFlux with varying pinhole spacing and widefield single molecule localization microscopy (SMLM). **(g)** Average amount of background photons per pixel after compensation for non-maximum illumination intensity as a function of the emitter-focus x-distance, for SpinFlux with varying pinhole spacing and widefield single molecule localization microscopy (SMLM). **(h)** CRLB in x-direction as a function of expected signal photon count for varying values of the expected background photon count. The pinhole radius $r_p = 3\sigma_{PSF}$ and pinhole spacing $r = 2\sigma_{PSF}$ were used and $(x_f, y_f) = (\theta_x, \theta_y)$.

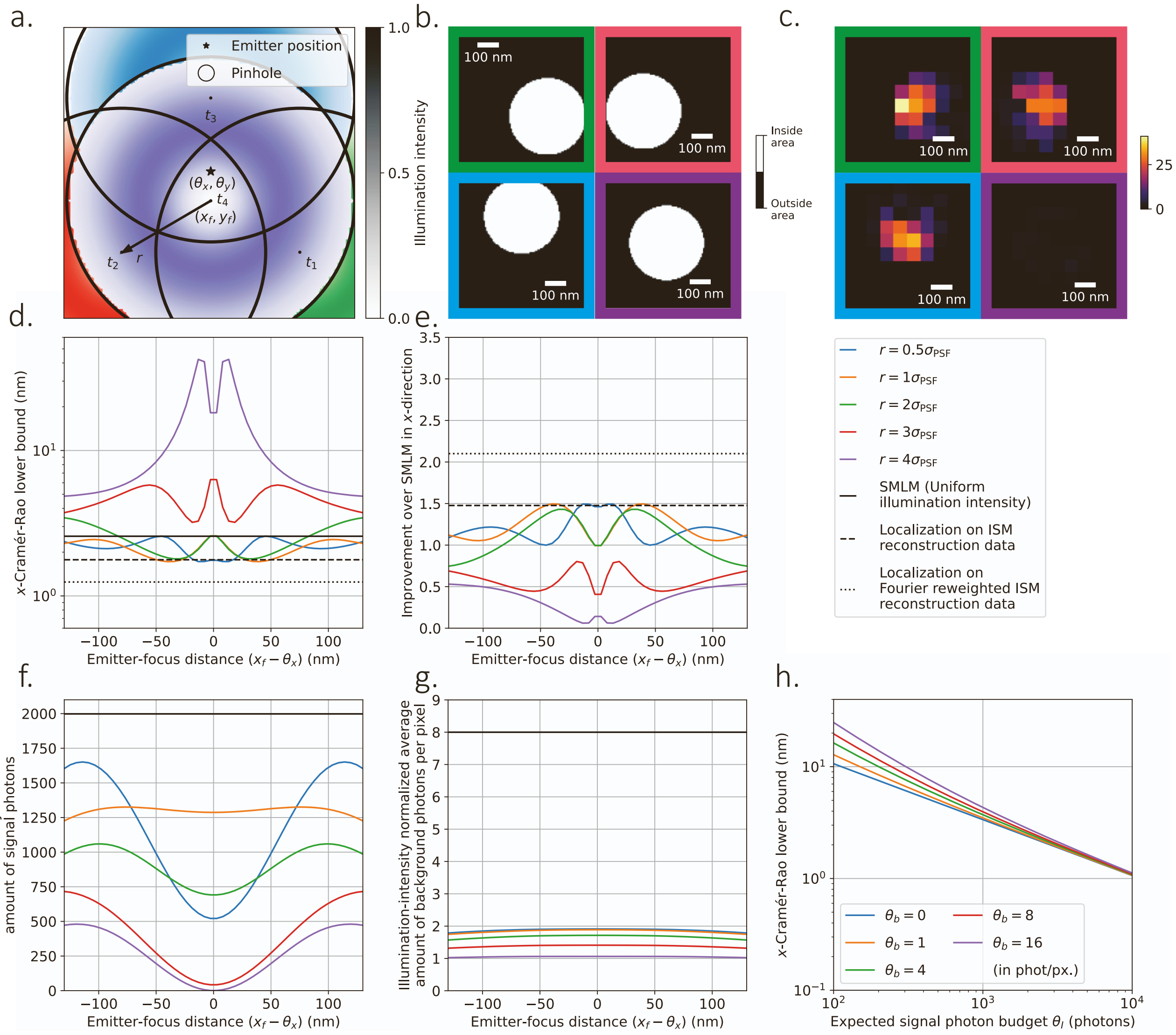

Figure S30: Theoretical minimum localization uncertainty of SpinFlux localization with four pinholes and donut-shaped patterns in an equilateral triangle configuration with a center pinhole. In (c-g), we used 2000 expected signal photons and 8 expected background photons per pixel, with pinhole radius $r_p = 3\sigma_{PSF}$. Results are evaluated for the scenario where the illumination power and time are constant during illumination with all patterns. **(a)** Schematic overview of SpinFlux localization with a triangle of three pinholes with an additional center pinhole, centered at focus coordinates (x_f, y_f). In (d-g), the x-distance $(x_f - \theta_x)$ between the pattern focus and the emitter is varied, where $y_f = \theta_y$. **(b)** Example of pinholes in the region of interest (650 × 650 nm). The pinhole radius $r_p = 2\sigma_{PSF}$ and pinhole spacing $r = 1.5\sigma_{PSF}$ were used. The pinhole masks were discretized with $N_{M,x}$, $N_{M,y} = 100$ mesh pixels in each direction. **(c)** Example of fluorescent response in the region of interest, resulting from illumination and emission through each pinhole in (b). **(d)** Cramér-Rao lower bound (CRLB) in x-direction as a function of the emitter-focus x-distance. Simulations show SpinFlux with varying pinhole spacing and widefield single molecule localization microscopy (SMLM). **(e)** Improvement of the SpinFlux CRLB over SMLM as a function of the emitter-focus x-distance for varying pinhole spacing. **(f)** Average amount of signal photons after compensation for non-maximum illumination intensity as a function of the emitter-focus x-distance, for SpinFlux with varying pinhole spacing and widefield single molecule localization microscopy (SMLM). **(g)** Average amount of background photons per pixel after compensation for non-maximum illumination intensity as a function of the emitter-focus x-distance, for SpinFlux with varying pinhole spacing and widefield single molecule localization microscopy (SMLM). **(h)** CRLB in x-direction as a function of expected signal photon count for varying values of the expected background photon count. The pinhole radius $r_p = 3\sigma_{PSF}$ and pinhole spacing $r = 2\sigma_{PSF}$ were used and $(x_f, y_f) = (\theta_x, \theta_y)$.

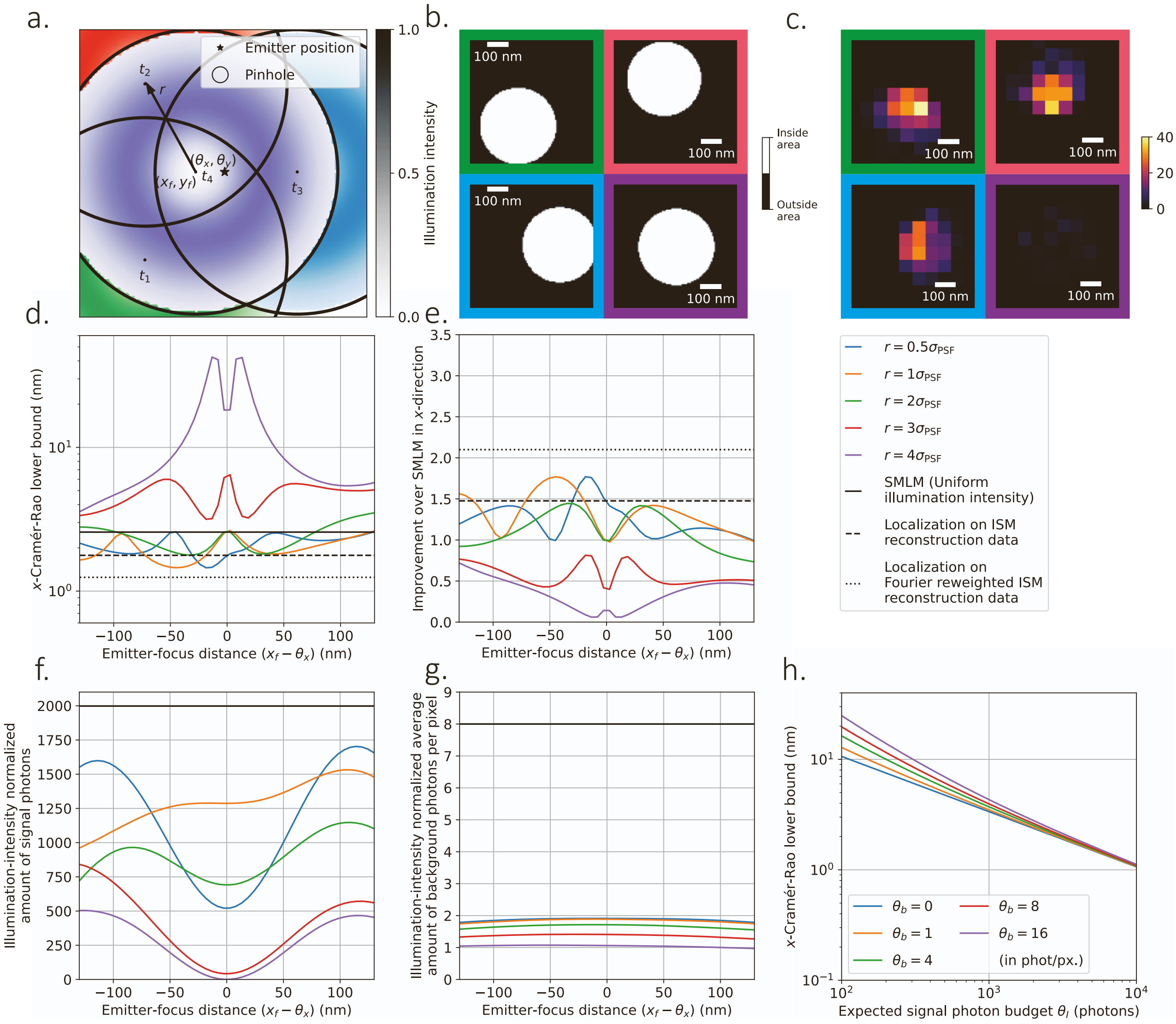

Figure S31: Theoretical minimum localization uncertainty of SpinFlux localization with four pinholes and donut-shaped patterns in a 90° rotated equilateral triangle configuration with a center pinhole. The pattern is rotated clockwise by 90 degrees with respect to Figure S26. In (c-g), we used 2000 expected signal photons and 8 expected background photons per pixel, with pinhole radius $r_p = 3\sigma_{PSF}$. Results are evaluated for the scenario where the illumination power and time are constant during illumination with all patterns. **(a)** Schematic overview of SpinFlux localization with a triangle of three pinholes with an additional center pinhole, centered at focus coordinates (x_f, y_f). In (d-g), the x-distance $(x_f - \theta_x)$ between the pattern focus and the emitter is varied, where $y_f = \theta_y$. **(b)** Example of pinholes in the region of interest (650 × 650 nm). The pinhole radius $r_p = 2\sigma_{PSF}$ and pinhole spacing $r = 1.5\sigma_{PSF}$ were used. The pinhole masks were discretized with $N_{M,x}$, $N_{M,y} = 100$ mesh pixels in each direction. **(c)** Example of fluorescent response in the region of interest, resulting from illumination and emission through each pinhole in (b). **(d)** Cramér-Rao lower bound (CRLB) in x-direction as a function of the emitter-focus x-distance. Simulations show SpinFlux with varying pinhole spacing and widefield single molecule localization microscopy (SMLM). **(e)** Improvement of the SpinFlux CRLB over SMLM as a function of the emitter-focus x-distance for varying pinhole spacing. **(f)** Average amount of signal photons after compensation for non-maximum illumination intensity as a function of the emitter-focus x-distance, for SpinFlux with varying pinhole spacing and widefield single molecule localization microscopy (SMLM). **(g)** Average amount of background photons per pixel after compensation for non-maximum illumination intensity as a function of the emitter-focus x-distance, for SpinFlux with varying pinhole spacing and widefield single molecule localization microscopy (SMLM). **(h)** CRLB in x-direction as a function of expected signal photon count for varying values of the expected background photon count. The pinhole radius $r_p = 3\sigma_{PSF}$ and pinhole spacing $r = 2\sigma_{PSF}$ were used and $(x_f, y_f) = (\theta_x, \theta_y)$.

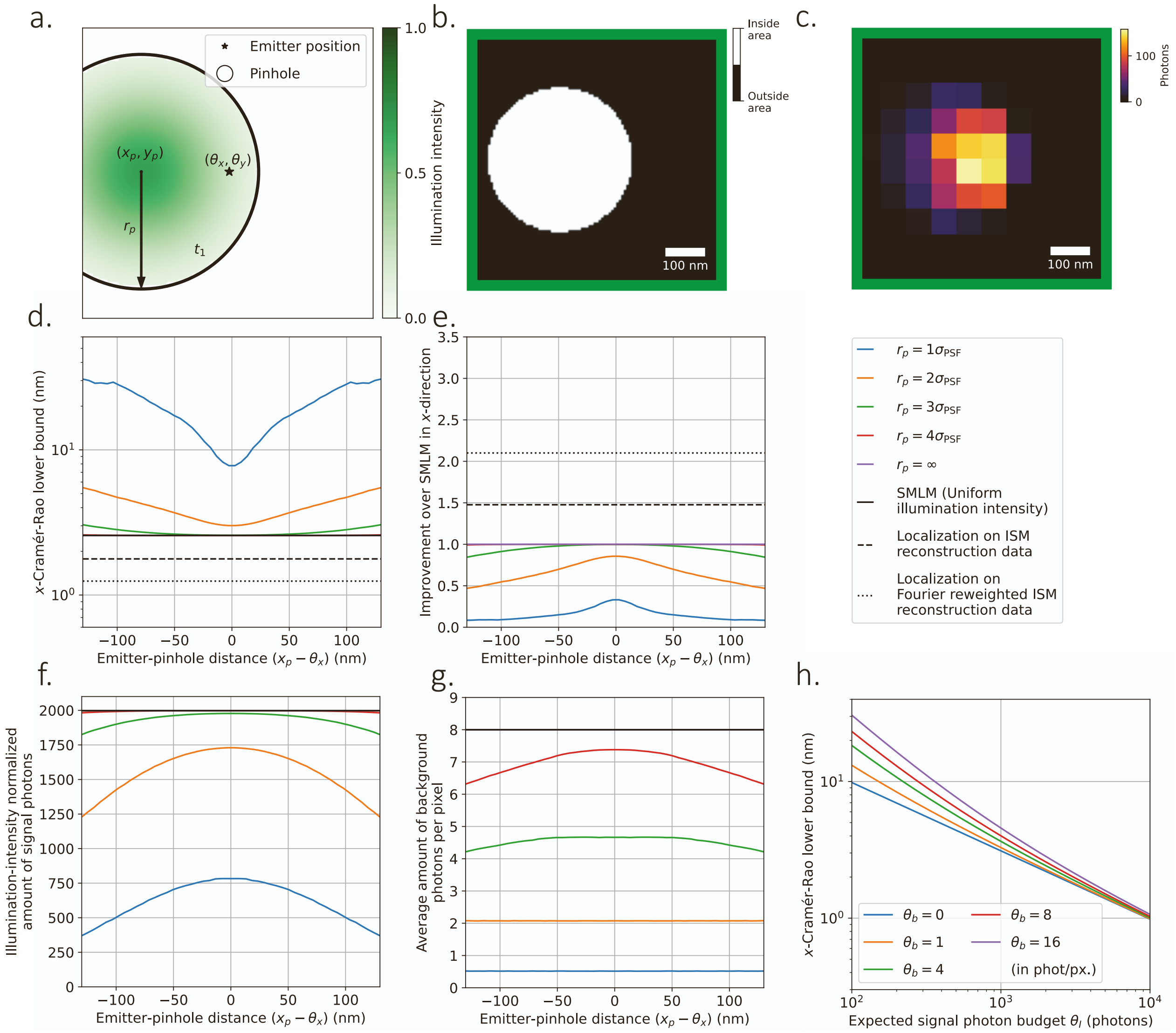

Figure S32: Theoretical minimum localization uncertainty of SpinFlux localization with one x-offset pinhole and pattern. In (c-g), 2000 expected signal photons and 8 expected background photons per pixel were used. Results are evaluated for the scenario where the entire signal photon budget is exhausted after illumination with the pattern (disregarding signal photons blocked by the spinning disk), neglecting the effects of pattern-dependent background. **(a)** Schematic overview of SpinFlux localization with one pinhole with radius r_p, centered at coordinates (x_p, y_p). In (d-g), the x-distance $(x_\text{p} - \theta_x)$ between the pinhole and the emitter is varied, where $y_\text{p} = \theta_y$. **(b)** Example of pinhole in the region of interest (650 × 650 nm). The pinhole radius $r_\text{p} = 2\sigma_\text{PSF}$ was used. The pinhole mask was discretized with $N_{\text{M},x}$, $N_{\text{M},y}$ = 100 mesh pixels in each direction. **(c)** Example of fluorescent response in the region of interest, resulting from illumination and emission through the pinhole in (b). **(d)** Cramér-Rao lower bound (CRLB) in x-direction as a function of the emitter-pinhole x-distance. Simulations show SpinFlux with varying pinhole sizes and widefield single-molecule localization microscopy (SMLM). **(e)** Improvement of the SpinFlux CRLB over SMLM as a function of the emitter-pinhole x-distance for varying pinhole sizes. **(f)** Average amount of signal photons after compensation for non-maximum illumination intensity as a function of the emitter-pinhole x-distance, for SpinFlux with varying pinhole sizes and widefield single molecule localization microscopy (SMLM). **(g)** Average amount of background photons per pixel as a function of the emitter-pinhole x-distance, for SpinFlux with varying pinhole sizes and widefield single molecule localization microscopy (SMLM). **(h)** CRLB in x-direction as a function of the expected signal photon count for varying values of the expected background photon count. The pinhole radius $r_\text{p} = 3\sigma_\text{PSF}$ was used and $(x_\text{p}, y_\text{p}) = (\theta_x, \theta_y)$.

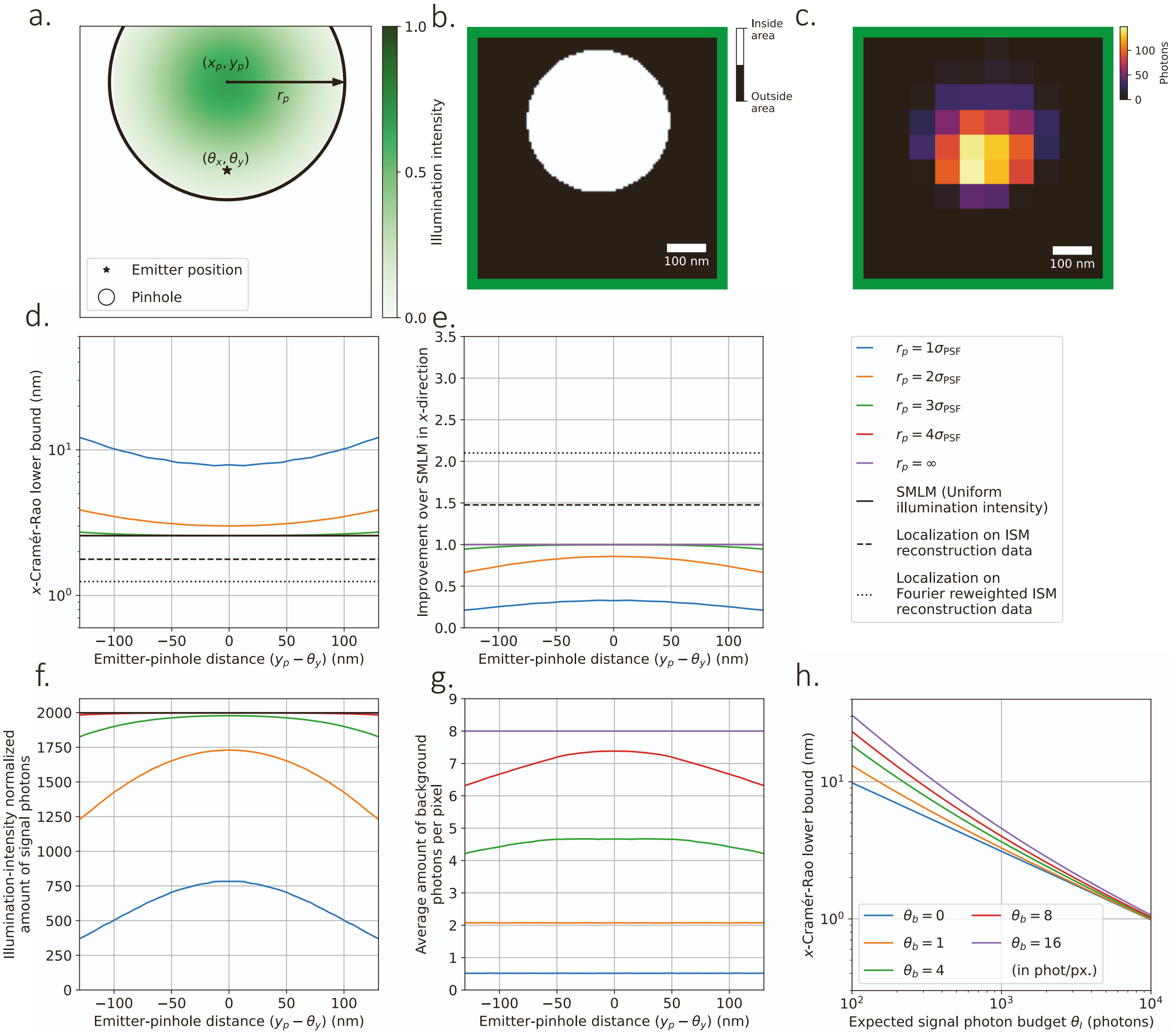

Figure S33: Theoretical minimum localization uncertainty of SpinFlux localization with one y-offset pinhole and pattern. In (c-g), 2000 expected signal photons and 8 expected background photons per pixel were used. Results are evaluated for the scenario where the entire signal photon budget is exhausted after illumination with the pattern (disregarding signal photons blocked by the spinning disk), neglecting the effects of pattern-dependent background. **(a)** Schematic overview of SpinFlux localization with one pinhole with radius r_p, centered at coordinates (x_p, y_p). In (d-g), the y-distance $(y_\text{p} - \theta_y)$ between the pinhole and the emitter is varied, where $x_\text{p} = \theta_x$. **(b)** Example of pinhole in the region of interest (650 × 650 nm). The pinhole radius $r_\text{p} = 2\sigma_\text{PSF}$ was used. The pinhole mask was discretized with $N_{\text{M},x}$, $N_{\text{M},y}$ = 100 mesh pixels in each direction. **(c)** Example of fluorescent response in the region of interest, resulting from illumination and emission through the pinhole in (b). **(d)** Cramér-Rao lower bound (CRLB) in x-direction as a function of the emitter-pinhole y-distance. Simulations show SpinFlux with varying pinhole sizes and widefield single-molecule localization microscopy (SMLM). **(e)** Improvement of the SpinFlux CRLB over SMLM as a function of the emitter-pinhole y-distance for varying pinhole sizes. **(f)** Average amount of signal photons after compensation for non-maximum illumination intensity as a function of the emitter-pinhole y-distance, for SpinFlux with varying pinhole sizes and widefield single molecule localization microscopy (SMLM). **(g)** Average amount of background photons per pixel as a function of the emitter-pinhole y-distance, for SpinFlux with varying pinhole sizes and widefield single molecule localization microscopy (SMLM). **(h)** CRLB in x-direction as a function of the expected signal photon count for varying values of the expected background photon count. The pinhole radius $r_\text{p} = 3\sigma_\text{PSF}$ was used and $(x_\text{p}, y_\text{p}) = (\theta_x, \theta_y)$.

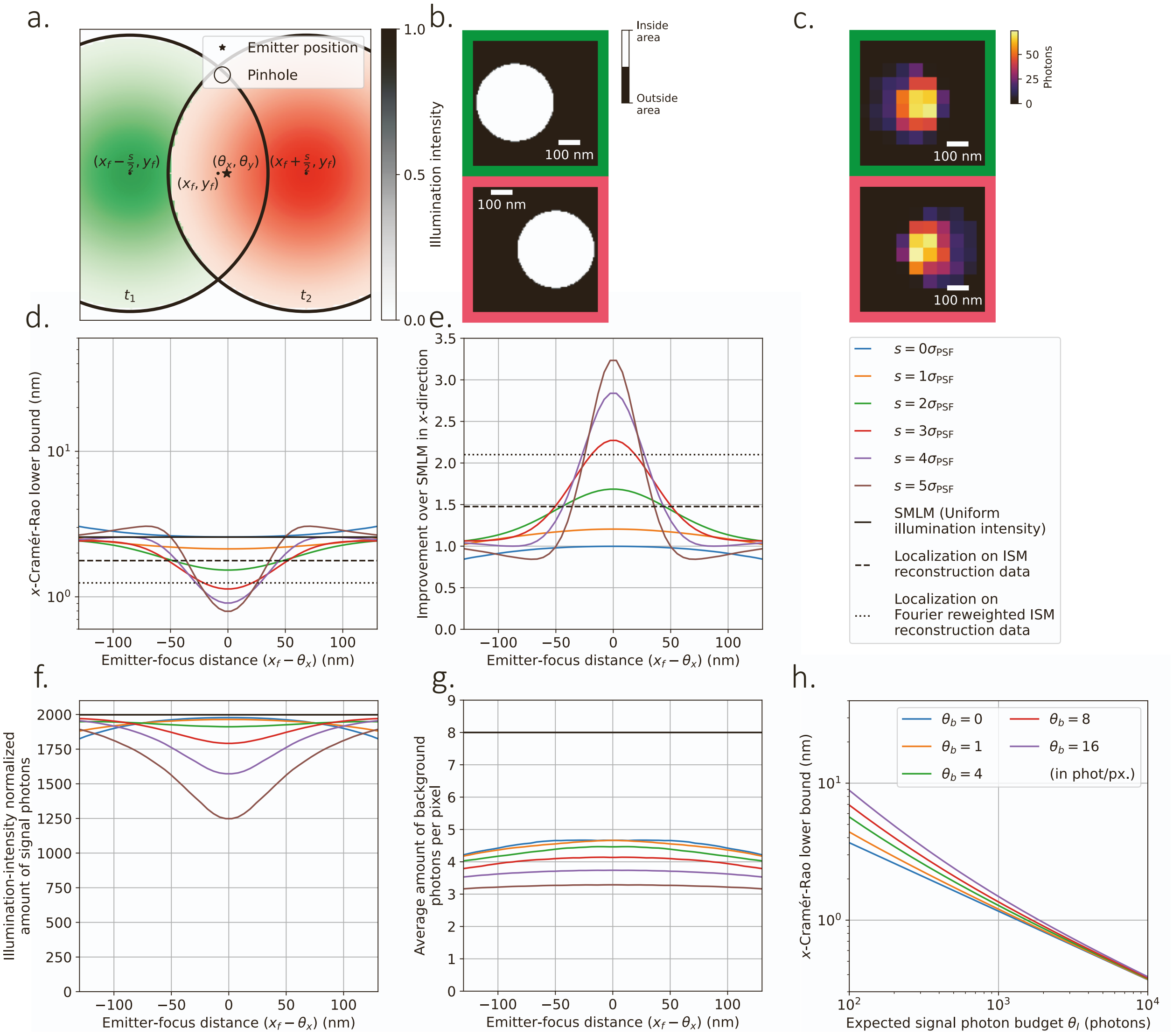

Figure S34: Theoretical minimum localization uncertainty of SpinFlux localization with two pinholes and patterns separated in the x-direction. In (c-g), 2000 expected signal photons and 8 expected background photons per pixel were used, with pinhole radius $r_p = 3\sigma_{PSF}$. Results are evaluated for the scenario where the entire signal photon budget is exhausted after illumination with all patterns (disregarding signal photons blocked by the spinning disk), neglecting the effects of pattern-dependent background. **(a)** Schematic overview of SpinFlux localization with two pinholes, separated in x and centered around the focus coordinates (x_f, y_f). In (d-g), the x-distance $(x_f - \theta_x)$ between the pattern focus and the emitter is varied, where $y_f = \theta_y$. **(b)** Example of pinholes in the region of interest (650 × 650 nm). The pinhole radius $r_p = 2\sigma_{PSF}$ and pinhole separation $s = 2\sigma_{PSF}$ were used. The pinhole masks were discretized with $N_{M,x}, N_{M,y} = 100$ mesh pixels in each direction. **(c)** Example of fluorescent response in the region of interest, resulting from illumination and emission through each pinhole in (b). **(d)** Cramér-Rao lower bound (CRLB) in x-direction as a function of the emitter-focus x-distance. Simulations show SpinFlux with varying pinhole separations and widefield single molecule localization microscopy (SMLM). **(e)** Improvement of the SpinFlux CRLB over SMLM as a function of the emitter-focus x-distance for varying pinhole separations. **(f)** Average amount of signal photons after compensation for non-maximum illumination intensity as a function of the emitter-focus x-distance, for SpinFlux with varying pinhole separations and widefield single molecule localization microscopy (SMLM). **(g)** Average amount of background photons per pixel as a function of the emitter-focus x-distance, for SpinFlux with varying pinhole separations and widefield single molecule localization microscopy (SMLM). **(h)** CRLB in x-direction as a function of expected signal photon count for varying values of the expected background photon count. The pinhole radius $r_p = 3\sigma_{PSF}$ and pinhole separation $s = 4\sigma_{PSF}$ were used and $(x_f, y_f) = (\theta_x, \theta_y)$.

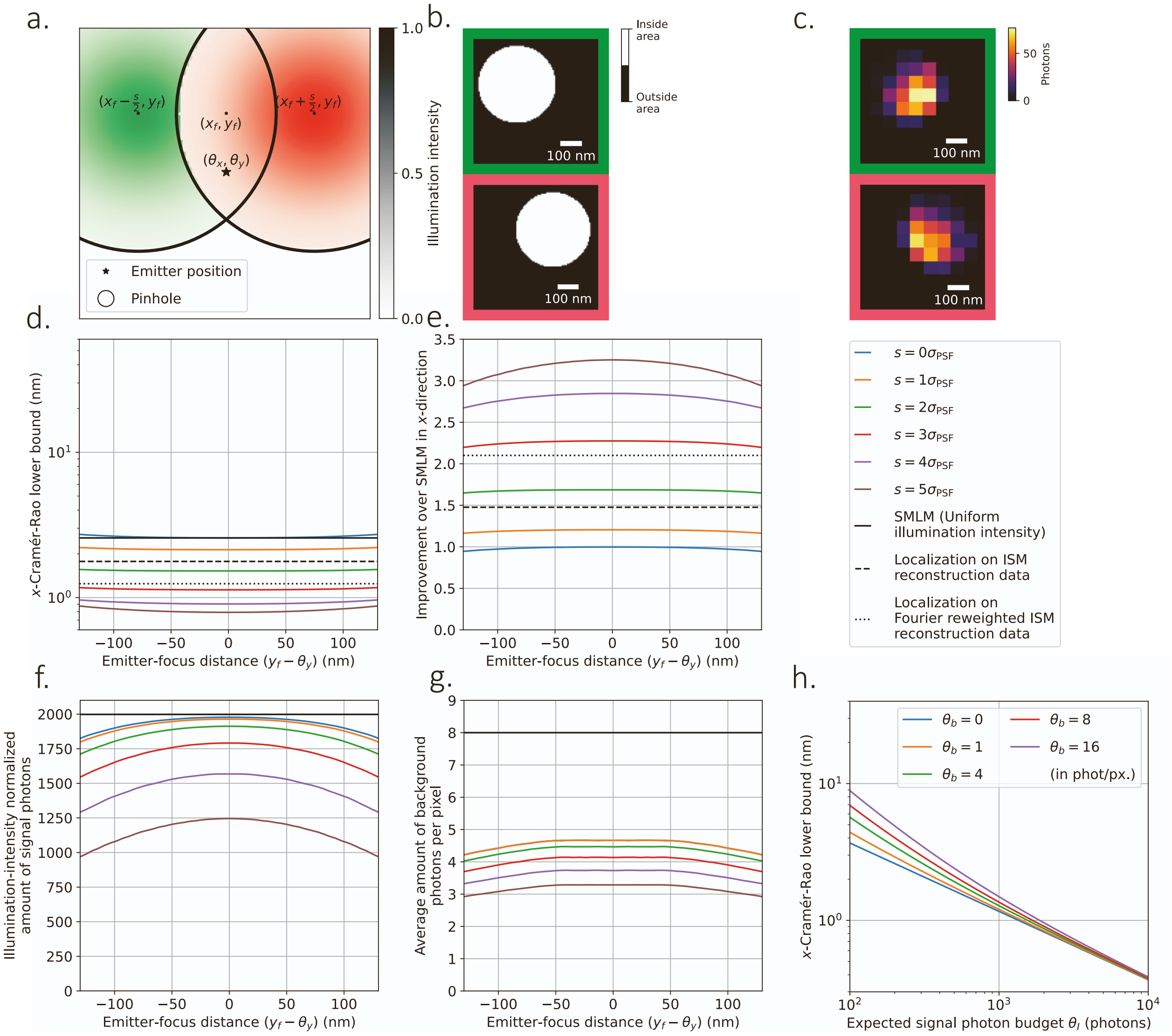

Figure S35: Theoretical minimum localization uncertainty of SpinFlux localization with two y-offset pinholes and patterns separated in the x-direction. In (c-g), 2000 expected signal photons and 8 expected background photons per pixel were used, with pinhole radius $r_p = 3\sigma_{PSF}$. Results are evaluated for the scenario where the entire signal photon budget is exhausted after illumination with all patterns (disregarding signal photons blocked by the spinning disk), neglecting the effects of pattern-dependent background. **(a)** Schematic overview of SpinFlux localization with two pinholes, separated in x and centered around the focus coordinates (x_f, y_f). In (d-g), the y-distance $(y_f - \theta_y)$ between the pattern focus and the emitter is varied, where $x_f = \theta_x$. **(b)** Example of pinholes in the region of interest (650 × 650 nm). The pinhole radius $r_p = 2\sigma_{PSF}$ and pinhole separation $s = 2\sigma_{PSF}$ were used. The pinhole masks were discretized with $N_{M,x}$, $N_{M,y} = 100$ mesh pixels in each direction. **(c)** Example of fluorescent response in the region of interest, resulting from illumination and emission through each pinhole in (b). **(d)** Cramér-Rao lower bound (CRLB) in x-direction as a function of the emitter-focus y-distance. Simulations show SpinFlux with varying pinhole separations and widefield single molecule localization microscopy (SMLM). **(e)** Improvement of the SpinFlux CRLB over SMLM as a function of the emitter-focus y-distance for varying pinhole separations. **(f)** Average amount of signal photons after compensation for non-maximum illumination intensity as a function of the emitter-focus y-distance, for SpinFlux with varying pinhole separations and widefield single molecule localization microscopy (SMLM). **(g)** Average amount of background photons per pixel as a function of the emitter-focus y-distance, for SpinFlux with varying pinhole separations and widefield single molecule localization microscopy (SMLM). **(h)** CRLB in x-direction as a function of expected signal photon count for varying values of the expected background photon count. The pinhole radius $r_p = 3\sigma_{PSF}$ and pinhole separation $s = 4\sigma_{PSF}$ were used and $(x_f, y_f) = (\theta_x, \theta_y)$.

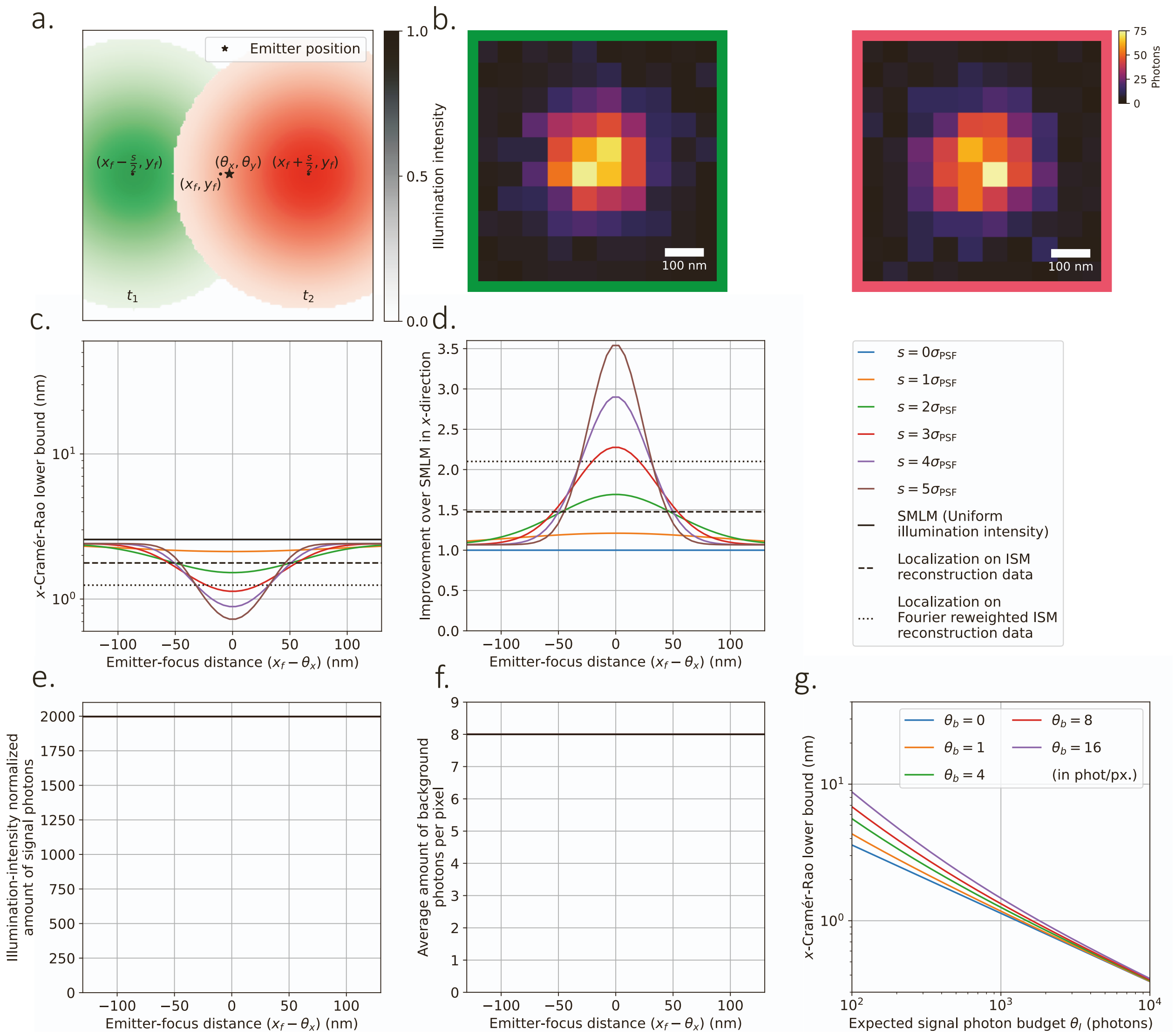

Figure S36: Theoretical minimum localization uncertainty of SpinFlux localization with two patterns without pinholes separated in the x-direction. In (b-f), 2000 expected signal photons and 8 expected background photons per pixel were used. Results are evaluated for the scenario where the entire signal photon budget is exhausted after illumination with all patterns, neglecting the effects of pattern-dependent background. **(a)** Schematic overview of SpinFlux localization with two pinholes, separated in x and centered around the focus coordinates (x_f, y_f). In (c-f), the x-distance $(x_f - \theta_x)$ between the pattern focus and the emitter is varied, where $y_f = \theta_y$. **(b)** Example of fluorescent response in the region of interest, resulting from illumination and emission by each pattern in (a). **(c)** Cramér-Rao lower bound (CRLB) in x-direction as a function of the emitter-focus x-distance. Simulations show SpinFlux with varying pinhole separations and widefield single molecule localization microscopy (SMLM). **(d)** Improvement of the SpinFlux CRLB over SMLM as a function of the emitter-focus x-distance for varying pinhole separations. **(e)** Average amount of signal photons after compensation for non-maximum illumination intensity as a function of the emitter-focus x-distance, for SpinFlux with varying pinhole separations and widefield single molecule localization microscopy (SMLM). **(f)** Average amount of background photons per pixel as a function of the emitter-focus x-distance, for SpinFlux with varying pinhole separations and widefield single molecule localization microscopy (SMLM). **(g)** CRLB in x-direction as a function of expected signal photon count for varying values of the expected background photon count. The pattern separation $s = 4\sigma_{PSF}$ was used and $(x_f, y_f) = (\theta_x, \theta_y)$.

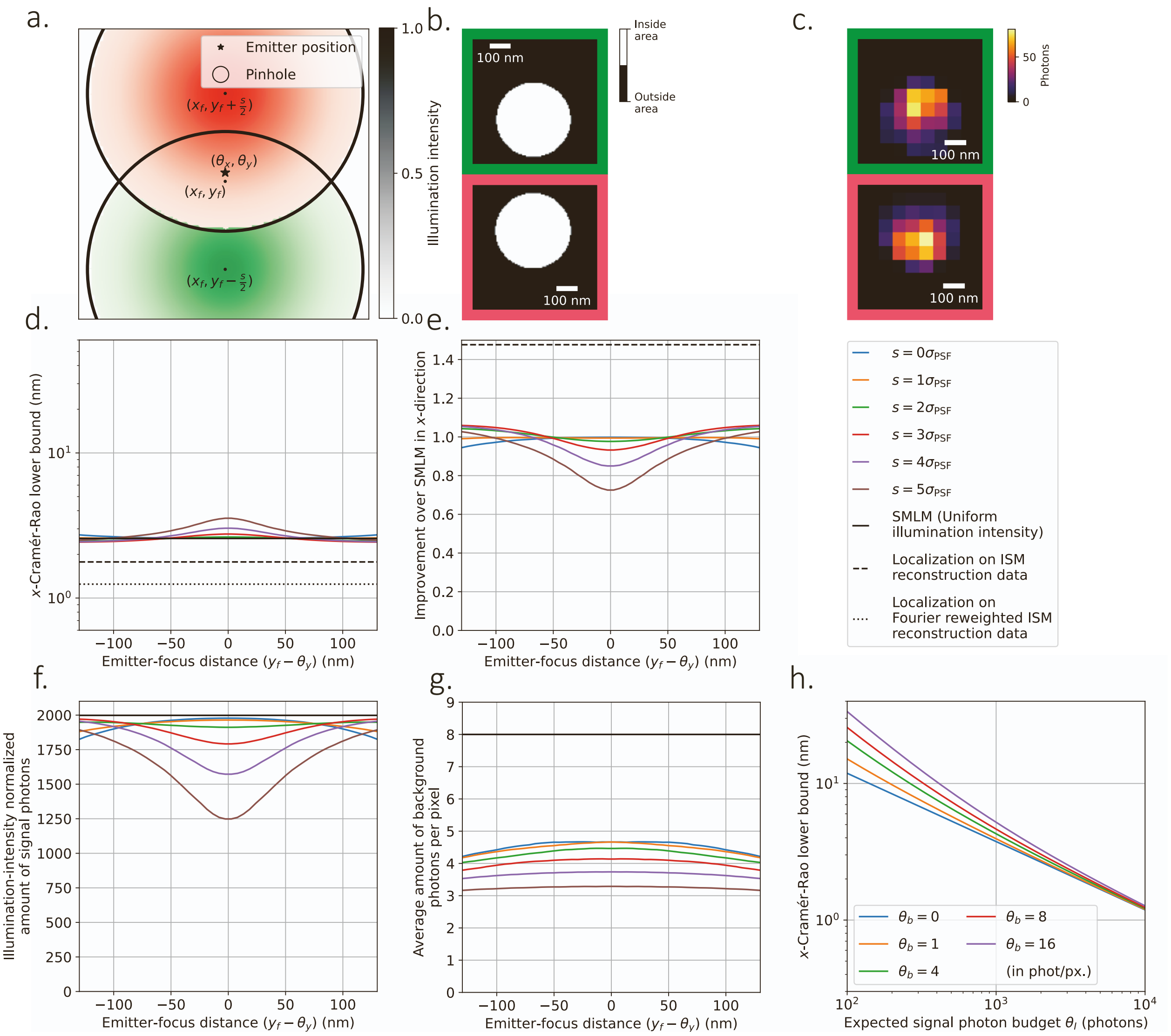

Figure S37: Theoretical minimum localization uncertainty of SpinFlux localization with two pinholes and patterns separated in the y-direction. In (c-g), 2000 expected signal photons and 8 expected background photons per pixel were used, with pinhole radius $r_p = 3\sigma_{PSF}$. Results are evaluated for the scenario where the entire signal photon budget is exhausted after illumination with all patterns (disregarding signal photons blocked by the spinning disk), neglecting the effects of pattern-dependent background. **(a)** Schematic overview of SpinFlux localization with two pinholes, separated in y and centered around the focus coordinates (x_f, y_f). In (d-g), the y-distance $(y_f - \theta_y)$ between the pattern focus and the emitter is varied, where $x_f = \theta_x$. **(b)** Example of pinholes in the region of interest (650 × 650 nm). The pinhole radius $r_p = 2\sigma_{PSF}$ and pinhole separation $s = 2\sigma_{PSF}$ were used. The pinhole masks were discretized with $N_{M,x}, N_{M,y} = 100$ mesh pixels in each direction. **(c)** Example of fluorescent response in the region of interest, resulting from illumination and emission through each pinhole in (b). **(d)** Cramér-Rao lower bound (CRLB) in x-direction as a function of the emitter-focus y-distance. Simulations show SpinFlux with varying pinhole separations and widefield single molecule localization microscopy (SMLM). **(e)** Improvement of the SpinFlux CRLB over SMLM as a function of the emitter-focus y-distance for varying pinhole separations. **(f)** Average amount of signal photons after compensation for non-maximum illumination intensity as a function of the emitter-focus y-distance, for SpinFlux with varying pinhole separations and widefield single molecule localization microscopy (SMLM). **(g)** Average amount of background photons per pixel as a function of the emitter-focus y-distance, for SpinFlux with varying pinhole separations and widefield single molecule localization microscopy (SMLM). **(h)** CRLB in x-direction as a function of expected signal photon count for varying values of the expected background photon count. The pinhole radius $r_p = 3\sigma_{PSF}$ and pinhole separation $s = 4\sigma_{PSF}$ were used and $(x_f, y_f) = (\theta_x, \theta_y)$.

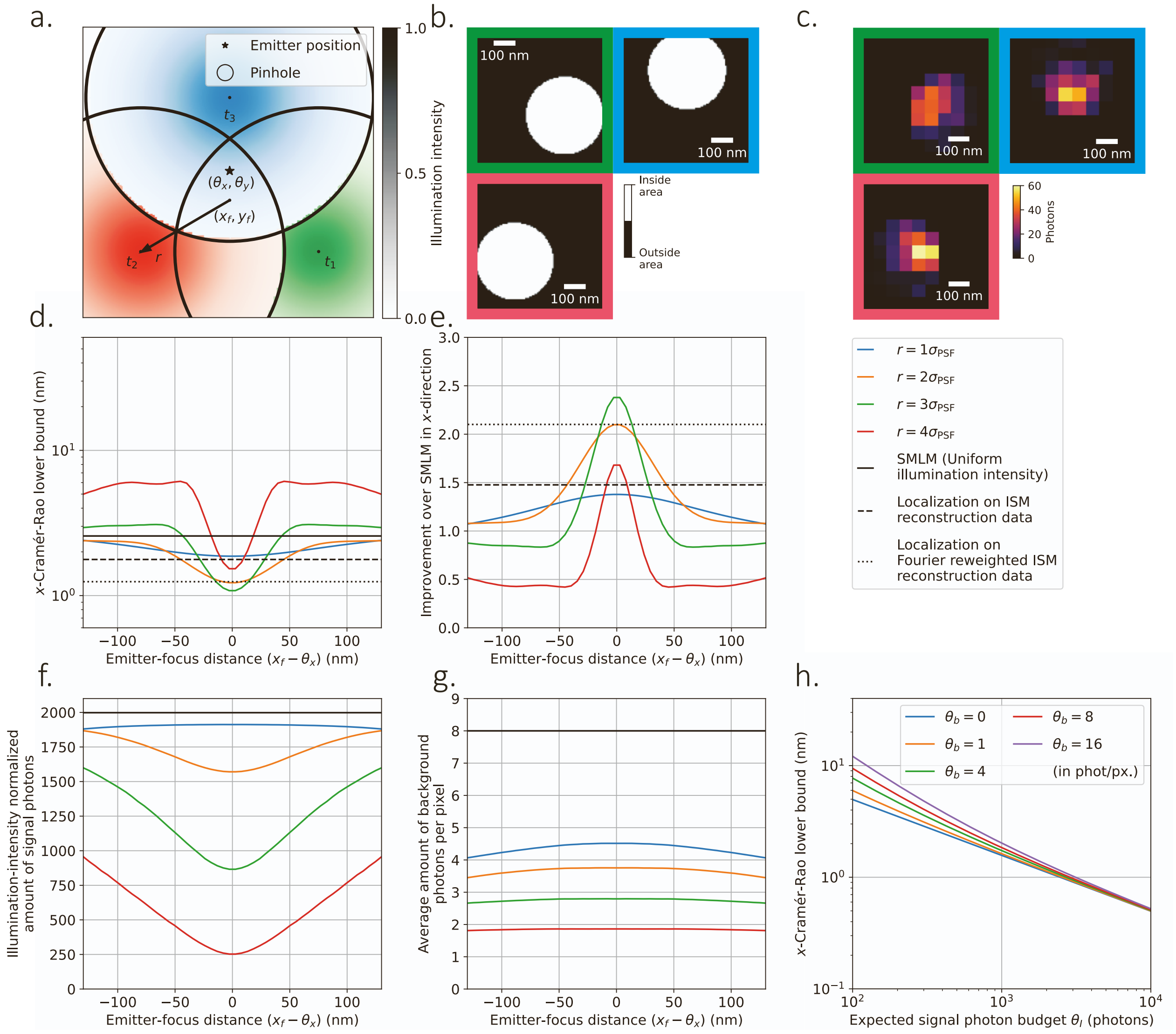

Figure S38: Theoretical minimum localization uncertainty of SpinFlux localization with three pinholes and patterns in an equilateral triangle configuration. In (c-g), we used 2000 expected signal photons and 8 expected background photons per pixel, with pinhole radius $r_p = 3\sigma_{PSF}$. Results are evaluated for the scenario where the entire signal photon budget is exhausted after illumination with all patterns (disregarding signal photons blocked by the spinning disk), neglecting the effects of pattern-dependent background. **(a)** Schematic overview of SpinFlux localization with a triangle of three pinholes, centered at focus coordinates (x_f, y_f). In (d-g), the x-distance $(x_f - \theta_x)$ between the pattern focus and the emitter is varied, where $y_f = \theta_y$. **(b)** Example of pinholes in the region of interest (650 × 650 nm). The pinhole radius $r_p = 2\sigma_{PSF}$ and pinhole spacing $r = 1.5\sigma_{PSF}$ were used. The pinhole masks were discretized with $N_{M,x}$, $N_{M,y} = 100$ mesh pixels in each direction. **(c)** Example of fluorescent response in the region of interest, resulting from illumination and emission through each pinhole in (b). **(d)** Cramér-Rao lower bound (CRLB) in x-direction as a function of the emitter-focus x-distance. Simulations show SpinFlux with varying pinhole spacing and widefield single molecule localization microscopy (SMLM). **(e)** Improvement of the SpinFlux CRLB over SMLM as a function of the emitter-focus x-distance for varying pinhole spacing. **(f)** Average amount of signal photons after compensation for non-maximum illumination intensity as a function of the emitter-focus x-distance, for SpinFlux with varying pinhole spacing and widefield single molecule localization microscopy (SMLM). **(g)** Average amount of background photons per pixel as a function of the emitter-focus x-distance, for SpinFlux with varying pinhole spacing and widefield single molecule localization microscopy (SMLM). **(h)** CRLB in x-direction as a function of expected signal photon count for varying values of the expected background photon count. The pinhole radius $r_p = 3\sigma_{PSF}$ and pinhole spacing $r = 2\sigma_{PSF}$ were used and $(x_f, y_f) = (\theta_x, \theta_y)$.

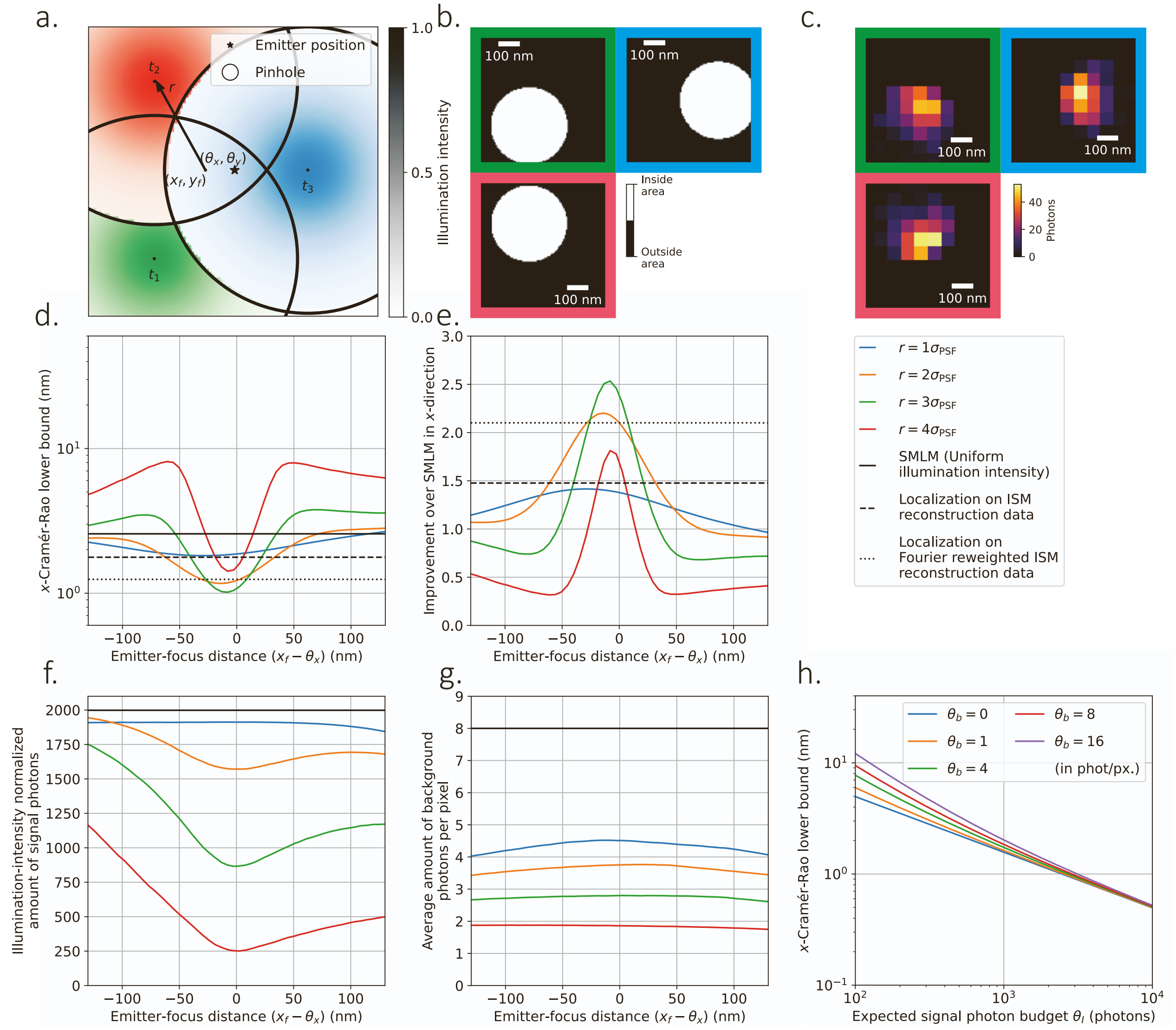

Figure S39: Theoretical minimum localization uncertainty of SpinFlux localization with three pinholes and patterns in a 90° rotated equilateral triangle configuration. The pattern is rotated clockwise by 90 degrees with respect to Figure S38. In (c-g), we used 2000 expected signal photons and 8 expected background photons per pixel, with pinhole radius $r_p = 3\sigma_{PSF}$. Results are evaluated for the scenario where the entire signal photon budget is exhausted after illumination with all patterns (disregarding signal photons blocked by the spinning disk), neglecting the effects of pattern-dependent background. **(a)** Schematic overview of SpinFlux localization with a triangle of three pinholes, centered at focus coordinates (x_f, y_f). In (d-g), the x-distance $(x_f - \theta_x)$ between the pattern focus and the emitter is varied, where $y_f = \theta_y$. **(b)** Example of pinholes in the region of interest (650 × 650 nm). The pinhole radius $r_p = 2\sigma_{PSF}$ and pinhole spacing $r = 1.5\sigma_{PSF}$ were used. The pinhole masks were discretized with $N_{M,x}, N_{M,y} = 100$ mesh pixels in each direction. **(c)** Example of fluorescent response in the region of interest, resulting from illumination and emission through each pinhole in (b). **(d)** Cramér-Rao lower bound (CRLB) in x-direction as a function of the emitter-focus x-distance. Simulations show SpinFlux with varying pinhole spacing and widefield single molecule localization microscopy (SMLM). **(e)** Improvement of the SpinFlux CRLB over SMLM as a function of the emitter-focus x-distance for varying pinhole spacing. **(f)** Average amount of signal photons after compensation for non-maximum illumination intensity as a function of the emitter-focus x-distance, for SpinFlux with varying pinhole spacing and widefield single molecule localization microscopy (SMLM). **(g)** Average amount of background photons per pixel as a function of the emitter-focus x-distance, for SpinFlux with varying pinhole spacing and widefield single molecule localization microscopy (SMLM). **(h)** CRLB in x-direction as a function of expected signal photon count for varying values of the expected background photon count. The pinhole radius $r_p = 3\sigma_{PSF}$ and pinhole spacing $r = 2\sigma_{PSF}$ were used and $(x_f, y_f) = (\theta_x, \theta_y)$.

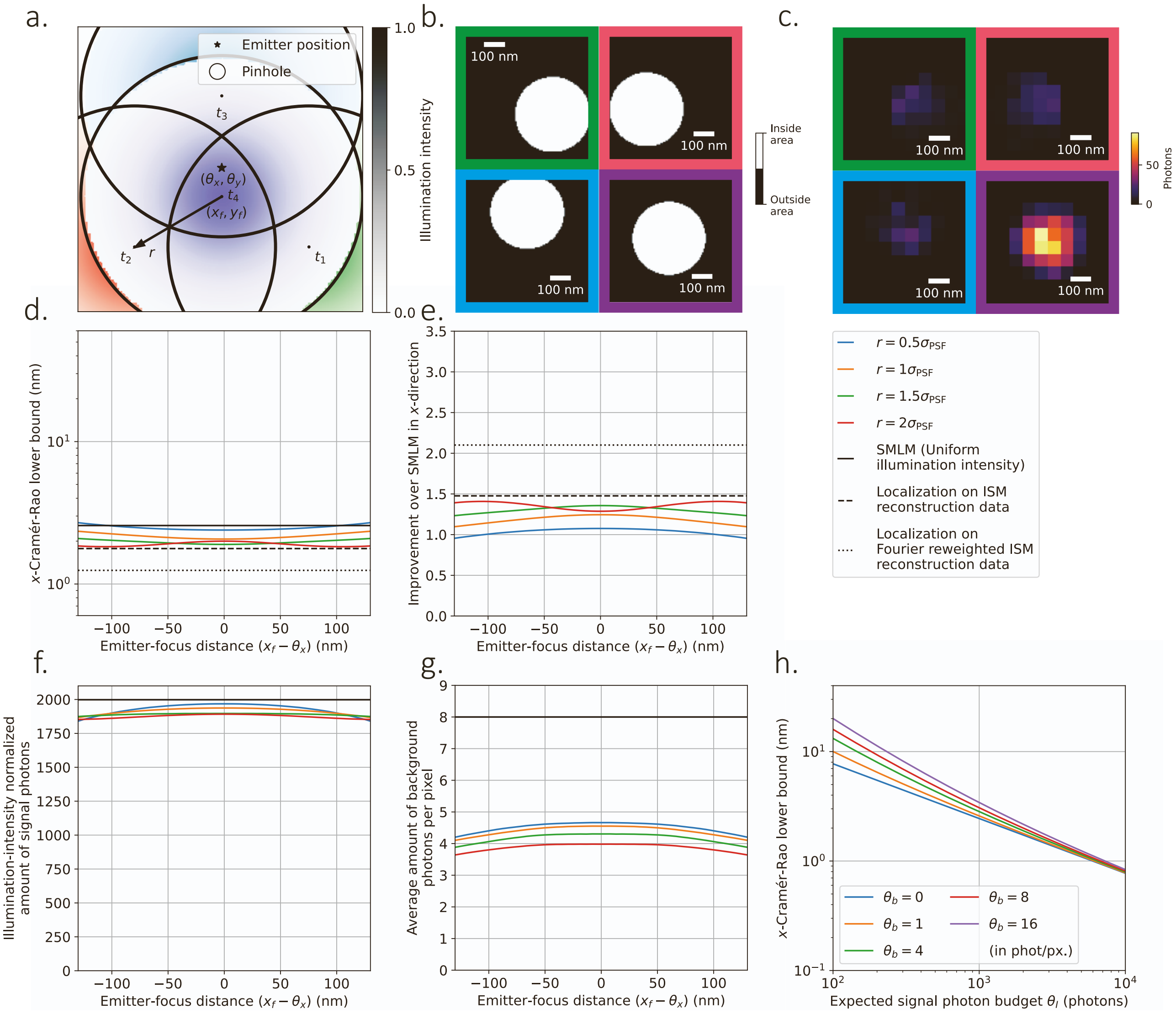

Figure S40: Theoretical minimum localization uncertainty of SpinFlux localization with four pinholes and patterns in an equilateral triangle configuration with a center pinhole. In (c-g), we used 2000 expected signal photons and 8 expected background photons per pixel, with pinhole radius $r_p = 3\sigma_{PSF}$. Results are evaluated for the scenario where the entire signal photon budget is exhausted after illumination with all patterns (disregarding signal photons blocked by the spinning disk), neglecting the effects of pattern-dependent background. **(a)** Schematic overview of SpinFlux localization with a triangle of three pinholes with an additional center pinhole, centered at focus coordinates (x_f, y_f). In (d-g), the x-distance $(x_f - \theta_x)$ between the pattern focus and the emitter is varied, where $y_f = \theta_y$. **(b)** Example of pinholes in the region of interest (650 × 650 nm). The pinhole radius $r_p = 2\sigma_{PSF}$ and pinhole spacing $r = 1.5\sigma_{PSF}$ were used. The pinhole masks were discretized with $N_{M,x}, N_{M,y} = 100$ mesh pixels in each direction. **(c)** Example of fluorescent response in the region of interest, resulting from illumination and emission through each pinhole in (b). **(d)** Cramér-Rao lower bound (CRLB) in x-direction as a function of the emitter-focus x-distance. Simulations show SpinFlux with varying pinhole spacing and widefield single molecule localization microscopy (SMLM). **(e)** Improvement of the SpinFlux CRLB over SMLM as a function of the emitter-focus x-distance for varying pinhole spacing. **(f)** Average amount of signal photons after compensation for non-maximum illumination intensity as a function of the emitter-focus x-distance, for SpinFlux with varying pinhole spacing and widefield single molecule localization microscopy (SMLM). **(g)** Average amount of background photons per pixel as a function of the emitter-focus x-distance, for SpinFlux with varying pinhole spacing and widefield single molecule localization microscopy (SMLM). **(h)** CRLB in x-direction as a function of expected signal photon count for varying values of the expected background photon count. The pinhole radius $r_p = 3\sigma_{PSF}$ and pinhole spacing $r = 2\sigma_{PSF}$ were used and $(x_f, y_f) = (\theta_x, \theta_y)$.

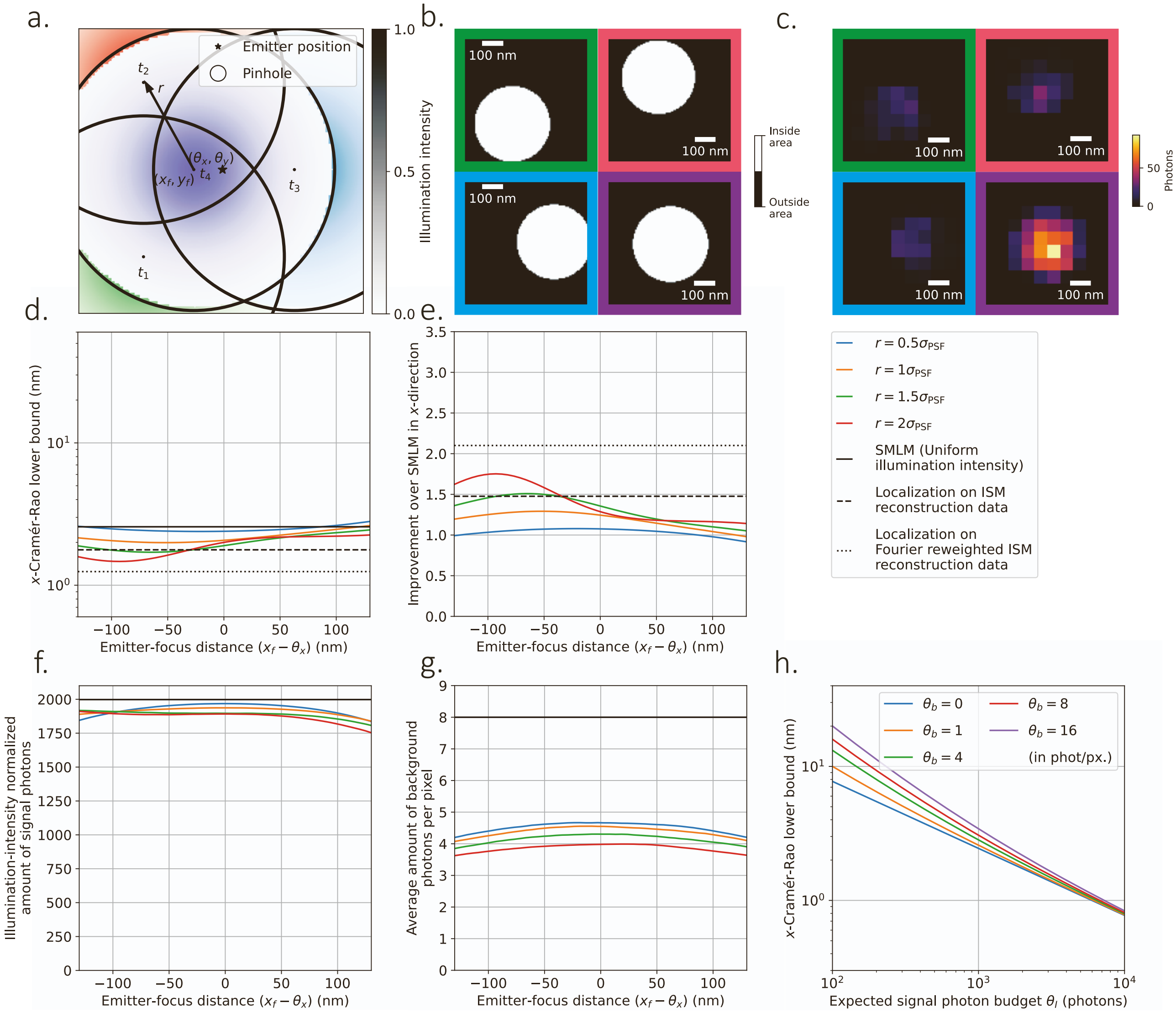

Figure S41: Theoretical minimum localization uncertainty of SpinFlux localization with four pinholes and patterns in a 90° rotated equilateral triangle configuration with a center pinhole. The pattern is rotated clockwise by 90 degrees with respect to Figure S40. In (c-g), we used 2000 expected signal photons and 8 expected background photons per pixel, with pinhole radius $r_p = 3\sigma_{PSF}$. Results are evaluated for the scenario where the entire signal photon budget is exhausted after illumination with all patterns (disregarding signal photons blocked by the spinning disk), neglecting the effects of pattern-dependent background. **(a)** Schematic overview of SpinFlux localization with a triangle of three pinholes with an additional center pinhole, centered at focus coordinates (x_f, y_f). In (d-g), the x-distance $(x_f - \theta_x)$ between the pattern focus and the emitter is varied, where $y_f = \theta_y$. **(b)** Example of pinholes in the region of interest (650 × 650 nm). The pinhole radius $r_p = 2\sigma_{PSF}$ and pinhole spacing $r = 1.5\sigma_{PSF}$ were used. The pinhole masks were discretized with $N_{M,x}$, $N_{M,y} = 100$ mesh pixels in each direction. **(c)** Example of fluorescent response in the region of interest, resulting from illumination and emission through each pinhole in (b). **(d)** Cramér-Rao lower bound (CRLB) in x-direction as a function of the emitter-focus x-distance. Simulations show SpinFlux with varying pinhole spacing and widefield single molecule localization microscopy (SMLM). **(e)** Improvement of the SpinFlux CRLB over SMLM as a function of the emitter-focus x-distance for varying pinhole spacing. **(f)** Average amount of signal photons after compensation for non-maximum illumination intensity as a function of the emitter-focus x-distance, for SpinFlux with varying pinhole spacing and widefield single molecule localization microscopy (SMLM). **(g)** Average amount of background photons per pixel as a function of the emitter-focus x-distance, for SpinFlux with varying pinhole spacing and widefield single molecule localization microscopy (SMLM). **(h)** CRLB in x-direction as a function of expected signal photon count for varying values of the expected background photon count. The pinhole radius $r_p = 3\sigma_{PSF}$ and pinhole spacing $r = 2\sigma_{PSF}$ were used and $(x_f, y_f) = (\theta_x, \theta_y)$.

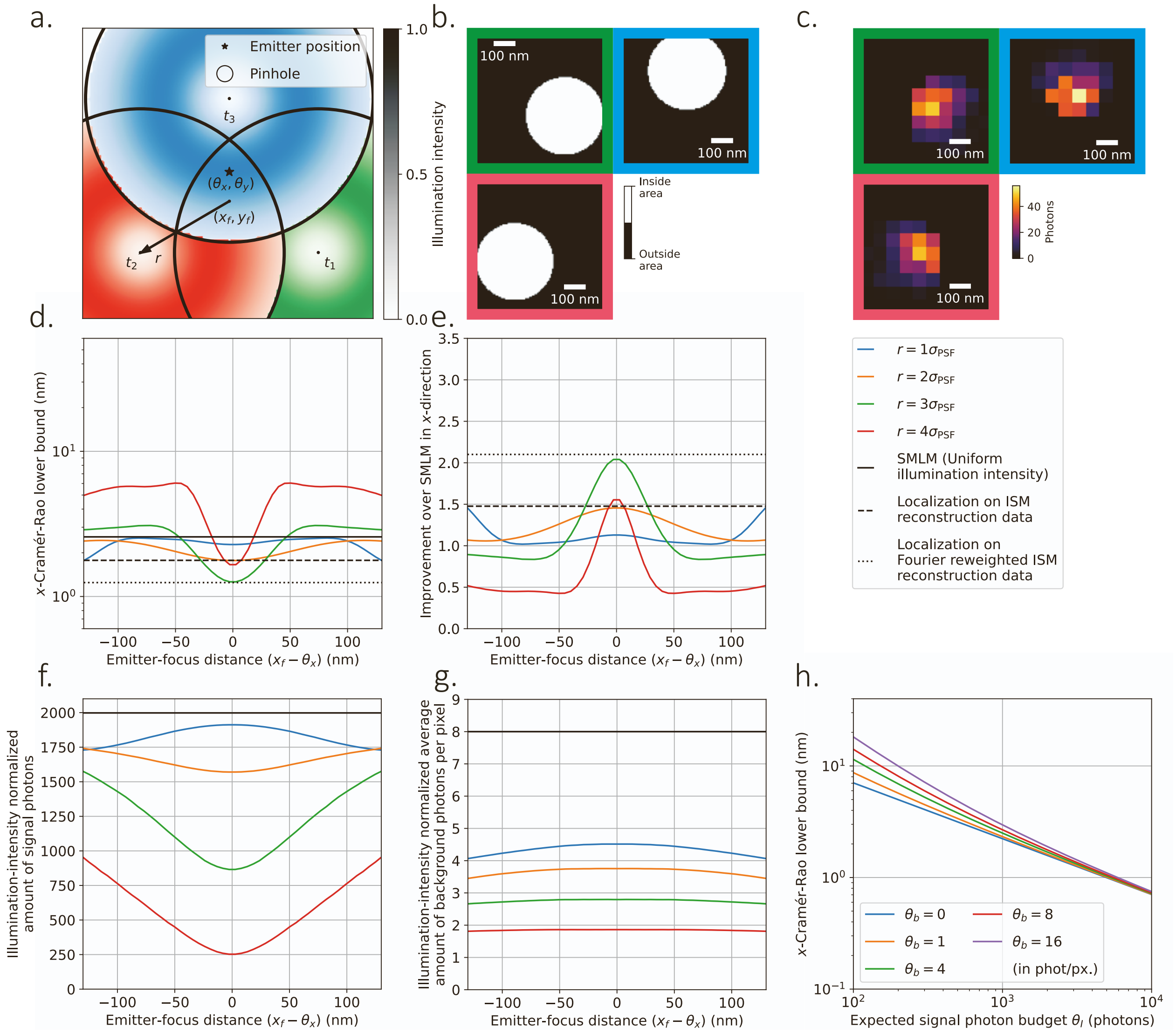

Figure S42: Theoretical minimum localization uncertainty of SpinFlux localization with three pinholes and donut-shaped patterns in an equilateral triangle configuration. In (c-g), we used 2000 expected signal photons and 8 expected background photons per pixel, with pinhole radius $r_p = 3\sigma_{PSF}$. Results are evaluated for the scenario where the entire signal photon budget is exhausted after illumination with all patterns (disregarding signal photons blocked by the spinning disk), neglecting the effects of pattern-dependent background. **(a)** Schematic overview of SpinFlux localization with a triangle of three pinholes, centered at focus coordinates (x_f, y_f). In (d-g), the x-distance $(x_f - \theta_x)$ between the pattern focus and the emitter is varied, where $y_f = \theta_y$. **(b)** Example of pinholes in the region of interest (650 × 650 nm). The pinhole radius $r_p = 2\sigma_{PSF}$ and pinhole spacing $r = 1.5\sigma_{PSF}$ were used. The pinhole masks were discretized with $N_{M,x}$, $N_{M,y} = 100$ mesh pixels in each direction. **(c)** Example of fluorescent response in the region of interest, resulting from illumination and emission through each pinhole in (b). **(d)** Cramér-Rao lower bound (CRLB) in x-direction as a function of the emitter-focus x-distance. Simulations show SpinFlux with varying pinhole spacing and widefield single molecule localization microscopy (SMLM). **(e)** Improvement of the SpinFlux CRLB over SMLM as a function of the emitter-focus x-distance for varying pinhole spacing. **(f)** Average amount of signal photons after compensation for non-maximum illumination intensity as a function of the emitter-focus x-distance, for SpinFlux with varying pinhole spacing and widefield single molecule localization microscopy (SMLM). **(g)** Average amount of background photons per pixel as a function of the emitter-focus x-distance, for SpinFlux with varying pinhole spacing and widefield single molecule localization microscopy (SMLM). **(h)** CRLB in x-direction as a function of expected signal photon count for varying values of the expected background photon count. The pinhole radius $r_p = 3\sigma_{PSF}$ and pinhole spacing $r = 2\sigma_{PSF}$ were used and $(x_f, y_f) = (\theta_x, \theta_y)$.

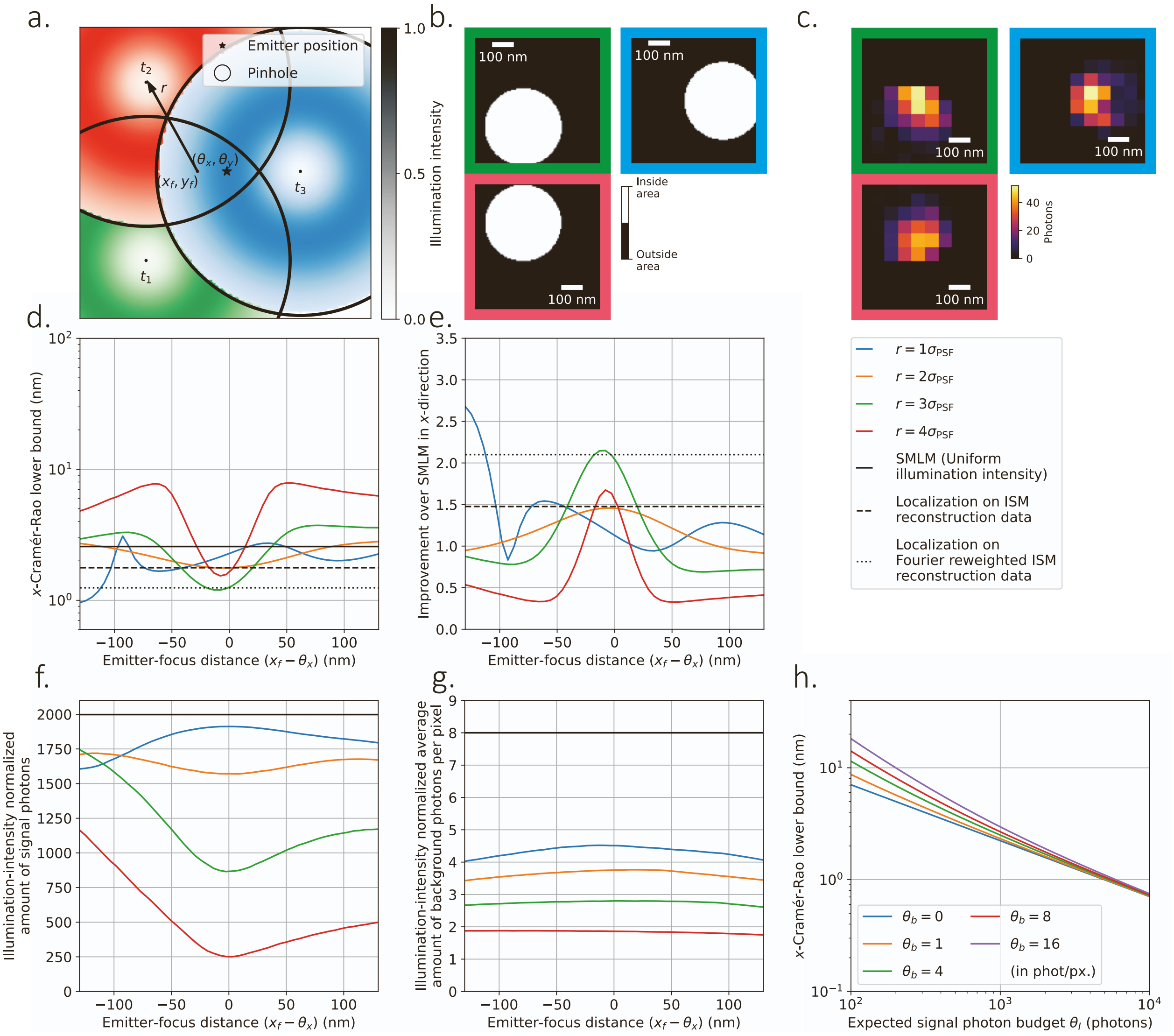

Figure S43: Theoretical minimum localization uncertainty of SpinFlux localization with three pinholes and soughnut-shaped patterns in a 90° rotated equilateral triangle configuration. The pattern is rotated clockwise by 90 degrees with respect to Figure S38. In (c-g), we used 2000 expected signal photons and 8 expected background photons per pixel, with pinhole radius $r_p = 3\sigma_{PSF}$. Results are evaluated for the scenario where the entire signal photon budget is exhausted after illumination with all patterns (disregarding signal photons blocked by the spinning disk), neglecting the effects of pattern-dependent background. **(a)** Schematic overview of SpinFlux localization with a triangle of three pinholes, centered at focus coordinates (x_f, y_f). In (d-g), the x-distance $(x_f - \theta_x)$ between the pattern focus and the emitter is varied, where $y_f = \theta_y$. **(b)** Example of pinholes in the region of interest (650 × 650 nm). The pinhole radius $r_p = 2\sigma_{PSF}$ and pinhole spacing $r = 1.5\sigma_{PSF}$ were used. The pinhole masks were discretized with $N_{M,x}$, $N_{M,y} = 100$ mesh pixels in each direction. **(c)** Example of fluorescent response in the region of interest, resulting from illumination and emission through each pinhole in (b). **(d)** Cramér-Rao lower bound (CRLB) in x-direction as a function of the emitter-focus x-distance. Simulations show SpinFlux with varying pinhole spacing and widefield single molecule localization microscopy (SMLM). **(e)** Improvement of the SpinFlux CRLB over SMLM as a function of the emitter-focus x-distance for varying pinhole spacing. **(f)** Average amount of signal photons after compensation for non-maximum illumination intensity as a function of the emitter-focus x-distance, for SpinFlux with varying pinhole spacing and widefield single molecule localization microscopy (SMLM). **(g)** Average amount of background photons per pixel as a function of the emitter-focus x-distance, for SpinFlux with varying pinhole spacing and widefield single molecule localization microscopy (SMLM). **(h)** CRLB in x-direction as a function of expected signal photon count for varying values of the expected background photon count. The pinhole radius $r_p = 3\sigma_{PSF}$ and pinhole spacing $r = 2\sigma_{PSF}$ were used and $(x_f, y_f) = (\theta_x, \theta_y)$.

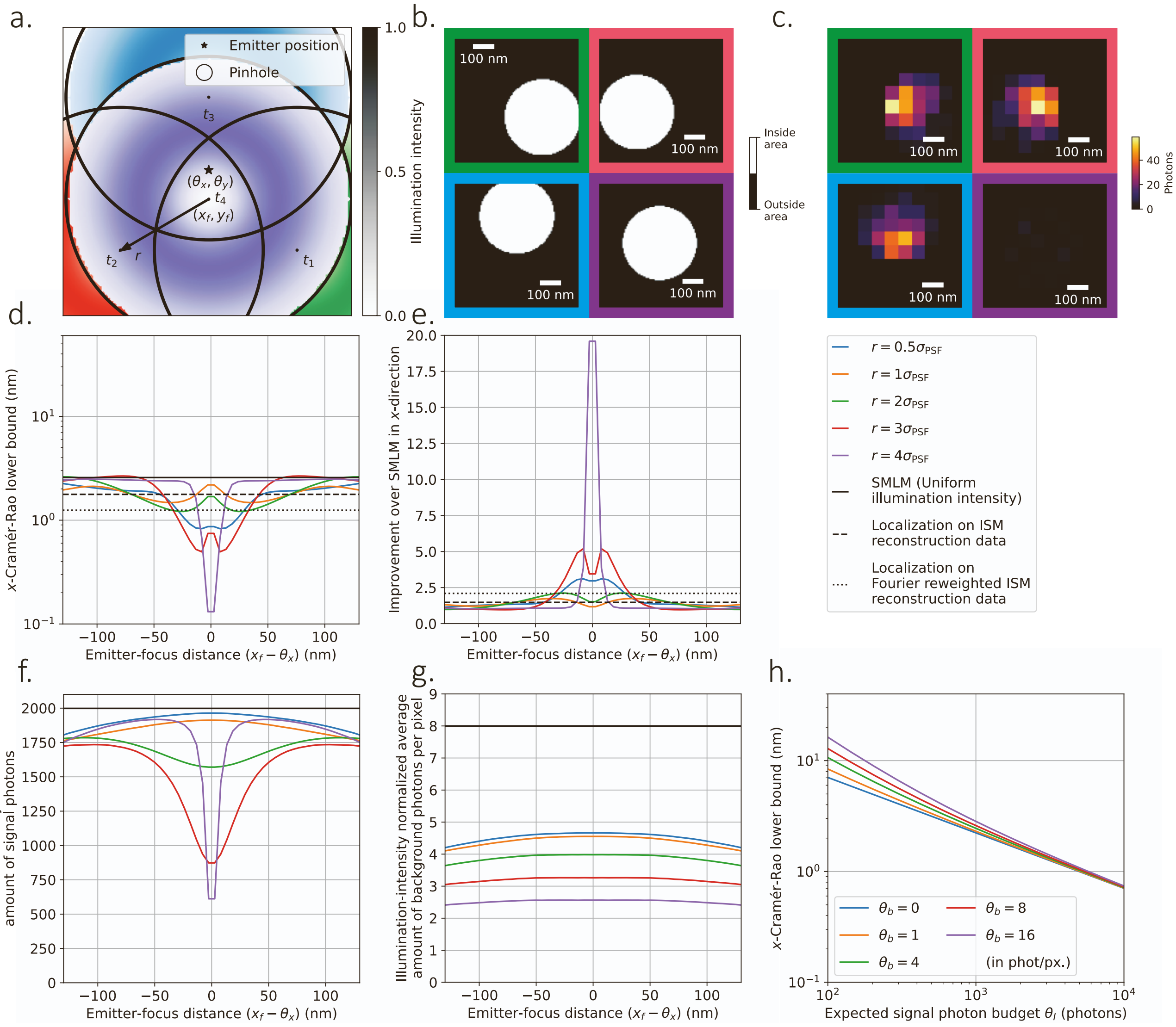

Figure S44: Theoretical minimum localization uncertainty of SpinFlux localization with four pinholes and donut-shaped patterns in an equilateral triangle configuration with a center pinhole. In (c-g), we used 2000 expected signal photons and 8 expected background photons per pixel, with pinhole radius $r_p = 3\sigma_{PSF}$. Results are evaluated for the scenario where the entire signal photon budget is exhausted after illumination with all patterns (disregarding signal photons blocked by the spinning disk), neglecting the effects of pattern-dependent background. **(a)** Schematic overview of SpinFlux localization with a triangle of three pinholes with an additional center pinhole, centered at focus coordinates (x_f, y_f). In (d-g), the x-distance $(x_f - \theta_x)$ between the pattern focus and the emitter is varied, where $y_f = \theta_y$. **(b)** Example of pinholes in the region of interest (650 × 650 nm). The pinhole radius $r_p = 2\sigma_{PSF}$ and pinhole spacing $r = 1.5\sigma_{PSF}$ were used. The pinhole masks were discretized with $N_{M,x}$, $N_{M,y} = 100$ mesh pixels in each direction. **(c)** Example of fluorescent response in the region of interest, resulting from illumination and emission through each pinhole in (b). **(d)** Cramér-Rao lower bound (CRLB) in x-direction as a function of the emitter-focus x-distance. Simulations show SpinFlux with varying pinhole spacing and widefield single molecule localization microscopy (SMLM). **(e)** Improvement of the SpinFlux CRLB over SMLM as a function of the emitter-focus x-distance for varying pinhole spacing. **(f)** Average amount of signal photons after compensation for non-maximum illumination intensity as a function of the emitter-focus x-distance, for SpinFlux with varying pinhole spacing and widefield single molecule localization microscopy (SMLM). **(g)** Average amount of background photons per pixel as a function of the emitter-focus x-distance, for SpinFlux with varying pinhole spacing and widefield single molecule localization microscopy (SMLM). **(h)** CRLB in x-direction as a function of expected signal photon count for varying values of the expected background photon count. The pinhole radius $r_p = 3\sigma_{PSF}$ and pinhole spacing $r = 2\sigma_{PSF}$ were used and $(x_f, y_f) = (\theta_x, \theta_y)$.

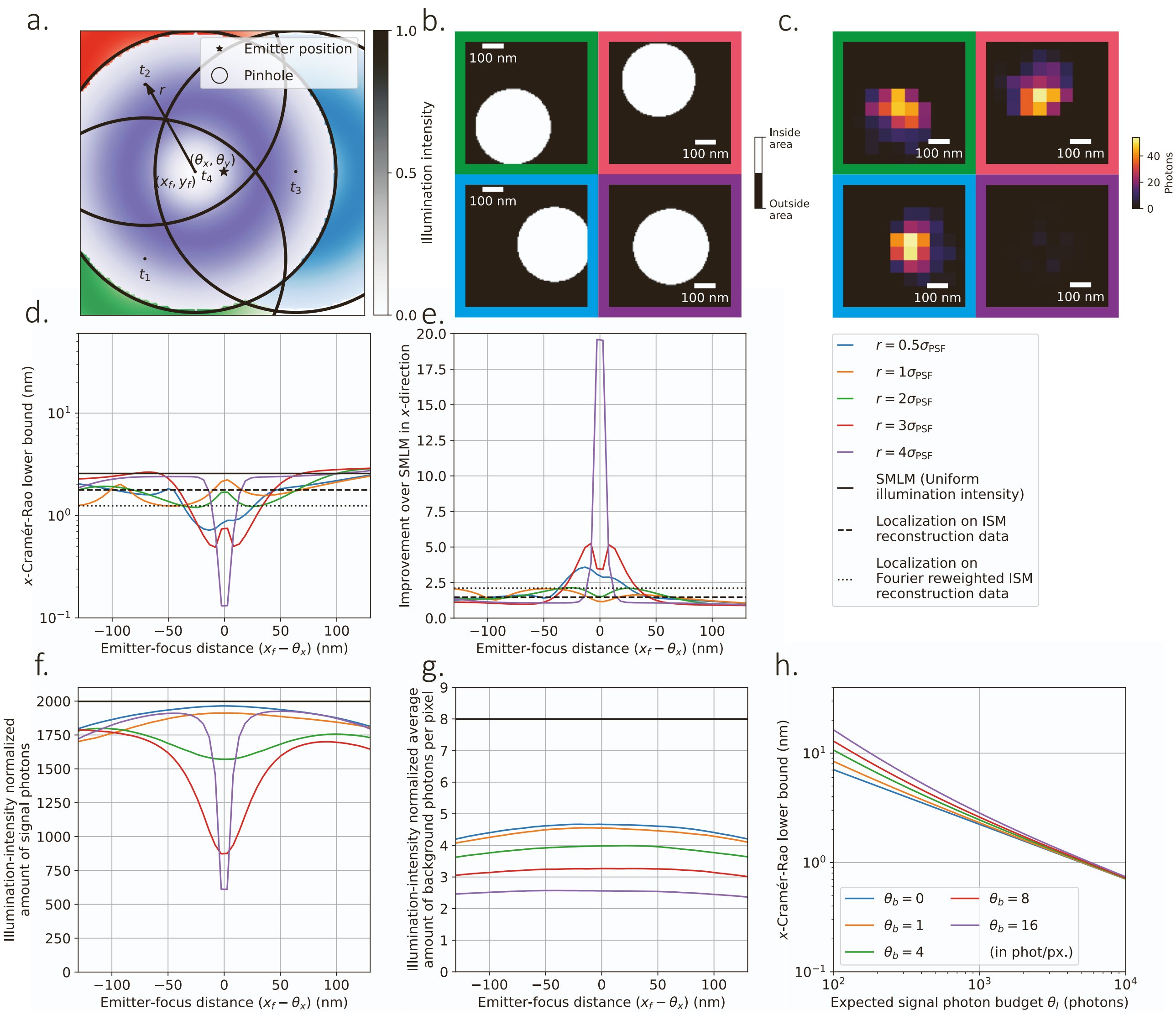

Figure S45: Theoretical minimum localization uncertainty of SpinFlux localization with four pinholes and donut-shaped patterns in a 90° rotated equilateral triangle configuration with a center pinhole. The pattern is rotated clockwise by 90 degrees with respect to Figure S40. In (c-g), we used 2000 expected signal photons and 8 expected background photons per pixel, with pinhole radius $r_p = 3\sigma_{PSF}$. Results are evaluated for the scenario where the entire signal photon budget is exhausted after illumination with all patterns (disregarding signal photons blocked by the spinning disk), neglecting the effects of pattern-dependent background. **(a)** Schematic overview of SpinFlux localization with a triangle of three pinholes with an additional center pinhole, centered at focus coordinates (x_f, y_f). In (d-g), the x-distance $(x_f - \theta_x)$ between the pattern focus and the emitter is varied, where $y_f = \theta_y$. **(b)** Example of pinholes in the region of interest (650 × 650 nm). The pinhole radius $r_p = 2\sigma_{PSF}$ and pinhole spacing $r = 1.5\sigma_{PSF}$ were used. The pinhole masks were discretized with $N_{M,x}$, $N_{M,y}$ = 100 mesh pixels in each direction. **(c)** Example of fluorescent response in the region of interest, resulting from illumination and emission through each pinhole in (b). **(d)** Cramér-Rao lower bound (CRLB) in x-direction as a function of the emitter-focus x-distance. Simulations show SpinFlux with varying pinhole spacing and widefield single molecule localization microscopy (SMLM). **(e)** Improvement of the SpinFlux CRLB over SMLM as a function of the emitter-focus x-distance for varying pinhole spacing. **(f)** Average amount of signal photons after compensation for non-maximum illumination intensity as a function of the emitter-focus x-distance, for SpinFlux with varying pinhole spacing and widefield single molecule localization microscopy (SMLM). **(g)** Average amount of background photons per pixel as a function of the emitter-focus x-distance, for SpinFlux with varying pinhole spacing and widefield single molecule localization microscopy (SMLM). **(h)** CRLB in x-direction as a function of expected signal photon count for varying values of the expected background photon count. The pinhole radius $r_p = 3\sigma_{PSF}$ and pinhole spacing $r = 2\sigma_{PSF}$ were used and $(x_f, y_f) = (\theta_x, \theta_y)$.

Table S1: Model parameters used in the SpinFlux localization precision simulations with Gaussian illumination patterns and with a Gaussian emission point spread function.

Quantity	Symbol	Value
Wavelength of excitation light	λ_{ex}	546 nm
Wavelength of emission light	λ_{em}	600 nm
Amount of pixels in x- and y-direction	N_x, N_y	10 pixels
Total amount of pixels	N_{pixels}	$N_x N_y$ = 100 pixels
Pixel size (x- and y-direction)	Δx, Δy	65 nm
Emitter x-position	θ_x	$\frac{N_{\text{pixels}}}{2}\Delta x = 325$ nm
Emitter y-position	θ_y	$\frac{N_{\text{pixels}}}{2}\Delta y = 325$ nm
Expected signal photon budget	θ_I	2000 photons
Expected background photon count	θ_b	8 photons/pixel
Numerical aperture	NA	1.35
Standard deviation of illumination PSF in x- and y-directions	σ_{illum}	$0.21\frac{\lambda_{\text{ex}}}{\text{NA}} = 84.9$ nm
Standard deviation of illumination PSF in x- and y-directions	σ_{PSF}	$0.21\frac{\lambda_{\text{em}}}{\text{NA}} = 93.3$ nm
Amount of discretization mesh pixels in x- and y-direction	$N_{x,M}$, $N_{y,M}$	100 pixels
Total amount of discretization mesh pixels	N_{M}	$N_{\text{M},x}N_{\text{M},y}$ = 10000 pixels
Discretization mesh pixel size (x-direction)	Δx_{M}	$\frac{N_x}{N_{\text{M},x}} \cdot \Delta x = 6.5$ nm
Discretization mesh pixel size (y-direction)	Δy_{M}	$\frac{N_y}{N_{\text{M},y}} \cdot \Delta y = 6.5$ nm

SUPPORTING REFERENCES

1. Schulz, O., C. Pieper, M. Clever, J. Pfaff, A. Ruhlandt, R. H. Kehlenbach, F. S. Wouters, J. Großhans, G. Bunt, and J. Enderlein, 2013. Resolution doubling in fluorescence microscopy with confocal spinning-disk image scanning microscopy. *Proceedings of the National Academy of Sciences* 110:21000–21005. https://doi.org/10.1073/pnas.1315858110.

2. Qin, S., S. Isbaner, I. Gregor, and J. Enderlein, 2020. Doubling the resolution of a confocal spinning-disk microscope using image scanning microscopy. *Nature Protocols* 16:164–181. https://doi.org/10.1038/s41596-020-00408-x.

3. Stallinga, S., and B. Rieger, 2012. The effect of background on localization uncertainty in single emitter imaging. *In* 2012 9th IEEE International Symposium on Biomedical Imaging (ISBI). 988–991.

4. Rieger, B., and S. Stallinga, 2014. The Lateral and Axial Localization Uncertainty in Super-Resolution Light Microscopy. *ChemPhysChem* 15:664–670. https://chemistry-europe.onlinelibrary.wiley.com/doi/abs/10.1002/cphc.201300711.

5. Smith, C. S., N. Joseph, B. Rieger, and K. A. Lidke, 2010. Fast, single-molecule localization that achieves theoretically minimum uncertainty. *Nat. Methods* 7:373–375. https://doi.org/10.1038/nmeth.1449.

6. Cnossen, J., T. Hinsdale, R. Ø. Thorsen, M. Siemons, F. Schueder, R. Jungmann, C. S. Smith, B. Rieger, and S. Stallinga, 2019. Localization microscopy at doubled precision with patterned illumination. *Nat. Methods* 17:59–63. https://doi.org/10.1038/s41592-019-0657-7.

7. Houwink, Q., D. Kalisvaart, S. Hung, J. Cnossen, D. Fan, P. Mos, A. C. Ülkü, C. Bruschini, E. Charbon, and C. S. Smith, 2021. Theoretical minimum uncertainty of single-molecule localizations using a single-photon avalanche diode array. *Opt. Express* 29:39920–39929. http://opg.optica.org/oe/abstract.cfm?URI=oe-29-24-39920.

8. Kalisvaart, D., J. Cnossen, S. Hung, S. Stallinga, M. Verhaegen, and C. S. Smith, 2022. Precision in iterative modulation enhanced single-molecule localization microscopy. *Biophysical Journal* 121:2279–2289. https://www.sciencedirect.com/science/article/pii/S0006349522004209.

9. De Luca, G. M., R. M. Breedijk, R. A. Brandt, C. H. Zeelenberg, B. E. de Jong, W. Timmermans, L. N. Azar, R. A. Hoebe, S. Stallinga, and E. M. Manders, 2013. Re-scan confocal microscopy: scanning twice for better resolution. *Biomedical Optics Express* 4:2644. http://dx.doi.org/10.1364/BOE.4.002644.

10. Mertz, J., 2019. Introduction to Optical Microscopy. Cambridge University Press. http://dx.doi.org/10.1017/9781108552660.

11. Pawley, J. B., 2006. Handbook Of Biological Confocal Microscopy. Springer US. http://dx.doi.org/10.1007/978-0-387-45524-2.

12. Slenders, E., and G. Vicidomini, 2023. ISM-FLUX: MINFLUX with an array detector. *Physical Review Research* 5. http://dx.doi.org/10.1103/PhysRevResearch.5.023033.

13. Balzarotti, F., Y. Eilers, K. C. Gwosch, A. H. Gynnå, V. Westphal, F. D. Stefani, J. Elf, and S. W. Hell, 2017. Nanometer resolution imaging and tracking of fluorescent molecules with minimal photon fluxes. *Science* 355:606–612. https://science.sciencemag.org/content/355/6325/606.

14. Sirinakis, G., E. S. Allgeyer, J. Cheng, and D. S. Johnston, 2022. Quantitative comparison of spinning disk geometries for PAINT based super-resolution microscopy. *Biomed. Opt. Express* 13:3773–3785. http://opg.optica.org/boe/abstract.cfm?URI=boe-13-7-3773.

15. Halpern, A. R., M. Y. Lee, M. D. Howard, M. A. Woodworth, P. R. Nicovich, and J. C. Vaughan, 2022. Versatile, do-it-yourself, low-cost spinning disk confocal microscope. *Biomed. Opt. Express* 13:1102–1120. http://opg.optica.org/boe/abstract.cfm?URI=boe-13-2-1102.

16. Kay, S. M., 1993. Fundamentals of Statistical Signal Processing, Volume I: Estimation Theory. Prentice Hall, Hoboken, NJ.

17. Ober, R. J., S. Ram, and E. S. Ward, 2004. Localization Accuracy in Single-Molecule Microscopy. *Biophys. J.* 86:1185–1200. https://doi.org/10.1016/S0006-3495(04)74193-4.
